# Moral and religious convictions: Are they the same or different things?

**DOI:** 10.1371/journal.pone.0199311

**Published:** 2018-06-21

**Authors:** Linda J. Skitka, Brittany E. Hanson, Anthony N. Washburn, Allison B. Mueller

**Affiliations:** Department of Psychology, University of Illinois at Chicago, Chicago, IL, United States of America; Universiteit van Amsterdam, NETHERLANDS

## Abstract

People often assume that moral and religious convictions are functionally the same thing. But are they? We report on 19 studies (*N* = 12,284) that tested whether people’s perceptions that their attitudes are reflections of their moral and religious convictions across 30 different issues were functionally the same (the *equivalence hypothesis*) or different constructs (the *distinct constructs hypothesis*), and whether the relationship between these constructs was conditional on political orientation (the *political asymmetry hypothesis*). Seven of these studies (*N* = 5,561, and 22 issues) also had data that allowed us to test whether moral and religious conviction are only closely related for those who are more rather than less religious (the *secularization hypothesis*), and a narrower form of the political asymmetry and secularization hypotheses, that is, that people’s moral and religious convictions may be tightly connected constructs only for religious conservatives. Meta-analytic tests of each of these hypotheses yielded weak support for the secularization hypothesis, no support for the equivalence or political asymmetry hypotheses, and the strongest support for the distinct constructs hypothesis.

## Introduction

People’s attitudes can be shaped by a number of different factors, including their preferences and tastes (“I hate licorice”), group norms or fads (“X is the new black”), or prudence (“look before you leap”). Some attitudes, however, are shaped or explained in terms of people’s religious beliefs. Forty-seven percent of a national sample of Americans who opposed same-sex marriage, for example, explained their opposition by referring to their religious beliefs and/or the bible [1]. The idea that people’s position on issues are sometimes based on or justified by religious belief is also inherent in the number of times Congress and various state legislatures have introduced legislation that makes exceptions based on the religious and moral convictions of the perceiver. For example, Senator Kennedy’s language in his Health Insurance Bill of Rights Act in 1997 stated, “A health insurance issuer may fully advise…enrollees…of the coverage’s limitations on providing particular medical services…based on the religious and moral convictions of the issuer” [2]. Statutes that make exceptions on the basis of “religious and moral convictions” seem to assume that religious and moral beliefs are essentially referencing the same underlying thing. But are they?

The goal of the 19 studies reported in this paper was to explore whether a perception that an attitude reflects one’s core moral or religious convictions reflects the same or different construct. Before turning to the specifics of these studies, we first briefly review our conceptualization of moral and religious conviction and review different theoretical positions on the (dis)connections between religious and moral attitudes and beliefs, as well as some research that has begun to explore the relationship between moral and religious convictions.

### Moral and religious conviction

People have a variety of perceptions or beliefs about their attitudes. Some attitudes, for example, are perceived or believed to be stronger, more extreme, certain, or important than other attitudes. These perceptions also seem to matter: Stronger, more extreme, certain and important attitudes are more likely to predict people’s attitudinally relevant thoughts, feelings, and behavior [3]. People not only have perceptions that some of their attitudes are comparatively strong, they also have perceptions that some attitudes are more strongly connected to their moral and/or religious beliefs as well. Debates about abortion, same sex marriage, stem cell research, capital punishment, prayer in public schools, and a host of other issues have constituencies that see these issues in terms of perceptions of right or wrong, in religious terms, or sometimes both [4, 5, 6]. That said, these issues themselves are not definitionally “moral” or “religious.” The issue of abortion, for example, was not always seen in moral or religious terms. Abortion services and drugs were marketed openly in the U.S. up until the late 1800s with little attention or protest. These practices only started to receive serious public and legal attention when the increasingly professionalized (and male) medical community started to replace other kinds of health care providers, that is, mostly female midwives and homeopaths [7]. Abortion is viewed as benignly acceptable as any other form of birth control in a variety of other cultural contexts, such as mainland China [8], the Czech Republic, Azerbaijan, Georgia, Moldovia, Romania, and Russia [9].

In addition to historical and national differences in the perceived moral acceptability of abortion, there is considerable individual variation in the American public about whether their views about abortion or other issues are rooted in personal moral conviction as well [6, 10], and similar if not more variability in the degree to which these attitudes reflect people’s religious convictions [6, 11]. Taken together, even though there is a tendency to treat some issues as if they are definitionally “moral” or “religious,” there is considerable historical, national, and individual differences in the degree to which these issues are in fact perceived in moral or religious terms.

It remains to be seen, however, whether people’s perceptions of the moral or religious relevance of an attitude co-vary, or whether these are distinguishable perceptions with perhaps different consequences and associations. Next, we review competing hypotheses about the connections or disconnections between people’s conceptions of morality and religion, that in turn have implications for whether people will perceive attitudes high in moral conviction to also be high in religious conviction and vice versa: The equivalence hypothesis, political asymmetry hypothesis, the secularization hypothesis, and the distinct constructs hypothesis [12].

### The equivalence hypothesis

The equivalence hypothesis posits that religion provides the motivational source of moral concerns. According to this view, morality and religion are nearly inseparably connected concerns. According to the equivalence hypothesis, morality is “a golden thread of humanitarianism inspired by loving care, motivated by religion” [13,14]. Because religion shapes people’s values, and these values in turn bolster people’s sense of purpose and meaning and indelibly shapes people’s conception of morality [15, 16, 17]. In a similar vein, past theory and research often discusses the moral and religious basis of certain attitudes as if these are interchangeable constructs [18, 19]. Most major religions (e.g., Buddhism, Christianity, and Judaism) also maintain that morality and faith are indivisibly related to each other [20]. People’s lay theories also reveal a strong tendency to assume tight connections between religious and moral belief as well: More than half of Americans believe that morality is impossible without a belief in God, a belief that is even stronger in a variety of other countries (e.g., Indonesia and Svengali [21]). There is also considerable evidence that people believe that a lack of religious belief is associated with immorality; even atheists are more likely to believe that religious non-believers are less moral than religious believers [22].

These lay beliefs are consistent with psychological explanations or formulations of the role and function of religiosity. Saroglou’s model of religious dimensions includes morality as a key pillar of religious belief, for example, arguing that “Religion not only is particularly concerned with morality as an external correlate but also includes morality as one of its basic dimensions” [23]. Evolutionary theorists similarly argue that religious and moral beliefs evolved as a set of interconnected cognitive systems that facilitate trust and cooperation within human groups, propositions that suggest that concerns about both are likely to be deeply intertwined [24]. If the equivalence hypothesis is true, then the degree to which someone sees their position on an issue as a reflection of moral conviction should strongly (if not nearly perfectly) predict whether they also see their position as a reflection of their religious convictions.

### The secularization hypothesis

The secularization hypothesis suggests that morality and religion have become increasingly separate overtime, especially in recent years as most societies are becoming increasingly secular. As societies become increasingly secular, public discourse is increasingly demoralized. Technical and legal mechanisms have progressively replaced the role of religion in facilitating cohesion and social control in much of social life [25, 26, 27]. The secularization hypothesis therefore suggests that morality and religion will be tightly associated only for the remaining people who are still religious. The connection between morality and religion, however, is increasingly fragmented for more and more people as religious belief continues to decline in the society at large. According to the secularization hypothesis, individual differences in religiosity and the degree to which people perceive an attitude as a reflection of religious conviction should interact to predict whether the attitude is also experienced as a moral conviction. Moral conviction and religious conviction should be more strongly correlated for people high, but not low, in religiosity.

### The political asymmetry hypothesis

Another possibility is that holding policy positions with both moral and religious convictions may be more likely for those on the political right than left. Consistent with this idea, religious Americans are more likely to have conservative than liberal positions on most issues [28, 29, 30, 31], something that suggests conservatives should be more likely to use their religious beliefs as a foundation for their political beliefs than will liberals. Although previous research has not found ideological differences in the tendency to have moral convictions about most issues [32], that does not mean that the factors that lead people to identify a given issue as a moral conviction will be the same for those on the political left and right. People on the political right might be more likely than those on the left to use religious beliefs not only as a cue for what position to take on a given issue, but also as a cue that an attitude is also a moral conviction. This prediction is consistent with the “political asymmetrys” framework for describing the contemporary American political climate [33] and with the notion that liberals’ and conservatives’ moral beliefs are based on quite different moral foundations [34]. The political asymmetry hypothesis is that religious conservatives and secular liberals possess radically “different systems of moral understanding,” [33], whereby religion informs conservatives’ political and moral beliefs to a much greater degree than it informs liberals’ beliefs [34]. In other words, the political asymmetry hypothesis (in its broad form) predicts that religious conviction will more strongly predict moral conviction for conservatives than it will for liberals (i.e., religious conviction and political orientation will interact to predict moral conviction for political issues).

### The distinct constructs hypothesis

A fourth theoretical perspective suggests that morality and religion are fundamentally different constructs. Kohlberg, for example, posited that religiosity and moral reasoning represented two disconnected areas of social and personal life [35]. People’s moral beliefs were thought to be based in rational arguments about justice that were affected by maturation and education and exposure to certain kinds of experiences. In contrast, Kohlberg argued that people’s religious beliefs were the internalized acceptance of religious knowledge and doctrine.

A key distinction between religious and moral beliefs is their comparative degree of authority independence: Religious beliefs are more intimately tied to authorities and rules than are moral beliefs [36, 37]. In other words, religious authorities and institutions teach their members what is acceptable or unacceptable, such as whether to eat pork or to go outside without covering one’s head. If religious authorities were reverse themselves and proclaim that is okay to eat pork or go hatless, their followers would very likely update their prior beliefs as well.

In contrast, people define moral beliefs in more absolutist terms that transcend what institutions or authorities dictate [12, 36, 37]. If, for example, someone has a moral commitment to the idea that eating meat is morally wrong, it would not matter what authorities or the law had to say about the practice: The perceiver would still see meat consumption as wrong. In summary, the distinct constructs hypothesis is that moral convictions operate independently of all concerns about authority, whereas religious convictions are to a very considerable degree authority dependent (e.g., obeying God or religious doctrine). In contrast to the equivalence hypothesis, the distinct construct hypothesis is that moral and religious convictions should not share a great deal of common variance.

We should also note that our hypotheses are not necessarily mutually exclusive. One possibility, for example, is that the secularization and political asymmetry hypotheses could both be true. That is, we might observe a strong connection between moral and religious convictions about a given issue only among religious conservatives, for example, and not among secular conservatives, in addition to secular and religious liberals. If both the secularization and political asymmetry hypotheses are true, we would predict a three-way interaction between political orientation, religiosity, and religious conviction predicting moral conviction about any given issue, in other words, support for both of these hypotheses is consistent with what we will call the narrower form of the political asymmetry and secularization hypotheses.

### The state of existing knowledge

Although generally not designed to explicitly test the hypotheses proposed here, there has been some limited study of the connections between moral and religious convictions. Wisneski, Lytle, and Skitka [38], for example, found that religious and moral convictions about physician-assisted suicide (PAS) were correlated at *r* (650) = .32, *p* < .01, suggesting that these constructs share only about 10% common variance. Religious and moral conviction about PAS in the same sample uniquely predicted participants’ trust or distrust in the Supreme Court to make the right decision about whether to allow this practice in the United States: stronger moral convictions about PAS were uniquely associated with greater distrust in the Supreme Court to get PAS right and stronger religious convictions about PAS were associated with greater trust in the Court to get this issue “right”.

Moral and religious convictions about PAS also had unique effects on people’s subsequent decision acceptance of an actual Supreme Court decision to uphold Oregon’s Death with Dignity Act [12]. Moral convictions about PAS also predicted changes in the perceived legitimacy of the U.S. Supreme Court from pre- to post ruling on its decision to uphold Oregon’s Death with Dignity Act. People with stronger moral convictions in support of legalizing PAS saw the Supreme Court as higher, and those who were morally opposed to PAS saw the Supreme Court as lower in legitimacy than they did pre-ruling. In contrast, despite the fact that those with stronger religious convictions about PAS uniformly opposed the Court’s decision, their beliefs about the legitimacy of the Supreme Court were unaffected by the Court’s decision. Because religious and moral convictions were only weakly correlated and related differently to other variables (the Court’s legitimacy), these results are not very consistent with the equivalence hypothesis. Because religiosity and political orientation were not explored as possible moderators of these effects, however, the political asymmetry and the secularization hypotheses can be ruled out by this study.

Morgan, Skitka and Wisneski [11] found a stronger correlation between people’s moral and religious convictions associated with their self-selected most important issue in the 2008 presidential election, *r*(416) = .44, *p* < .001. Although the correlation was stronger, it was not sufficiently high to suggest that moral and religious convictions about people’s most important issues are necessarily the same construct. Religious and moral convictions about people’s most important issue also had different associations with intentions to vote. Stronger moral convictions predicted stronger intentions to vote, whereas stronger religious convictions predicted weaker intentions to vote in that election. Both results were unqualified by party identification, a result at odds with the political asymmetry hypothesis [11].

In summary, although there is some support in favor of the distinct constructs view of moral and religious conviction, the hypothesis has only been tested in a handful of attitude domains, and research has not ruled out whether there are contingent associations between moral and religious convictions, that is, whether this relationship might be stronger for those who are more religious and/or conservative. The goal of this research was to test these competing hypotheses across a wide range of issues and a variety of different samples.

## Method

The University of Illinois at Chicago Institutional Review Board reviewed this research and deemed it exempt, approval #2016–1105.

### Samples, issues, and replications

Our lab has been collecting ancillary data on both moral and religious conviction for years, but it has for the most part gone unanalyzed because the moral/religious distinction was not the main focus of those projects. The current research involved returning to every sample we had access to that had at least some of the relevant variables to allow us to test the hypotheses outlined above. We identified 19 different studies/samples that included measures of political orientation, and moral and religious convictions with a total *N* of 12,284 that allowed us to examine the relationship between political orientation and moral and religious convictions across 30 different issues. Because the same issues were used in more than one study/sample, we also were able to test the replicability of support or non-support for the equivalence, political asymmetry, and distinct constructs hypotheses with the issues of abortion (3 studies/samples), capital punishment (2 studies/samples), gun control (4 studies/samples), nuclear power (2 studies/samples), physician assisted suicide (2 studies/samples), same-sex marriage (4 studies/samples), and workplace professionalism (2 studies/samples).

Only some of the 19 studies/samples, however, also included a measure of religiosity. We were able to test the secularization and narrow political asymmetry hypotheses, therefore, only with a smaller set of issues and participants. Tests of the secularization and narrow political asymmetry hypotheses were conducted with 7 different studies/samples with a total *N* of 5,561 across 22 issues. We were also able to examine the replicability of some of these observed effects because the same issues were examined in more than on study/sample: abortion (2 studies/samples), physician assisted suicide (2 studies/samples), and workplace professionalism (2 studies/samples).

Most of our samples were U.S. Mechanical Turk workers. That said, our samples also often included college students recruited from psychology subject pools; students, faculty, and staff recruited from a university listserv; two national samples (one true probability sample in the former case, and one quota sample) and two community samples of non-U.S. participants (mainland Chinese, *N* = 207, and Israeli Jews, *N* = 136). More specific details about each of our samples is provided in S1 File and all data is available in S1 Datasets.

## Measures

All studies included a measure of moral conviction, religious conviction, and political orientation. As noted above, only some studies also included a measure of religiosity. More details about the specific measurement for each study is provided in S1 File.

### Moral conviction

Moral conviction associated with attitude objects was measured with between 1 and 4 items. Typical items asked the degree to which participants’ attitude about a given object was “a reflection of your core moral beliefs and convictions,” “connected to your beliefs about fundamental right and wrong,” “based on moral principle,” and “a moral stance” typically on 5-point scales labeled *not at all*, *slightly*, *moderately*, *much*, and *very much* (multi-item measures α ranged from .71 to .90); single-item or two-item measures generally consisted of the first and second items on this list.

### Religious conviction

Religious conviction was generally measured with a single face-valid item, specifically, the extent to which participants indicated that their attitude about [given issue] was “a reflection of your religious beliefs and convictions,” and in a small number of cases, was supplemented with an item that asked the degree to which their attitude was “a religious stance.” In most cases, the same response options were used for moral conviction as described above. The religious and moral conviction items were interspersed with other items assessing attitude strength (e.g., attitude certainty, importance) or were presented in completely different parts of the survey, which should have reduced any demand to respond to these items similarly.

### Religiosity

We used three items from the Santa Clara Strength of Religiosity scale to measure religiosity [39]. Participants were asked how much each of the following states described them: “My religious faith is extremely important to me,” “My religious faith impacts many of my decisions,” and “I look to faith for meaning and purpose in my life,” with the response options generally of *not at all*, *slightly*, *moderately*, *much*, and *very much* (α ranged from .95 to .98 across samples). Religiosity was usually measured at the end of the survey in concert with demographic characteristics, and separately from the attitude items.

### Political orientation

Participants in most samples were given two items to assess their political orientation (see S1 File for exceptions). First, participants answered the question “Generally speaking, do you usually think of yourself as a liberal, conservative, moderate, or something else?” Participants who reported being either liberal or conservative were branched to a second item asking them the degree to which they considered themselves liberal (conservative) using a 3-point scale with the point labels *slightly*, *moderately*, and *strongly liberal (conservative)*. Participants who indicated being something other than liberal or conservative for the first question were branched to a second item that asked “If you had to choose, would you consider yourself a liberal or a conservative?” with the response options *liberal*, *conservative*, and *neither*. We typically recoded participants’ responses to the two items they received into a single, 7-point bipolar measure of political orientation with higher numbers indicating greater conservatism/less liberalism and those who reported leaning toward liberal or conservative recoded into the “slightly” category for each group, although in some cases it was scored as nine-point scale (with the leaners coded a point lower than, rather than being folded into “slightly”).

## Results

We used the following analytic strategy all 19 studies. Participants from each study were included in analyses if they provided data for at least two of the key variables of interest (moral conviction, religious conviction, political orientation, and religiosity). Any missing values were replaced with the mean of the sample. Sample sizes sometimes vary slightly across issues within the same study, however, due to different patterns of missingness for variables included in the analysis, due to some examples of people not completing any measures for a given issue (e.g., the participant dropped out before completing the entire survey).

We first calculated the bivariate correlations between moral conviction, religious conviction, political orientation, and religiosity for each issue to test the equivalence and distinct construct hypotheses. To test the political asymmetry hypothesis in the subset of studies that only measured moral conviction, religious conviction, and political orientation, we ran a two-step hierarchical regression model predicting moral conviction. The direct effects of religious conviction (mean centered) and political orientation (midpoint centered) were entered in the first block and the interaction of these two predictor variables were entered in the second block. To test the broad secularization hypothesis as well as the narrow form of secularization and political asymmetry hypotheses in the subset of studies that also measured religiosity, we ran a three-step hierarchical regression model predicting moral conviction. The direct effects of religious conviction (mean centered), political orientation (midpoint centered), and religiosity (mean centered) were entered in the first block, all two-way interactions and the three-way interactions of these predictor variables were entered in the second and third block, respectively. All significant interactions were followed up at one standard deviation above the mean, the mean, and one standard deviation below the mean of religiosity and religious conviction, and one standard deviation above the midpoint, the midpoint, and one standard deviation below the midpoint of political orientation where appropriate (see S1 File for all analyses). Hypotheses were then tested using random effects meta analyses using the metafor package in R [40]. We report the meta-analytic results below.

### The equivalence and distinct construct hypotheses

The equivalence hypothesis predicts that moral and religious convictions are functionally the same constructs, whereas the distinct construct hypothesis predicts that moral and religious convictions are relatively orthogonal. Statistically, the former hypothesis implies that self-reported moral and religious convictions for any given issue should be highly correlated. We *a priori* defined “highly correlated” as above *r* = .70, or approximately 50% shared variance. Although this cut-off for support for the equivalence hypothesis was agreed to before we knew the findings, it was a pre-registered hypothesis only for Study 19 (see https://aspredicted.org/fd86k.pdf). Results were more consistent with the distinct constructs than the equivalence hypothesis.

**Fig 1:**
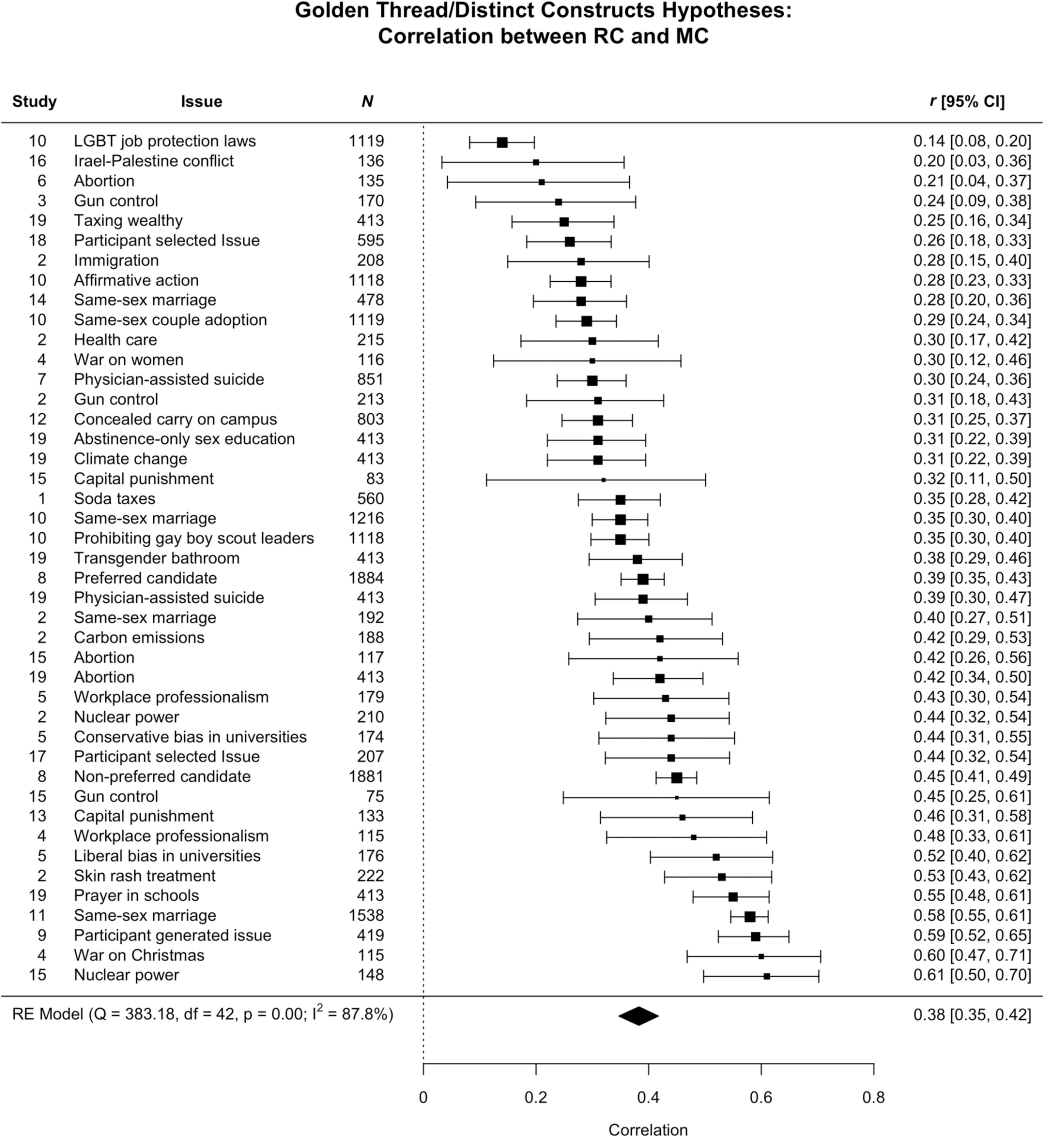
Correlations (*r*) between moral and religious convictions for each issue as well as the meta-analytic correlation.

Fig 1 summarizes the tests of the equivalence and distinct constructs hypotheses (see S1 File for analyses used for inputs into the meta-analysis). The correlation between moral and religious conviction showed considerable variability across issues and samples (*r* = .14 for protections against discrimination for lesbian, gay, bisexual and transsexual, or LBGT individuals, to a *r* = .61 for building new nuclear power plants in the U.S.), and yielded a meta-analytic *r* = .38, *p* < .001, 95% CI [.35, .42] (or 14% shared variance). Not one correlation between moral and religious conviction was at or greater than .70. These results suggest that even if people’s perceptions that their attitudes reflect their moral and religious convictions are correlated at higher than non-zero levels, the degree of association between these variables is nonetheless not sufficiently large to support the hypothesis that these meta-perceptions of attitudes are based on a common golden thread. These results are instead more consistent with the distinct constructs than the equivalence hypothesis.

### The broad political asymmetry hypothesis

**Fig 2:**
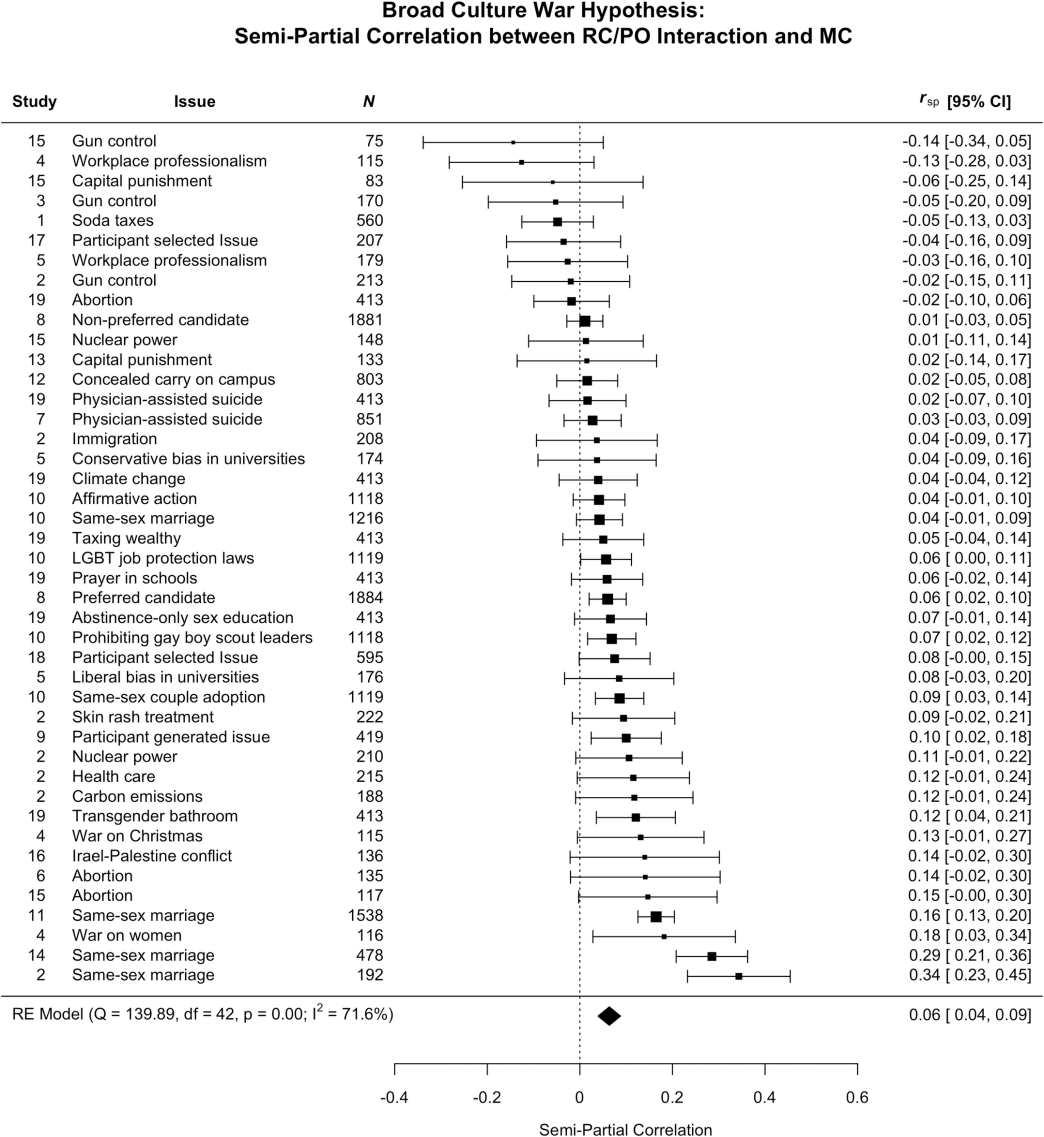
Effect sizes (*r*) represent the semi-partial correlation between the religious conviction / political orientation and moral conviction for each issue and the overall weighted meta-analytic effect.

We interpreted the results as consistent with the broad political asymmetry hypothesis if we observed a two-way interaction between religious conviction and political orientation, such that religious and moral conviction was positively correlated for conservatives, but not (or more weakly) correlated for liberals. The broad political asymmetry hypothesis prediction that there would be a stronger association between moral and religious conviction for conservatives than liberals was supported for 7 out of 30 issues (preferred presidential candidate, prohibiting gay boy scout leaders, same-sex couple adoption, participant generated issue, allowing transgendered people to use the bathroom of their choice, same-sex marriage, and the “war on women”), or 23% of the issues tested). One of these supportive results (same-sex marriage) also replicated in two different samples (Fig 2).

For the meta-analysis, the test of this hypothesis was represented by the semi-partial correlation between the religious conviction and political orientation interaction term, and moral conviction controlling for the direct effects of religious conviction and political orientation (see S1 File for the hierarchical regressions and follow-ups of any significant interactions). On the whole, how and whether moral and religious convictions co-vary was not explained well by the political asymmetry hypothesis, meta-analytic semi-partial *r* = .06, or well less than 1% shared variance.

### The secularization hypothesis

**Fig 3:**
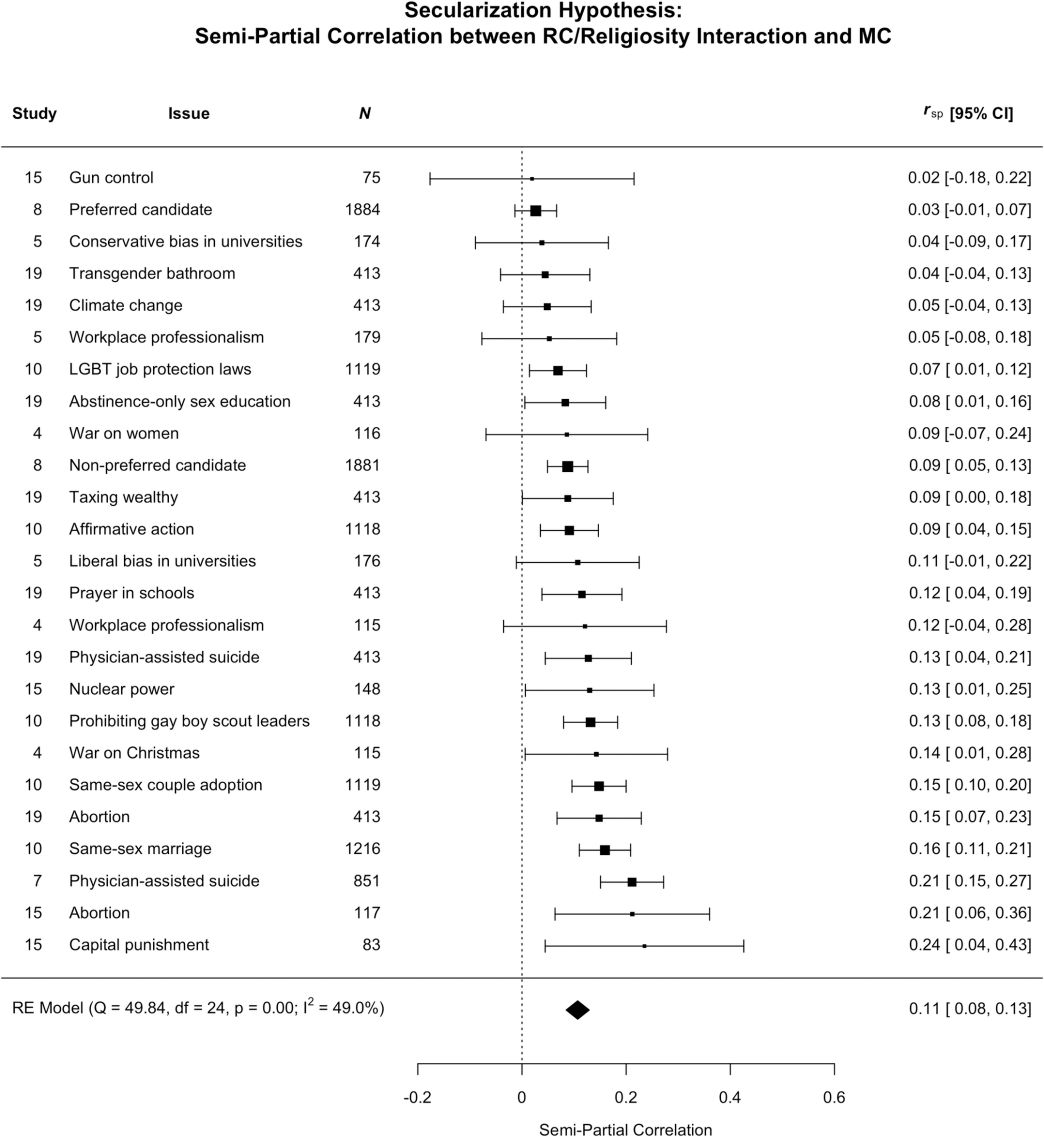
Effect sizes (*r*) represent the semi-partial correlation between the religious conviction / religiosity interaction and moral conviction for each issue and the overall weighted meta-analytic effect.

The secularization hypothesis predicted a two-way interaction between religiosity and religious conviction to predict moral conviction. If the secularization hypothesis is true, then moral and religious conviction should be more strongly correlated for people high than low in religiosity. We found some support for this hypothesis (Fig 3). The confidence intervals for the effect size of the semi-partial correlation between the religious conviction by religiosity interaction (controlling for the direct effects of both variables) predicting moral conviction did not include zero for 13 out 22 issues examined (or 59%), with a meta- semi-partial *r* = .11 (or just over 1% shared variance). Analyses of simple slopes of significant interactions were also consistent with the pattern predicted by the secularization hypothesis (see S1 File).

Although the overall effect size for the religiosity by religious conviction interaction was very small, it also replicated well across 2 out of the 3 tests possible (abortion and physician assisted suicide, but not workplace professionalism).

### The narrow political asymmetry hypothesis

**Fig 4:**
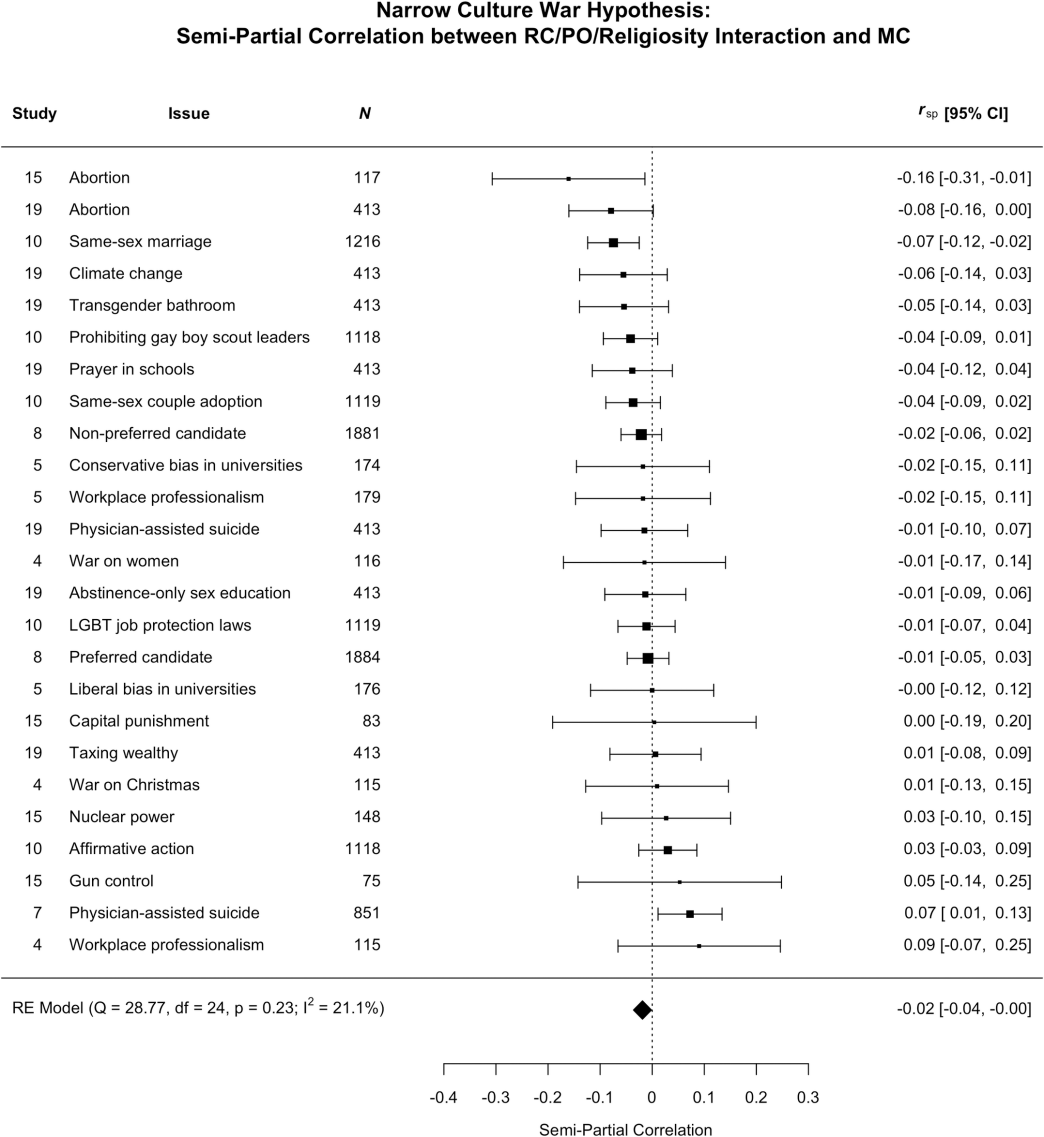
Effect sizes (*r*) represent the semi-partial correlation between the religious conviction, religiosity, and political orientation interaction and moral conviction for each issue and the overall weighted meta-analytic effect.

We also acknowledged the possibility that the data could support both the political asymmetry and the secularization hypotheses. In other words, it might be the case that moral and religious conviction are only strongly correlated for religious conservatives. This hypothesis would be supported if we observed a three-way interaction between religiosity, religious conviction, and political orientation predicting moral conviction. We found no support for the narrower version of the political asymmetry and secularization hypotheses (Fig 4). We found only one effect with a confidence interval that did not include zero (physician assisted suicide in one sample), a finding that that did not replicate in a second sample. The meta-analytic semi-partial was *r* = -.02, *p* = .033, 95% CI [-.04, -.002], or .04% shared variance.

## Discussion

The goal of this study was to test competing hypotheses about the relationship between two kinds of perceptions people sometimes have about their attitudes, specifically the perception that a given attitude is rooted in or reflects their moral and/or religious convictions. People’s lay theories often confound these constructs: If something is perceived as religious, it will also be perceived as moral (and vice versa). Contrary to both people’s lay theories and various scholarly theories of religion, however, we found that the degree to which people perceive a given attitude as a moral or religious conviction is largely orthogonal, sharing only on average 14% common variance.

We also tested whether the relationship between moral and religious convictions might be stronger under some conditions or for some people, such as people higher in religiosity and/or conservatives. We found no strong support for the latter and some support for the former hypothesis. Religious and moral conviction were more strongly related to each other among the religious than the non-religious for 59% of the issues we examined, a finding consistent with the secularization hypothesis. That said, the effect size in support of the secularization hypothesis was very small; the interaction of religiosity and religious conviction only explained a little more than 1% of the variance in moral conviction overall. Taken together, the overwhelming evidence therefore seems most consistent with the distinct constructs hypothesis: Moral and religious convictions are largely independent constructs.

The conclusion in support of the distinct constructs hypothesis is bolstered by the fact that we set the burden of proof for the equivalence hypothesis very low (50% shared variance or higher), we considered a broad range of plausible alternative hypotheses (the broad and narrow political asymmetry and secularization hypotheses), and we tested hypotheses across dozens of samples and issues, and therefore had a very large meta-analytic sample size. Moreover, our meta-analytic effect size estimates are very unlikely to be inflated or biased due to publication biases, because in all but two samples, nothing about the relationship between moral and religious conviction had been previously published. To our knowledge, our lab is the only one that has been collecting data about religious convictions related to attitudes, so this meta-analysis very likely includes all studies of religious conviction conducted to date—in short, this meta-analysis is also free from the file drawer problem [41]. Finally, we observed very similar conclusions in our two non-U.S. samples as we did in our U.S. samples. The correlation between moral and religious conviction about the Israeli-Palestinian conflict on our Jewish Israeli sample, for example, was among the weakest we observed, and even weaker than we observed in our mainland China sample.

The conclusion that moral and religious convictions are distinguishable constructs is also consistent with studies that find that that these variables have very different relationships with outcome variables of interest. Both moral and religious conviction associated with people’s attitudes about physician assisted suicide, for example, predicted how fair and acceptable the Supreme Court’s decision was in a case that challenged state’s rights to legislate the legality of this practice in *Gonzales v*. *Oregon*. People with strong moral convictions thought that the Supreme Court’s decision in this case was more fair or unfair, or more acceptable or unacceptable as function of whether the Court’s ruling was consistent with their moralized preferences to either legalize or prohibit physician assisted suicide. In contrast, the more religiously convicted (who mostly oppose the practice) found the Court’s ruling in favor of Oregon’s Death with Dignity Act as more unfair and unacceptable than those who were less religiously convicted about the issue (note: moral and religious conviction did not interact to predict any outcome variable in this study).

Where the morally convicted and religiously convicted converged, however, was in their subsequent impressions of the legitimacy of the Supreme Court. Although the religiously convicted clearly did not like the Court’s decision, their perceptions of the Supreme Court’s legitimacy did not change from pre- to post its ruling in this case [12]. People with strong moral convictions about physician assisted suicide, however, did see the legitimacy of the Supreme Court differently as a function of whether the Court yielded a morally pleasing or displeasing decision. In essence, people used their moral beliefs about physician assisted suicide as a litmus test of the legitimacy of the Supreme Court: The Court was seen as even more legitimate after ruling in favor of the Oregon’s Death with Dignity Act by morally convicted supporters but was seen as less legitimate by morally convicted opposers of physician assisted suicide after than before the Court ruled in this case [12].

Moral and religious convictions associated with people’s most important issues in the 2008 Presidential election cycle also had very different relationships with whether people voted in that election. Higher levels of moral conviction about issues in the election that year were associated with increased intentions to vote. Stronger religious convictions on the same issues, however, were associated with reduced intentions to vote [11].

Importantly, the finding that moral and religious convictions are distinct constructs is consistent with moral domain theory predictions [36, 37]. According to domain theory, one key way that moral and religious beliefs differ is in the degree to which they are dependent on authorities. When people say that a political position they hold reflects their religious convictions, they are likely to be communicating that they take the position that is supported by religious elites within their faith communities, and/or their understanding of religious texts like the bible or the Koran. In other words, these attitudes are *de facto* based on authority. When people say that their attitude reflects a moral conviction, in contrast, they are communicating something they believe independent of what authorities or even their peers have to say about the matter. That moral convictions have the property of authority and peer independence is the most robustly replicated effect of the moral conviction research program [12, 42, 43, 44, 45, 46].

Future research should begin to further explore the implications of religious convictions, and how these might differ from moral convictions. Moral convictions, for example, are associated with greater social, and sometimes also with greater political, intolerance of attitudinally dissimilar others [6, 47]. If domain theory predictions about the differences between religious and moral belief are true, then one would expect these effects would be weaker or non-existent with respect to religious convictions. Because people tend to feel that their moral convictions are objectively and universally true [48] people who do not share them are clearly suspect and do not belong to one’s moral community. Religious convictions, however, are likely to be experienced as something shared within one’s faith community, but not necessarily shared with those outside of that community. People may therefore be more tolerant of religious than they are of moral differences.

Similarly, people also have difficulty accepting compromise with respect to issues they hold as strong moral convictions [6, 49]. Would they have the same difficulty when asked to consider compromises with respect to issues they hold as strong religious convictions, that are not also high in moral convictions? It is possibly that some of the negative stereotypes about religious beliefs—for example, that they can be dogmatic or unyielding—might not be about religiosity at all but might be the consequence of strong moral convictions. Discovering that moral and religious convictions are largely orthogonal constructs opens the door to a whole range of possible research that explores how each of these variables uniquely relates to a host of phenomena including social and political (in)tolerance, compromise, political engagement, pro-social and anti-social behavior, social influence and persuasion, as well as a host of other possibilities.

Before closing, we should note that our conclusions are limited to our nearly exclusive reliance on U.S. samples at a particular point in time. It is possible that religious and moral convictions could have been much more highly correlated at earlier times in history, when U.S. was less secularized. Our results might also have looked very different if we examined samples where there is almost universal agreement that morality is not possible without God (e.g., if we used Indonesian or Svengali samples instead of a primary reliance on U.S. samples).

Finally, we should note that the conclusions we can make with the studies examined here are limited to how people’s moral and religious convictions are related to their attitudes. Morality and religiosity may have other ties that we are not capturing with this research. Religion and religious belief, for example, may play a stronger role in encouraging moral traits and/or behavior through socialization and internalization of moral norms. The degree to which there is in fact a tie between religious belief and morality, however, often does not withstand empirical inquiry. One study that assessed people’s everyday moral and immoral experiences, for example, found that the religious and nonreligious were not different in either the likelihood or quality of committed moral and immoral acts [50]. Other studies similarly find weak or no evidence of an association between prosociality and religiosity [51]. An extensive study of children’s prosociality across six countries (Canada, China, Jordan, Turkey, United States, and South Africa), for example, surprisingly found that children raised non-religious households were more altruistic than children raised in religious households [52], or that there are no differences in altruism as a function of religiosity [53]. Taken together, many people clearly believe that morality and religious belief go together. Empirical investigation of the veracity of this longstanding belief, however, suggests something different.

## Supporting information

S1 File File provides detailed descriptions of the methods and results for the 19 studies reported in this manuscript. (DOCX)

S1 Datasets This zip folder includes subfolders by study with data and analysis scripts. (ZIP)
