# Supplementary material for: Moral and religious convictions: Are they the same or different things?
Source: PLoS One. 2018 Jun 21;13(6):e0199311. doi: 10.1371/journal.pone.0199311 (PMC6013213; doi:10.1371/journal.pone.0199311)
Supplement: S1 File — (DOCX) [file pone.0199311.s001.docx]

# General Analytic Strategy

For all studies presented in this supplemental material we used the following analytic strategy. Participants from each study were included in analyses if they responded to at least two of the key variables of interest (moral conviction, religious conviction, political orientation, and religiosity). Any missing values were replaced with the mean of the sample.^[[1]](#footnote-1)^ We first calculated the bivariate correlations between moral conviction, religious conviction, political orientation, and religiosity for each issue to test the golden thread and distinct construct hypotheses. Next, to test the broad culture war hypothesis in the subset of studies that only measured moral conviction, religious conviction, and political orientation, we ran a two-step hierarchical regression model predicting moral conviction. The direct effects of religious conviction (mean centered) and political orientation (midpoint centered) were entered in the first block and the interaction of these two predictor variables were entered in the second block. To test the secularization hypothesis as well as both the broad and narrow form of the culture war hypotheses in the subset of studies that also measured religiosity, we ran a three-step hierarchical regression model predicting moral conviction. The direct effects of religious conviction (mean centered), political orientation (midpoint centered), and religiosity (mean centered) were entered in the first block, all two-way interactions and the three-way interaction of these predictor variables were entered in the second and third block, respectively. All significant interactions were followed up at one standard deviation above the mean, the mean, and one standard deviation below the mean of religiosity and religious conviction, and one standard deviation above the midpoint, the midpoint, and one standard deviation below the midpoint of political orientation where appropriate.

# Study 1: Washburn & Skitka, 2015

## Method

This study was approved by the Institutional Review Board of the Office for the Protection of Research Subjects at the University of Illinois at Chicago. Prior to completing any study measures, participants were presented with an informed consent form with information about the study and consented to participate.

### Participants

Participants were recruited from UIC’s Massmail email system starting on August 17, 2015. Our sample consisted of 560 participants who completed relevant measures. Participants ranged in age from 18 to 73 (*M* = 28.80, *SD* = 12.45) and were 60% female and 38% male. Fifteen percent had a high school diploma or GED, 22% had some college but no degree, 9% had a technical/associates degree, 26% had a bachelor’s degree, 18% had a master’s degree, and 10% had a doctoral/professional degree.

### Measures

Participants completed measures of moral and religious conviction (interspersed with other items) about their attitudes on soda taxes. They also completed a measure of political orientation.

**Moral conviction.** Moral convictions associated with their soda taxes attitude were measured with a validated two item measure. Participants rated the degree to which their attitudes were “connected to your beliefs about fundamental right and wrong,” and “a reflection of your core moral beliefs and convictions” on 5-point scales labeled *not at all, slightly, moderately, much,* and *very much* (α = .76).

**Religious conviction.** Religious conviction was a measured with a single face-valid item, specifically, the extent to which participants indicated that their attitudes were “connected to their religious beliefs and convictions,” with the same response options we used for moral conviction. The religious and moral conviction items were interspersed with other items assessing attitude strength (e.g., attitude certainty, importance).

**Political orientation.** Political orientation was measured by asking participants whether they generally think of themselves as liberal, conservative, or uncertain. Participants who indicate whether they are liberal or conservative subsequently branched to an item that asked how strongly they identified as liberal or conservative, with the response options of *slightly, moderately, much, and very much.* Participants who response with “uncertain” branched to an item that asked whether they leaned more toward liberal or conservative, or neither. Leaners were recoded as either *slightly liberal* or *slightly conservative*, and responses to these combined items were used to create a 9-point measure of political orientation, with higher scores reflecting greater conservativism.

## Results

As can be seen in Table S1.1 and in support of our distinct constructs hypothesis, moral and religious conviction for attitude positions on soda taxes were only moderately correlated. As can be seen in Table S1.2, the relationship between religious conviction and moral conviction was not moderated by political orientation, providing no support for our broad culture war hypothesis.

| Table S1.1  *Descriptive Statistics and Bivariate Correlations for Soda Taxes* | | | | | | |
| --- | --- | --- | --- | --- | --- | --- |
|  |  | *M* | *SD* | (1) | (2) | (3) |
| (1) | Political Orientation | -1.32 | 1.80 | - |  |  |
| (2) | Religious Conviction | 1.26 | 0.73 | .14** | - |  |
| (3) | Moral Conviction | 2.38 | 1.18 | .07 | .35** | - |
| *Note.* * *p* < .05. ** *p* < .01. Higher scores on political orientation indicate greater conservatism. | | | | | | |

| Table S1.2  *Hierarchical Regression Model Predicting Soda Tax Moral Conviction* | | | | |
| --- | --- | --- | --- | --- |
|  |  | Moral Conviction | |  |
|  | Predictor | *B* | *SE* | Δ*R*^2^ |
| Block 1  df = 557 | Political Orientation | 0.01 | 0.03 |  |
|  | Religious Conviction | 0.57** | 0.06 | .13** |
| Block 2  df = 556 | Political Orientation X Religious Conviction | -0.04 | 0.04 | .002 |
| *R*^2^ |  |  |  | .13** |
| *Note*. * *p* < .05. ** *p* < .01. Higher scores on political orientation indicate greater conservatism. | | | | |

# Study 2: Washburn & Skitka, 2017

## Method

This study was approved by the Institutional Review Board of the Office for the Protection of Research Subjects at the University of Illinois at Chicago. Prior to completing any study measures, participants were presented with an informed consent form with information about the study and consented to participate.

### Participants

Participants were recruited from Amazon’s Mechanical Turk website (Mturk) starting on August 13, 2014. Our sample consisted of 1488 participants who completed relevant measures. Participants ranged in age from 18 to 80 (*M* = 36.08, *SD* = 14.41) and were 54% male and 46% female. Less than one percent had less than a high school education, 12% had a high school diploma or GED, 24% had some college but no degree, 12% had a technical/associates degree, 33% had a bachelor’s degree, 15% had a master’s degree, and 4% had a doctoral/professional degree.

### Measures

Participants completed measures of moral and religious conviction (interspersed with other items) about their attitudes toward skin rash treatments, gun control, carbon emissions, nuclear power, same-sex marriage, health care, or immigration. They also completed a measure of political orientation.

**Moral conviction.** Moral convictions associated with each attitude were measured with a validated two item measure. Participants rated the degree to which their attitudes were “connected to your beliefs about fundamental right and wrong,” and “a reflection of your core moral beliefs and convictions” on 5-point scales labeled *not at all, slightly, moderately, much,* and *very much* (skin rash treatments: α = .80, gun control: α = .81, carbon emissions: α = .86, nuclear power: α = .81, same-sex marriage: α = .86, health care: α = .86, immigration: α = .83).

**Religious conviction.** Religious conviction was a measured with a single face-valid item, specifically, the extent to which participants indicated that their attitudes were “connected to their religious beliefs and convictions,” with the same response options we used for moral conviction. The religious and moral conviction items were interspersed with other items assessing attitude strength (e.g., attitude certainty, importance).

**Political orientation.** Political orientation was measured by asking participants whether they generally think of themselves as liberal, conservative, or uncertain. Participants who indicate whether they are liberal or conservative subsequently branched to an item that asked how strongly they identified as liberal or conservative, with the response options of *slightly, moderately, much, and very much.* Participants who response with “uncertain” branched to an item that asked whether they leaned more toward liberal or conservative, or neither. Leaners were recoded as either *slightly liberal* or *slightly conservative*, and responses to these combined items were used to create a 9-point measure of political orientation, with higher scores reflecting greater conservativism.

## Results

### Skin Rash Treatment

As can be seen in Table S2.1, moral and religious conviction for attitude positions on skin rash treatments were moderate to highly correlated, though still below our golden thread hypothesis threshold of .70. Additionally, as seen in Table S2.2, this relationship was not moderated by political orientation. These results most closely support our distinct constructs hypothesis and do not support our broad culture war hypothesis.

| Table S2.1  *Descriptive Statistics and Bivariate Correlations for Skin Rash Treatment* | | | | | | |
| --- | --- | --- | --- | --- | --- | --- |
|  |  | *M* | *SD* | (1) | (2) | (3) |
| (1) | Political Orientation | -0.46 | 2.42 | - |  |  |
| (2) | Religious Conviction | 1.39 | 0.89 | .21** | - |  |
| (3) | Moral Conviction | 2.07 | 1.16 | .17* | .53** | - |
| *Note.* * *p* < .05. ** *p* < .01. Higher scores on political orientation indicate greater conservatism. | | | | | | |

| Table S2.2  *Hierarchical Regression Model Predicting Skin Rash Treatment Moral Conviction* | | | | |
| --- | --- | --- | --- | --- |
|  |  | Moral Conviction | |  |
|  | Predictor | *B* | *SE* | Δ*R*^2^ |
| Block 1  df = 219 | Political Orientation | 0.03 | 0.03 |  |
|  | Religious Conviction | 0.67** | 0.08 | .28** |
| Block 2  df = 218 | Political Orientation X Religious Conviction | 0.05 | 0.03 | .01 |
| *R*^2^ |  |  |  | .29** |
| *Note*. * *p* < .05. ** *p* < .01. Higher scores on political orientation indicate greater conservatism. | | | | |

### Gun Control

As can be seen in Table S2.3 and in support of our distinct constructs hypothesis, moral and religious conviction for attitude positions on gun control were only moderately correlated. As can be seen in Table S2.4, the relationship between religious conviction and moral conviction was not moderated by political orientation, providing no support for our broad culture war hypothesis.

Table S2.3

*Descriptive Statistics and Bivariate Correlations for Gun Control*

|  |  | *M* | *SD* | (1) | (2) | (3) |
| --- | --- | --- | --- | --- | --- | --- |
| (1) | Political Orientation | -0.50 | 2.23 | - |  |  |
| (2) | Religious Conviction | 1.82 | 1.28 | .11 | - |  |
| (3) | Moral Conviction | 3.32 | 1.15 | -.05 | .31** | - |

*Note.* * *p* < .05. ** *p* < .01. Higher scores on political orientation indicate greater conservatism.

Table S2.4

*Hierarchical Regression Model Predicting Gun Control Moral Conviction*

|  |  | Moral Conviction | |  |
| --- | --- | --- | --- | --- |
|  | Predictor | *B* | *SE* | Δ*R*^2^ |
| Block 1  df = 210 | Political Orientation | -0.04 | 0.03 |  |
|  | Religious Conviction | 0.28** | 0.06 | .10** |
| Block 2  df = 209 | Political Orientation X Religious Conviction | -0.01 | 0.03 | .000 |
| *R*^2^ |  |  |  | .10** |

*Note*. * *p* < .05. ** *p* < .01. Higher scores on political orientation indicate greater conservatism.

### Carbon Emissions

As can be seen in Table S2.5 and in support of our distinct constructs hypothesis, moral and religious conviction for attitude positions on carbon emissions were only moderately correlated. As can be seen in Table S2.6, the relationship between religious conviction and moral conviction was not moderated by political orientation, providing no support for our broad culture war hypothesis.

Table S2.5

*Descriptive Statistics and Bivariate Correlations for Carbon Emissions*

|  |  | *M* | *SD* | (1) | (2) | (3) |
| --- | --- | --- | --- | --- | --- | --- |
| (1) | Political Orientation | 0.04 | 2.37 | - |  |  |
| (2) | Religious Conviction | 1.64 | 1.07 | .06 | - |  |
| (3) | Moral Conviction | 2.77 | 1.26 | -.08 | .42** | - |

*Note.* * *p* < .05. ** *p* < .01. Higher scores on political orientation indicate greater conservatism.

Table S2.6

*Hierarchical Regression Model Predicting Carbon Emissions Moral Conviction*

|  |  | Moral Conviction | |  |
| --- | --- | --- | --- | --- |
|  | Predictor | *B* | *SE* | Δ*R*^2^ |
| Block 1  df = 185 | Political Orientation | -0.06 | 0.04 |  |
|  | Religious Conviction | 0.51** | 0.08 | .19** |
| Block 2  df = 184 | Political Orientation X Religious Conviction | 0.06 | 0.04 | .01 |
| *R*^2^ |  |  |  | .20** |

*Note*. * *p* < .05. ** *p* < .01. Higher scores on political orientation indicate greater conservatism.

### Nuclear Power

As can be seen in Table S2.7 and in support of our distinct constructs hypothesis, moral and religious conviction for attitude positions on nuclear power were only moderately correlated. As can be seen in Table S2.8, the relationship between religious conviction and moral conviction was not moderated by political orientation, providing no support for our broad culture war hypothesis.

Table S2.7

*Descriptive Statistics and Bivariate Correlations for Nuclear Power*

|  |  | *M* | *SD* | (1) | (2) | (3) |
| --- | --- | --- | --- | --- | --- | --- |
| (1) | Political Orientation | 0.19 | 2.34 | - |  |  |
| (2) | Religious Conviction | 1.50 | 1.02 | .06 | - |  |
| (3) | Moral Conviction | 2.48 | 1.23 | -.22** | .44** | - |

*Note.* * *p* < .05. ** *p* < .01. Higher scores on political orientation indicate greater conservatism.

Table S2.8

*Hierarchical Regression Model Predicting Nuclear Power Moral Conviction*

|  |  | Moral Conviction | |  |
| --- | --- | --- | --- | --- |
|  | Predictor | *B* | *SE* | Δ*R*^2^ |
| Block 1  df = 207 | Political Orientation | -0.13** | 0.03 |  |
|  | Religious Conviction | 0.55** | 0.07 | .26** |
| Block 2  df = 206 | Political Orientation X Religious Conviction | 0.05 | 0.03 | .01 |
| *R*^2^ |  |  |  | .27** |

*Note*. * *p* < .05. ** *p* < .01. Higher scores on political orientation indicate greater conservatism.

### Same-Sex Marriage

As can be seen in Table S2.9 and in support of our distinct constructs hypothesis, moral and religious conviction for attitude positions on same-sex marriage were only moderately correlated. However, as can be seen in Table S2.10, the relationship between religious conviction and moral conviction was moderated by political orientation such that the positive relationship between religious conviction and moral conviction was strongest for conservatives, slightly weaker for moderates, and non-significant for liberals (see Figure S2.1 and Table S2.11). These results most closely support the broad culture war hypothesis.

Table S2.9

*Descriptive Statistics and Bivariate Correlations for Same-Sex Marriage*

|  |  | *M* | *SD* | (1) | (2) | (3) |
| --- | --- | --- | --- | --- | --- | --- |
| (1) | Political Orientation | -0.28 | 2.53 | - |  |  |
| (2) | Religious Conviction | 2.53 | 1.73 | .47** | - |  |
| (3) | Moral Conviction | 3.74 | 1.28 | -.01 | .40** | - |

*Note.* * *p* < .05. ** *p* < .01. Higher scores on political orientation indicate greater conservatism.

Table S2.10

*Hierarchical Regression Model Predicting Same-Sex Marriage Moral Conviction*

|  |  | Moral Conviction | |  |
| --- | --- | --- | --- | --- |
|  | Predictor | *B* | *SE* | Δ*R*^2^ |
| Block 1  df = 189 | Political Orientation | -0.13** | 0.04 |  |
|  | Religious Conviction | 0.39** | 0.05 | .21** |
| Block 2  df = 188 | Political Orientation X Religious Conviction | 0.11** | 0.02 | .12** |
| *R*^2^ |  |  |  | .33** |

*Note*. * *p* < .05. ** *p* < .01. Higher scores on political orientation indicate greater conservatism.


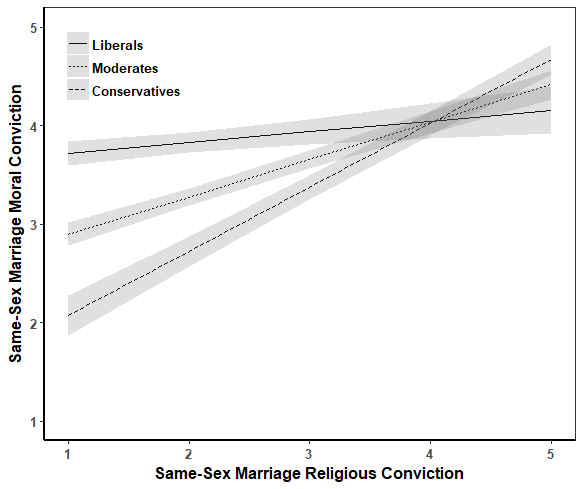


*Figure S2.1.* Simple slopes for the religious conviction by political orientation interaction predicting same-sex marriage moral conviction from Study 2. Political orientation is centered on moderates, 1 *SD* below moderate = liberals, 1 *SD* above moderate = conservatives. Ribbons indicate standard error.

Table S2.11

*Simple Slopes for the Religious Conviction by Political Orientation Interaction Predicting Same-Sex Marriage Moral Conviction*

|  | Religious Conviction Predicting Moral Conviction | | | |
| --- | --- | --- | --- | --- |
| Level of Moderator | *B* | *SE* | *t* | *p* |
| Liberals  (1 *SD* below moderate) | 0.11 | 0.07 | 1.57 | .12 |
| Moderates | 0.38 | 0.05 | 7.60 | <.001 |
| Conservatives  (1 *SD* above moderate) | 0.65 | 0.07 | 9.59 | <.001 |

*Note.* df = 188.

### Health Care

As can be seen in Table S2.12 and in support of our distinct constructs hypothesis, moral and religious conviction for attitude positions on health care were only moderately correlated. As can be seen in Table S2.13, the relationship between religious conviction and moral conviction was not moderated by political orientation, providing no support for our broad culture war hypothesis.

Table S2.12

*Descriptive Statistics and Bivariate Correlations for Health Care*

|  |  | *M* | *SD* | (1) | (2) | (3) |
| --- | --- | --- | --- | --- | --- | --- |
| (1) | Political Orientation | -0.43 | 2.28 | - |  |  |
| (2) | Religious Conviction | 1.60 | 1.10 | .05 | - |  |
| (3) | Moral Conviction | 3.30 | 1.22 | -.24** | .30** | - |

*Note.* * *p* < .05. ** *p* < .01. Higher scores on political orientation indicate greater conservatism.

Table S2.13

*Hierarchical Regression Model Predicting Health Care Moral Conviction*

|  |  | Moral Conviction | |  |
| --- | --- | --- | --- | --- |
|  | Predictor | *B* | *SE* | Δ*R*^2^ |
| Block 1  df = 212 | Political Orientation | -0.14*** | 0.03 |  |
|  | Religious Conviction | 0.35*** | 0.07 | .16*** |
| Block 2  df = 211 | Political Orientation X Religious Conviction | 0.05 | 0.03 | .01 |
| *R*^2^ |  |  |  | .17*** |

*Note*. * *p* < .05. ** *p* < .01. *** *p* < .001. Higher scores on political orientation indicate greater conservatism.

### Immigration

As can be seen in Table S2.14 and in support of our distinct constructs hypothesis, moral and religious conviction for attitude positions on immigration were only weakly to moderately correlated. As can be seen in Table S2.15, the relationship between religious conviction and moral conviction was not moderated by political orientation, providing no support for our broad culture war hypothesis.

Table S2.14

*Descriptive Statistics and Bivariate Correlations for Immigration*

|  |  | *M* | *SD* | (1) | (2) | (3) |
| --- | --- | --- | --- | --- | --- | --- |
| (1) | Political Orientation | -0.17 | 2.37 | - |  |  |
| (2) | Religious Conviction | 1.84 | 1.20 | .07 | - |  |
| (3) | Moral Conviction | 3.33 | 1.18 | .03 | .28** | - |

*Note.* * *p* < .05. ** *p* < .01. Higher scores on political orientation indicate greater conservatism.

Table S2.15

*Hierarchical Regression Model Predicting Immigration Moral Conviction*

|  |  | Moral Conviction | |  |
| --- | --- | --- | --- | --- |
|  | Predictor | *B* | *SE* | Δ*R*^2^ |
| Block 1  df = 205 | Political Orientation | 0.004 | 0.03 |  |
|  | Religious Conviction | 0.27** | 0.07 | .08** |
| Block 2  df = 204 | Political Orientation X Religious Conviction | 0.01 | 0.03 | .001 |
| *R*^2^ |  |  |  | .08** |

*Note*. * *p* < .05. ** *p* < .01. Higher scores on political orientation indicate greater conservatism.

# Study 3: Washburn & Skitka, 2014

## Method

This study was approved by the Institutional Review Board of the Office for the Protection of Research Subjects at the University of Illinois at Chicago. Prior to completing any study measures, participants were presented with an informed consent form with information about the study and consented to participate.

### Participants

Participants were recruited from Amazon’s Mechanical Turk website (Mturk) starting on August 13, 2014. Our sample consisted of 170 participants who completed relevant measures. Participants ranged in age from 19 to 72 (*M* = 35.99, *SD* = 13.58) and were 54% female and 46% male. One percent had less than a high school education, 14% had a high school diploma or GED, 19% had some college but no degree, 15% had a technical/associates degree, 42% had a bachelor’s degree, 8% had a master’s degree, and 1% had a doctoral/professional degree.

### Measures

Participants completed measures of moral and religious conviction (interspersed with other items) about their attitudes on gun control. They also completed a measure of political orientation.

**Moral conviction.** Moral convictions associated with their gun control attitude were measured with a validated two item measure. Participants rated the degree to which their attitudes were “connected to your beliefs about fundamental right and wrong,” and “a reflection of your core moral beliefs and convictions” on 5-point scales labeled *not at all, slightly, moderately, much,* and *very much* (α = .86).

**Religious conviction.** Religious conviction was a measured with a single face-valid item, specifically, the extent to which participants indicated that their attitudes were “connected to their religious beliefs and convictions,” with the same response options we used for moral conviction. The religious and moral conviction items were interspersed with other items assessing attitude strength (e.g., attitude certainty, importance).

**Political orientation.** Political orientation was measured by asking participants whether they generally think of themselves as liberal, conservative, or uncertain. Participants who indicate whether they are liberal or conservative subsequently branched to an item that asked how strongly they identified as liberal or conservative, with the response options of *slightly, moderately, much, and very much.* Participants who response with “uncertain” branched to an item that asked whether they leaned more toward liberal or conservative, or neither. Leaners were recoded as either *slightly liberal* or *slightly conservative*, and responses to these combined items were used to create a 9-point measure of political orientation, with higher scores reflecting greater conservativism.

## Results

As can be seen in Table S3.1 and in support of our distinct constructs hypothesis, moral and religious conviction for attitude positions on gun control were only weakly correlated. As can be seen in Table S3.2, the relationship between religious conviction and moral conviction was not moderated by political orientation, providing no support for our broad culture war hypothesis.

Table S3.1

*Descriptive Statistics and Bivariate Correlations for Gun Control*

|  |  | *M* | *SD* | (1) | (2) | (3) |
| --- | --- | --- | --- | --- | --- | --- |
| (1) | Political Orientation | -0.25 | 2.37 | - |  |  |
| (2) | Religious Conviction | 1.92 | 1.27 | .19* | - |  |
| (3) | Moral Conviction | 3.55 | 1.12 | .06 | .24** | - |

*Note.* * *p* < .05. ** *p* < .01. Higher scores on political orientation indicate greater conservatism.

Table S3.2

*Hierarchical Regression Model Predicting Gun Control Moral Conviction*

|  |  | Moral Conviction | |  |
| --- | --- | --- | --- | --- |
|  | Predictor | *B* | *SE* | Δ*R*^2^ |
| Block 1  df = 167 | Political Orientation | 0.005 | 0.04 |  |
|  | Religious Conviction | 0.21** | 0.07 | .06** |
| Block 2  df = 166 | Political Orientation X Religious Conviction | -0.02 | 0.03 | .003 |
| *R*^2^ |  |  |  | .06* |

*Note*. * *p* < .05. ** *p* < .01. Higher scores on political orientation indicate greater conservatism.

# Study 4: Mallett, Washburn, & Skitka, under review

## Method

This study was approved by the Institutional Review Board of the Office for the Protection of Research Subjects at the University of Illinois at Chicago. Prior to completing any study measures, participants were presented with an informed consent form with information about the study and consented to participate.

### Participants

Participants were recruited from Amazon’s Mechanical Turk (Mturk) starting on November 17, 2016. Our sample consisted of 346 participants who completed relevant measures. Participants ranged in age from 18 to 81 (*M* = 37.18, *SD* = 12.73) and were 58% male and 42% female. One percent had less than high school diploma, 13% had a high school diploma or GED, 25% had some college but no degree, 11% had a technical/associates degree, 36% had a bachelor’s degree, 13% had a master’s degree, and 1% had a doctoral/professional degree.

### Measures

Participants completed measures of moral and religious conviction (interspersed with other items) about their attitudes on the war on women, the war on Christmas, or workplace professionalism. They also completed a measure of political orientation.

**Moral conviction.** Moral convictions associated with their attitudes were measured with a validated two item measure. Participants rated the degree to which their attitudes were “connected to your beliefs about fundamental right and wrong,” and “a reflection of your core moral beliefs and convictions” on 5-point scales labeled *not at all, slightly, moderately, much,* and *very much* (war on women: α = .84, war on Christmas: α = .92, workplace professionalism: α = .85).

**Religious conviction.** Religious conviction was a measured with a single face-valid item, specifically, the extent to which participants indicated that their attitudes were “connected to their religious beliefs and convictions,” with the same response options we used for moral conviction. The religious and moral conviction items were interspersed with other items assessing attitude strength (e.g., attitude certainty, importance).

**Religiosity.** We used three items from the Santa Clara Strength of Religiosity scale to measure religiosity. Participants were asked how much each of the following states described them: “My religious faith is extremely important to me,” “My religious faith impacts many of my decisions,” and “I look to faith for meaning and purpose in my life,” with the response options of *not at all, slightly, moderately, much,* and *very much* (α = .98).

**Political orientation.** Political orientation was measured by asking participants whether they generally think of themselves as liberal, conservative, or uncertain. Participants who indicate whether they are liberal or conservative subsequently branched to an item that asked how strongly they identified as liberal or conservative, with the response options of *slightly, moderately, much, and very much.* Participants who response with “uncertain” branched to an item that asked whether they leaned more toward liberal or conservative, or neither. Leaners were recoded as either *slightly liberal* or *slightly conservative*, and responses to these combined items were used to create a 9-point measure of political orientation, with higher scores reflecting greater conservativism.

## Results

### War on Christmas

As can be seen in Table S4.1, moral and religious conviction for attitude positions on the war on Christmas were moderate to highly correlated, though still below our golden thread hypothesis threshold of .70. Additionally, as seen in Table S4.2, this relationship was not moderated by political orientation, religiosity, or a combination of the two. These results most closely support our distinct constructs hypothesis and do not support our broad culture war or secularization hypotheses.

Table S4.1

*Descriptive Statistics and Bivariate Correlations for War on Christmas*

|  |  | *M* | *SD* | (1) | (2) | (3) | (4) |
| --- | --- | --- | --- | --- | --- | --- | --- |
| (1) | Political Orientation | 0.09 | 2.70 | - |  |  |  |
| (2) | Religiosity | 3.93 | 2.41 | .49** | - |  |  |
| (3) | Religious Conviction | 2.30 | 1.45 | .51** | .47** | - |  |
| (4) | Moral Conviction | 2.65 | 1.37 | .38** | .32** | .60** | - |

*Note.* * *p* < .05. ** *p* < .01. Higher scores on political orientation indicate greater conservatism.

Table S4.2

*Hierarchical Regression Model Predicting War on Christmas Moral Conviction*

|  |  | Moral Conviction | |  |
| --- | --- | --- | --- | --- |
|  | Predictor | *B* | *SE* | Δ*R*^2^ |
| Block 1  df = 111 | Political Orientation | 0.05 | 0.05 |  |
|  | Religiosity | 0.01 | 0.05 |  |
|  | Religious Conviction | 0.52** | 0.09 | .37** |
| Block 2  df = 108 | Religiosity X Religious Conviction | 0.07 | 0.04 |  |
|  | Political Orientation X Religiosity | -0.02 | 0.02 |  |
|  | Political Orientation X Religious Conviction | 0.06 | 0.03 | .07** |
| Block 3  df = 107 | Religiosity X Religious Conviction X Political Orientation | 0.002 | 0.01 | .000 |
| *R*^2^ |  |  |  | .44** |

*Note*. * *p* < .05. ** *p* < .01. Higher scores on political orientation indicate greater conservatism.

### War on Women

As can be seen in Table S4.3 and in support of our distinct constructs hypothesis, moral and religious conviction for attitude positions on the war on women were only moderately correlated. As can be seen in Table S4.4, the relationship between religious conviction and moral conviction was moderated by political orientation such that the positive relationship between religious conviction and moral conviction was strongest for conservatives, slightly weaker for moderates, and non-significant for liberals (see Figure S4.1 and Table S4.5). These results most closely support the broad culture war hypothesis.

Table S4.3

*Descriptive Statistics and Bivariate Correlations for War on Women*

|  |  | *M* | *SD* | (1) | (2) | (3) | (4) |
| --- | --- | --- | --- | --- | --- | --- | --- |
| (1) | Political Orientation | -0.22 | 2.52 | - |  |  |  |
| (2) | Religiosity | 3.79 | 2.43 | .41** | - |  |  |
| (3) | Religious Conviction | 1.93 | 1.30 | .11 | .55** | - |  |
| (4) | Moral Conviction | 3.18 | 1.25 | -.28** | -.02 | .30** | - |

*Note.* * *p* < .05. ** *p* < .01. Higher scores on political orientation indicate greater conservatism.

Table S4.4

*Hierarchical Regression Model Predicting War on Women Moral Conviction*

|  |  | Moral Conviction | |  |
| --- | --- | --- | --- | --- |
|  | Predictor | *B* | *SE* | Δ*R*^2^ |
| Block 1  df = 112 | Political Orientation | -0.13** | 0.05 |  |
|  | Religiosity | -0.07 | 0.06 |  |
|  | Religious Conviction | 0.40** | 0.10 | .20** |
| Block 2  df = 109 | Religiosity X Religious Conviction | 0.06 | 0.05 |  |
|  | Political Orientation X Religiosity | -0.05* | 0.02 |  |
|  | Political Orientation X Religious Conviction | 0.09* | 0.04 | .07* |
| Block 3  df = 108 | Religiosity X Religious Conviction X Political Orientation | -0.004 | 0.02 | .000 |
| *R*^2^ |  |  |  | .27** |

*Note*. * *p* < .05. ** *p* < .01. Higher scores on political orientation indicate greater conservatism.


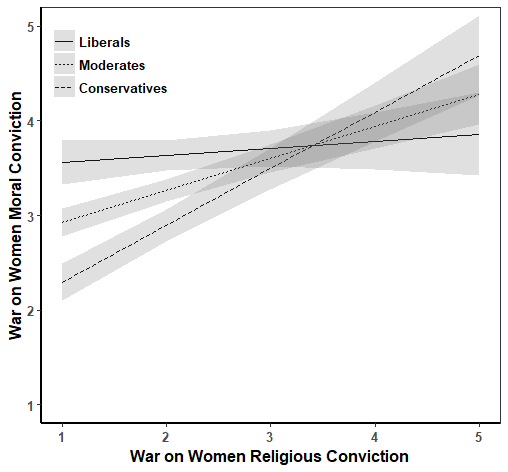


*Figure S4.1.* Simple slopes for the religious conviction by political orientation interaction predicting war on women moral conviction from Study 4. Political orientation is centered on moderates, 1 *SD* below moderate = liberals, 1 *SD* above moderate = conservatives. Ribbons indicate standard error.

Table S4.5

*Simple Slopes for the Religious Conviction by Political Orientation Interaction Predicting War on Women Moral Conviction*

|  | Religious Conviction Predicting Moral Conviction | | | |
| --- | --- | --- | --- | --- |
| Level of Moderator | *B* | *SE* | *t* | *p* |
| Liberals  (1 *SD* below moderate) | 0.08 | 0.15 | 0.52 | .63 |
| Moderates | 0.34 | 0.10 | 3.44 | .001 |
| Conservatives  (1 *SD* above moderate) | 0.60 | 0.12 | 4.91 | <.001 |

*Note.* df = 110.

Though not relevant for our hypotheses, there was also an interaction between political orientation and religiosity predicting war on women moral conviction (see Table S4.4). There was a negative relationship between religiosity and moral conviction for conservatives and no relationship between religiosity and moral conviction for moderates or liberals (see Figure S4.2 and Table S4.6).


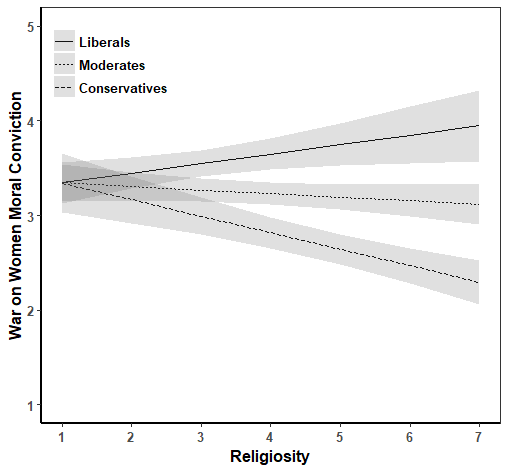


*Figure S4.2.* Simple slopes for the religiosity by political orientation interaction predicting war on women moral conviction from Study 4. Political orientation is centered on moderates, 1 *SD* below moderate = liberals, 1 *SD* above moderate = conservatives. Ribbons indicate standard error.

Table S4.6

*Simple Slopes for the Religiosity by Political Orientation Interaction Predicting War on Women Moral Conviction*

|  | Religiosity Predicting Moral Conviction | | | |
| --- | --- | --- | --- | --- |
| Level of Moderator | *B* | *SE* | *t* | *p* |
| Liberals  (1 *SD* below moderate) | 0.10 | 0.09 | 1.17 | .25 |
| Moderates | -0.04 | 0.06 | -0.65 | .52 |
| Conservatives  (1 *SD* above moderate) | -0.18 | 0.08 | -2.34 | .02 |

*Note.* df = 110.

### Workplace Professionalism

As can be seen in Table S4.7, moral and religious conviction for attitude positions on the workplace professionalism were moderate to highly correlated, though still below our golden thread hypothesis threshold of .70. Additionally, as seen in Table S4.8, this relationship was not moderated by political orientation, religiosity, or a combination of the two. These results most closely support our distinct constructs hypothesis and do not support our broad culture war or secularization hypotheses.

Table S4.7

*Descriptive Statistics and Bivariate Correlations for Workplace Professionalism*

|  |  | *M* | *SD* | (1) | (2) | (3) | (4) |
| --- | --- | --- | --- | --- | --- | --- | --- |
| (1) | Political Orientation | -0.48 | 2.49 | - |  |  |  |
| (2) | Religiosity | 3.33 | 2.41 | .43** | - |  |  |
| (3) | Religious Conviction | 1.86 | 1.22 | .29** | .61** | - |  |
| (4) | Moral Conviction | 2.97 | 1.20 | .12 | .32** | .48** | - |

*Note.* * *p* < .05. ** *p* < .01. Higher scores on political orientation indicate greater conservatism.

Table S4.8

*Final Hierarchical Regression Model Predicting Workplace Professionalism Moral Conviction*

|  |  | Moral Conviction | |  |
| --- | --- | --- | --- | --- |
|  | Predictor | *B* | *SE* | Δ*R*^2^ |
| Block 1  df = 111 | Political Orientation | -0.02 | 0.04 |  |
|  | Religiosity | 0.03 | 0.06 |  |
|  | Religious Conviction | 0.45** | 0.10 | .24** |
| Block 2  df = 108 | Religiosity X Religious Conviction | 0.07 | 0.05 |  |
|  | Political Orientation X Religiosity | 0.04 | 0.02 |  |
|  | Political Orientation X Religious Conviction | -0.08 | 0.05 | .02 |
| Block 3  df = 107 | Religiosity X Religious Conviction X Political Orientation | 0.02 | 0.02 | .01 |
| *R*^2^ |  |  |  | .27** |

*Note*. * *p* < .05. ** *p* < .01. Higher scores on political orientation indicate greater conservatism.

# Study 5: Mallett, Washburn, & Skitka, under review

## Method

This study was approved by the Institutional Review Board of the Office for the Protection of Research Subjects at the University of Illinois at Chicago and the Human Subjects Institutional Review Board of the Office of Research Integrity at James Madison University. Prior to completing any study measures, participants were presented with an informed consent form with information about the study and consented to participate.

### Participants

Participants were recruited from the University of Illinois at Chicago and James Madison University starting on March 22, 2017. Our sample consisted of 529 participants who completed relevant measures. Participants ranged in age from 18 to 73 (*M* = 21.37, *SD* = 6.90) and were 35% male and 64% female. Nineteen percent had a high school diploma or GED, 60% had some college but no degree, 5% had a technical/associates degree, 9% had a bachelor’s degree, 6% had a master’s degree, and 1% had a doctoral/professional degree.

### Measures

Participants completed measures of moral and religious conviction (interspersed with other items) about their attitudes on conservative bias in universities, liberal bias in university, or workplace professionalism. They also completed a measure of political orientation.

**Moral conviction.** Moral convictions associated with their attitudes were measured with a validated two item measure. Participants rated the degree to which their attitudes were “connected to your beliefs about fundamental right and wrong,” and “a reflection of your core moral beliefs and convictions” on 5-point scales labeled *not at all, slightly, moderately, much,* and *very much* (conservative bias: α = .82, liberal bias: α = .76, workplace professionalism: α = .79).

**Religious conviction.** Religious conviction was a measured with a single face-valid item, specifically, the extent to which participants indicated that their attitudes were “connected to their religious beliefs and convictions,” with the same response options we used for moral conviction. The religious and moral conviction items were interspersed with other items assessing attitude strength (e.g., attitude certainty, importance).

**Religiosity.** We used three items from the Santa Clara Strength of Religiosity scale to measure religiosity. Participants were asked how much each of the following states described them: “My religious faith is extremely important to me,” “My religious faith impacts many of my decisions,” and “I look to faith for meaning and purpose in my life,” with the response options of *not at all, slightly, moderately, much,* and *very much* (α = .97).

**Political orientation.** Political orientation was measured by asking participants whether they generally think of themselves as liberal, conservative, or uncertain. Participants who indicate whether they are liberal or conservative subsequently branched to an item that asked how strongly they identified as liberal or conservative, with the response options of *slightly, moderately, much, and very much.* Participants who response with “uncertain” branched to an item that asked whether they leaned more toward liberal or conservative, or neither. Leaners were recoded as either *slightly liberal* or *slightly conservative*, and responses to these combined items were used to create a 9-point measure of political orientation, with higher scores reflecting greater conservativism.

## Results

### Conservative Bias in Universities

As can be seen in Table S5.1, moral and religious conviction for attitude positions on the conservative bias in universities were moderately correlated, though still below our golden thread hypothesis threshold of .70. Additionally, as seen in Table S5.2, this relationship was not moderated by political orientation, religiosity, or a combination of the two. These results most closely support our distinct constructs hypothesis and do not support our broad culture war or secularization hypotheses.

Table S5.1

*Descriptive Statistics and Bivariate Correlations for Conservative Bias at Universities*

|  |  | *M* | *SD* | (1) | (2) | (3) | (4) |
| --- | --- | --- | --- | --- | --- | --- | --- |
| (1) | Political Orientation | -0.62 | 2.09 | - |  |  |  |
| (2) | Religiosity | 4.58 | 2.45 | .41** | - |  |  |
| (3) | Religious Conviction | 1.93 | 1.11 | .29** | .46** | - |  |
| (4) | Moral Conviction | 2.75 | 1.12 | -.01 | .04 | .44** | - |

*Note.* * *p* < .05. ** *p* < .01. Higher scores on political orientation indicate greater conservatism.

Table S5.2

*Hierarchical Regression Model Predicting Conservative Bias at Universities Moral Conviction*

|  |  | Moral Conviction | |  |
| --- | --- | --- | --- | --- |
|  | Predictor | *B* | *SE* | Δ*R*^2^ |
| Block 1  df = 170 | Political Orientation | -0.06 | 0.04 |  |
|  | Religiosity | -0.08* | 0.04 |  |
|  | Religious Conviction | 0.55** | 0.08 | .23** |
| Block 2  df = 167 | Religiosity X Religious Conviction | 0.02 | 0.03 |  |
|  | Political Orientation X Religiosity | 0.03 | 0.02 |  |
|  | Political Orientation X Religious Conviction | 0.02 | 0.04 | .03 |
| Block 3  df = 166 | Religiosity X Religious Conviction X Political Orientation | -0.01 | 0.02 | .000 |
| *R*^2^ |  |  |  | .26** |

*Note*. * *p* < .05. ** *p* < .01. Higher scores on political orientation indicate greater conservatism.

### Liberal Bias in Universities

As can be seen in Table S5.3, moral and religious conviction for attitude positions on the liberal bias in universities were moderate to highly correlated, though still below our golden thread hypothesis threshold of .70. Additionally, as seen in Table S5.4, this relationship was not moderated by political orientation, religiosity, or a combination of the two. These results most closely support our distinct constructs hypothesis and do not support our broad culture war or secularization hypotheses.

Table S5.3

*Descriptive Statistics and Bivariate Correlations for Liberal Bias at Universities*

|  |  | *M* | *SD* | (1) | (2) | (3) | (4) |
| --- | --- | --- | --- | --- | --- | --- | --- |
| (1) | Political Orientation | -0.06 | 2.05 | - |  |  |  |
| (2) | Religiosity | 4.82 | 2.65 | .39** | - |  |  |
| (3) | Religious Conviction | 2.01 | 1.22 | .30** | .55** | - |  |
| (4) | Moral Conviction | 2.83 | 1.11 | .10 | .08 | .52** | - |

*Note.* * *p* < .05. ** *p* < .01. Higher scores on political orientation indicate greater conservatism.

Table S5.4

*Hierarchical Regression Model Predicting Liberal Bias at Universities Moral Conviction*

|  |  | Moral Conviction | |  |
| --- | --- | --- | --- | --- |
|  | Predictor | *B* | *SE* | Δ*R*^2^ |
| Block 1  df = 172 | Political Orientation | 0.01 | 0.04 |  |
|  | Religiosity | -0.12** | 0.03 |  |
|  | Religious Conviction | 0.61** | 0.07 | .33** |
| Block 2  df = 169 | Religiosity X Religious Conviction | 0.06 | 0.03 |  |
|  | Political Orientation X Religiosity | -0.01 | 0.02 |  |
|  | Political Orientation X Religious Conviction | 0.05 | 0.04 | .03* |
| Block 3  df = 168 | Religiosity X Religious Conviction X Political Orientation | -0.00001 | 0.01 | .000 |
| *R*^2^ |  |  |  | .36** |

*Note*. * *p* < .05. ** *p* < .01. Higher scores on political orientation indicate greater conservatism.

### Workplace Professionalism

As can be seen in Table S5.5, moral and religious conviction for attitude positions on workplace professionalism were moderately correlated, though still below our golden thread hypothesis threshold of .70. Additionally, as seen in Table S5.6, this relationship was not moderated by political orientation, religiosity, or a combination of the two. These results most closely support our distinct constructs hypothesis and do not support our broad culture war or secularization hypotheses.

Table S5.5

*Descriptive Statistics and Bivariate Correlations for Workplace Professionalism*

|  |  | *M* | *SD* | (1) | (2) | (3) | (4) |
| --- | --- | --- | --- | --- | --- | --- | --- |
| (1) | Political Orientation | -0.32 | 2.29 | - |  |  |  |
| (2) | Religiosity | 4.47 | 2.49 | .48** | - |  |  |
| (3) | Religious Conviction | 1.91 | 1.18 | .34** | .55** | - |  |
| (4) | Moral Conviction | 3.01 | 1.02 | .31** | .30** | .43** | - |

*Note.* * *p* < .05. ** *p* < .01. Higher scores on political orientation indicate greater conservatism.

Table S5.6

*Hierarchical Regression Model Predicting Workplace Professionalism Moral Conviction*

|  |  | Moral Conviction | |  |
| --- | --- | --- | --- | --- |
|  | Predictor | *B* | *SE* | Δ*R*^2^ |
| Block 1  df = 175 | Political Orientation | 0.08* | 0.03 |  |
|  | Religiosity | 0.01 | 0.04 |  |
|  | Religious Conviction | 0.31** | 0.07 | .22** |
| Block 2  df = 172 | Religiosity X Religious Conviction | 0.02 | 0.03 |  |
|  | Political Orientation X Religiosity | 0.001 | 0.02 |  |
|  | Political Orientation X Religious Conviction | -0.01 | 0.03 | .003 |
| Block 3  df = 171 | Religiosity X Religious Conviction X Political Orientation | 0.02 | 0.02 | .000 |
| *R*^2^ |  |  |  | .22** |

*Note*. * *p* < .05. ** *p* < .01. Higher scores on political orientation indicate greater conservatism.

# Study 6: Mueller, 2016

## Method

Study 6 was a secondary analysis of data originally collected to test whether moral and religious conviction differentially impact people’s willingness to take the perspective of attitudinally dissimilar others (Mueller, 2016). Relevant to the current paper, the data include measures of moral and religious conviction for the issue of legalized abortion, as well as participants’ political orientation. Thus, these data allow us to test the golden thread, culture war (broad form), and distinct constructs hypotheses. This study was approved by the Institutional Review Board of the Office for the Protection of Research Subjects at the University of Illinois at Chicago. Prior to completing any study measures, participants were presented with an informed consent form with information about the study and consented to participate.

### Participants

One hundred thirty-five participants were recruited from the Introductory Psychology subject pool at the University of Illinois at Chicago spanning from February 22, 2016 to February 24, 2017. Participants had to be at least 18 years old to participate (*M* = 18.85, *SD* = 0.99). The sample was 68.7% female, ethnically diverse (36.6% Latino, 27.6% Asian or Pacific Islander, 20.9% White, 11.2% African American, and 3.7% Other).

### Measures

For the purposes of this paper, we focused on three measures: Participants’ reported strength of moral and religious conviction for legalized abortion, as well as their political orientation. More detail is provided below.

**Moral conviction.** Moral convictions for the issue of legalized abortion were measured with a validated four-item measure (Skitka & Morgan, 2014). Participants rated the extent to which their feelings about legalized abortion were “a reflection of your core moral beliefs and convictions,” “a moral stance,” “based on a moral principle,” and “connected to your beliefs about fundamental right and wrong” on 5-point scales labeled *not at all*, *slightly*, *moderately*, *much*, and *very much* (α = .76).

**Religious conviction.** Religious conviction was measured with two face-valid items. Participants rated the extent to which their feelings about legalized abortion were “based on a religious principle,” and “a reflection of your religious beliefs” on 5-point scales labeled *not at all*, *slightly*, *moderately*, *much*, and *very much* (*r* = .84).

**Political orientation.** Participants’ political orientation was assessed with the question, “Are your political beliefs generally liberal or conservative?” Answer choices included *liberal*, *neutral/neither*, and *conservative*. For participants who initially selected *liberal* or *conservative*, they were then asked, “To what extent are your political beliefs liberal (conservative),” followed by the answer choices *slightly*, *moderately*, *much*, and *very much*. In contrast, participants who initially selected *neutral/neither* were asked, “If you had to say which way you lean, would you say you are more conservative or more liberal?” Answer choices included *lean toward liberal*, *lean toward conservative*, and *neutral/neither*. We aggregated these measures to create a single, bipolar measure of political orientation: -4 (*very much liberal*), -3 (*much liberal*), -2 (*moderately liberal*), -1 (*slightly liberal/lean liberal*), 0 (*neutral/neither*), 1 (*slightly conservative/lean conservative*), 2 (*moderately conservative*), 3 (*much conservative*), and 4 (*very much conservative*).

## Results

As shown in Tables S6.1 and S6.2, although moral and religious convictions were weakly correlated. Therefore, we can conclude that the constructs are more distinct than overlapping. Moreover, we found little evidence for the broad form culture wars hypothesis. Political orientation did not significantly influence the relationship between religious and moral conviction for the issue of legalized abortion.

Table S6.1

*Descriptive Statistics and Correlations of Key Variables in Study 6*

| Variable | *M* | *SD* | 1 | 2 | 3 |
| --- | --- | --- | --- | --- | --- |
| 1. PO | -1.05 | 1.55 | - |  |  |
| 1. RC | 2.27 | 1.35 | .09 | - |  |
| 1. MC | 3.61 | 0.90 | .01 | .21* | - |

**p* < .05. *Note*. MC = moral conviction for legalized abortion; RC = religious conviction for legalized abortion; PO = political orientation.

Table S6.2

*Hierarchical Regression Model Predicting Legalized Abortion Moral Conviction*

|  | Predictor | Moral Conviction | |  |
| --- | --- | --- | --- | --- |
|  |  | *B* | *SE* | Δ *R^2^* |
| Block 1  df = 132 | PO | -0.01 | 0.05 |  |
|  | Religious Conviction | 0.14* | 0.06 | .04^†^ |
| Block 2  df = 131 | PO X Religious Conviction | 0.06^†^ | 0.03 | .02^†^ |
| *R^2^* |  |  |  | .06** |

† *p* < .10, * *p* < .05, ** *p* < .01

# Study 7: Skitka, Bauman, & Lytle, 2009

## Method

Study 7 was a secondary analysis of data originally collected to test whether moral and religious conviction differentially impact people’s compliance with and reactions to the U.S. Supreme Court before and after it ruled on a case that challenged states’ rights to legalize physician-assisted suicide (PAS; Skitka et al., 2009). Relevant to the current paper, the data include measures of moral and religious conviction for the issue of PAS, political orientation, and religiosity. Thus, these data allow us to test all four hypotheses. This study was approved by the Institutional Review Board of the Office for the Protection of Research Subjects at the University of Illinois at Chicago. Prior to completing any study measures, participants were presented with an informed consent form with information about the study and consented to participate.

### Participants

Eight hundred fifty-one participants were recruited from a panel of respondents maintained by Knowledge Networks (KN). KN recruits panel members using random-digit-dialing telephone methods, and the panel represents the demographic breakdown of the U.S. Census (see http://www.knowledgenetworks.com/ganp/ for more information). The sample size of the current study exceeds the one reported in Skitka et al. (2009) because we only analyzed responses from the first wave of the original study; we made this decision because all key variables for the current investigation were collected during the first, but not second, wave of the original study. Participants had to be at least 18 years old to participate (*M* = 45.33, *SD* = 16.25). The sample was 51.0% female, ethnically representative of the U.S. population (74.4% White, Non-Hispanic; 11.4% Hispanic; 7.3% Black, Non-Hispanic; 3.6% 2+ Races, Non-Hispanic; and 3.3% Other, Non-Hispanic).

### Measures

For the purposes of this paper, we focused on four measures: Participants’ reported strength of moral and religious conviction for PAS, their political orientation, and their religiosity. More detail is provided below.

**Moral conviction.** Moral convictions for the issue of PAS were measured with a two-item measure. Participants rated the extent to which their feelings about whether PAS should be allowed “reflect your core moral values and convictions,” and “deeply connected to your beliefs about ‘right’ and ‘wrong’” on 5-point scales labeled *not at all*, *slightly*, *moderately*, *much*, and *very much* (*r* = .83). Higher scores on this variable refer to greater moral conviction.

**Religious conviction.** Religious conviction was measured with a single face-valid item. Participants rated the extent to which, “My attitude about PAS is closely connected to my religious beliefs” on a 7-point scale labeled *very much disagree*, *moderately disagree*, *slightly disagree*, *neutral or uncertain*, *slightly agree*, *moderately agree*, and *very much agree*. Higher scores on this variable refer to greater religious conviction.

**Political orientation.** Participants’ political orientation was assessed with the question, “In general, do you think of yourself as…” followed by the answer choices *extremely liberal*, *liberal*, *slightly liberal*, *moderate/middle of the road*, *slightly conservative*, *conservative*, and *extremely conservative.* Higher scores on this variable reflect greater conservatism.

**Religiosity.** Religiosity was assessed with a short form of the Santa Clara Strength of Religious Faith Questionnaire (Plante & Boccaccini, 1997). Participants reported their level of agreement or disagreement to the following three items: “My religious faith is extremely important to me,” “My religious faith impacts many of my decisions,” and “I look to faith for meaning and purpose in my life.” Participants responded on 7-point scales, with the point labels of *very much disagree*, *moderately disagree*, *slightly disagree*, *neutral or uncertain*, *slightly agree*, *moderately agree*, and *very much agree* (α = 0.95). Higher scores on this variable indicate greater religiosity.

## Results

Moral and religious conviction were moderately correlated in Study 7, a finding consistent with the distinct constructs hypothesis (see Table S7.1). However, the relationship between moral and religious conviction was qualified by political orientation and religiosity (see Table S7.2). As illustrated in Figure S7.1, religious conviction more strongly predicted moral conviction at high (vs. low) levels of religiosity: a pattern that was generally apparent at all levels of political orientation, not just among conservatives. We therefore interpret these findings as most consistent with the secularization hypothesis: religious conviction most strongly predicted moral conviction among the religious.

Table S7.1

*Descriptive Statistics and Correlations of Key Variables in Study 7: Moral and Religious Conviction For Physician-Assisted Suicide (PAS)*

| Variable | Mean | SD | 1 | 2 | 3 | 4 |
| --- | --- | --- | --- | --- | --- | --- |
| 1. PO | 0.22 | 1.39 | - |  |  |  |
| 1. Religiosity | 1.02 | 1.94 | .28** | - |  |  |
| 1. RC | 0.18 | 2.27 | .31** | .75** | - |  |
| 1. MC | 3.45 | 1.30 | .18** | .24** | .30** | - |

***p* < .01. *Note*. MC = moral conviction for PAS. RC = religious conviction for PAS. PO = political identification, which served as a proxy for political orientation; higher numbers refer to more conservative PO.

Table S7.2

Hierarchical regression model predicting physician-assisted suicide (PAS) moral conviction.

|  | Predictor | Moral Conviction | |  |
| --- | --- | --- | --- | --- |
|  |  | *B* | *SE* | Δ *R^2^* |
| Block 1  df = 847 | Political Orientation | 0.09** | 0.03 |  |
|  | Religiosity | 0.02 | 0.03 |  |
|  | Religious Conviction | 0.14** | 0.03 | .10** |
| Block 2  df = 844 | Religiosity X Religious Conviction | 0.08** | 0.01 |  |
|  | Political Orientation X Religiosity | 0.01 | 0.03 |  |
|  | Political Orientation X Religious Conviction | 0.02 | 0.02 | .06** |
| Block 3  df = 843 | Religiosity X Religious Conviction X Political Orientation | 0.02* | 0.01 | <.01* |
| *R^2^* |  |  |  | .16** |

* *p* < .05, ** *p* < .01


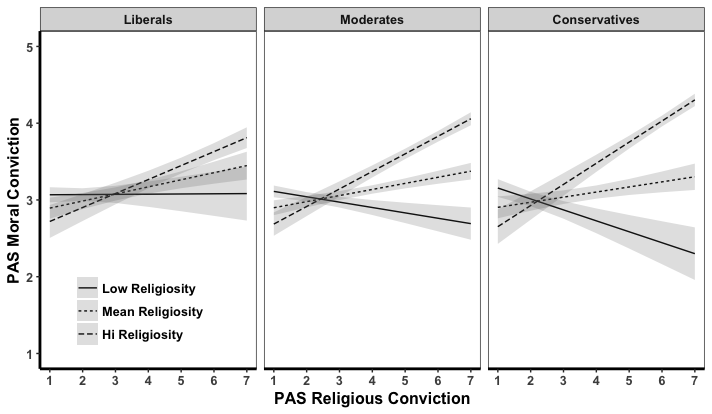


*Figure S7.1.* Simple slopes for the physician-assisted suicide (PAS) religious conviction by religiosity by political orientation interaction predicting PAS moral conviction. Political orientation is centered on moderates, 1 *SD* below moderate = liberal, 1 *SD* above moderate = conservative. Ribbons indicate standard error.

Table S7.3

*Simple Interactions and Slopes for the Physician-Assisted Suicide (PAS) Religious Conviction by Religiosity by Political Orientation Interaction Predicting PAS Moral Conviction*

|  |  | Levels of Political Orientation Moderator | | | | | | | | | | | | | |
| --- | --- | --- | --- | --- | --- | --- | --- | --- | --- | --- | --- | --- | --- | --- | --- |
|  |  | Liberals | | | |  | Moderates | | | |  | Conservatives | | | |
| Simple Interaction |  | *B* | *SE* | *t* | *p* |  | *B* | *SE* | *t* | *p* |  | *B* | *SE* | *t* | *p* |
| Religious Conviction X Religiosity |  | 0.05 | 0.02 | 2.53 | 0.01 |  | 0.08 | 0.01 | 6.54 | <.001 |  | 0.11 | 0.02 | 6.25 | <.001 |
|  |  | Religious Conviction predicting  Moral Conviction | | | |  | Religious Conviction predicting  Moral Conviction | | | |  | Religious Conviction predicting  Moral Conviction | | | |
| Levels of Religiosity Moderator |  | *B* | *SE* | *t* | *p* |  | *B* | *SE* | *t* | *p* |  | *B* | *SE* | *t* | *p* |
| Low Religiosity  (1 *SD* below mean) |  | 0.00 | 0.07 | 0.03 | .97 |  | -0.07 | 0.04 | -1.69 | 0.09 |  | -0.14 | 0.07 | -2.16 | .03 |
| Mean Religiosity |  | 0.09 | 0.04 | 2.07 | 0.04 |  | 0.08 | 0.03 | 2.75 | <.01 |  | 0.07 | 0.04 | 1.51 | 0.13 |
| High Religiosity  (1 *SD* above mean) |  | 0.18 | 0.05 | 3.96 | <.001 |  | 0.23 | 0.03 | 7.22 | <.001 |  | 0.28 | 0.04 | 6.56 | <.001 |

*Note.* df = 986, 1 *SD* below midpoint (Independent/moderate) = Democrat/liberal, 1 *SD* above midpoint (Independent/moderate) = Republican/conservative

# Study 8: Brandt, Wisneski, & Skitka, 2015

## Method

### Study 8 was a secondary analysis of data originally collected to test the affective and cognitive antecedents and consequences of moral conviction in the context of the 2012 U.S. Presidential Election (Brandt et al., 2015). Relevant to the current paper, the data include measures of moral and religious conviction about the participants’ preferred and non-preferred candidates, political orientation, and religiosity. Thus, these data allow us to test all four hypotheses. This dataset was collected by that paper’s first author while at Tilburg University in Tilburg, Netherlands. At the time of data collection (September, 2012), Tilburg University did not have a formal ethical/institutional review board and ethical review of academic research was not required by Dutch law. Participants were presented with information about the risks and benefits of the study, a confidentiality assurance, and contact information for the principal investigator prior to completing any study measures.

### Participants

Participants were recruited from Amazon’s Mechanical Turk website (Mturk) starting on September 12, 2012. Our sample consisted of 1884 participants who were eligible to vote in the election (U.S. citizen, 18 years or older, and registered to vote), who completed relevant measures. Participants ranged in age from 18 to 83 (*M* = 33.33, *SD* = 12.34), 1% had less than a high school education, 12% had a high school diploma or equivalent, 31% had some college but no degree, 11% had a technical/associates degree, 33% had a bachelor’s degree, 10% had a master’s degree, 3% had a doctoral/professional degree, 46% were male, and 46% were female. Although Brandt et al. (2015) collected data longitudinally, analysis here was restricted to the pre-election sample.

### Measures

Participants completed measures of moral and religious conviction (interspersed with other items) about both their preferred major party candidate in the 2012 election, as well as their non-preferred major party candidate. They also completed a measure of political orientation and religiosity.

**Moral conviction.** Moral convictions associated with candidate preferences were measured with a validated three item measure (see Skitka & Morgan, 2014 for more information about validation). Participants rated the degree to which their feelings about their preferred and non-preferred candidate were “connected to your beliefs about fundamental right and wrong,” “a reflection of your core moral beliefs and convictions,” and “based on moral principle” on 5-point scales labeled *not at all, slightly, moderately, much,* and *very much* (Preferred Candidate α = .91, Non-Preferred Candidate α = .94).

**Religious conviction.** Religious conviction was a measured with a single face-valid item, specifically, the extent to which participants indicated that their feelings about about their preferred and non-preferred candidate were “a reflection of your religious beliefs,” with the same response options we used for moral conviction. The religious and moral conviction items were interspersed with other items assessing attitude strength (e.g., attitude certainty, importance).

**Religiosity.** We used three items from the Santa Clara Strength of Religiosity scale to measure religiosity. Participants were asked how much each of the following states described them: “My religious faith is extremely important to me,” “My religious faith impacts many of my decisions,” and “I look to faith for meaning and purpose in my life,” with the response options of *not at all, slightly, moderately, much,* and *very much* (α = .96).

**Political orientation.** Political orientation was measured by asking participants whether they generally think of themselves as liberal, conservative, or something else. Participants who indicate whether they are liberal or conservative subsequently branched to an item that asked how strongly they identified as liberal or conservative, with the response options of *slightly strong, moderately strong,* and *very strong.* Participants who response with “something else” branched to an item that asked whether they leaned more toward liberal or conservative, or neither. Leaners were recoded as either *slightly liberal* or *slightly conservative*, and responses to these combined items were used to create a 7-point measure of political orientation, with higher scores reflecting greater conservativism.

## Results

### Preferred Candidate in 2012 Election

As can be seen in Table S8.1 and in support of our distinct constructs hypothesis, moral and religious conviction for preferred candidate in 2012 election were only moderately correlated. As can be seen in Table S8.2, the relationship between religious conviction and moral conviction was moderated by political orientation such that the positive relationship between religious conviction and moral conviction was strongest for conservatives, slightly weaker for moderates, and weakest for liberals (see Figure S8.1 and Table S8.3). These results most closely support the broad culture war hypothesis.

Table S8.1

*Descriptive Statistics and Bi-Variate Correlations for Preferred Candidate in the 2012 Presidential Election*

|  |  | *M* | *SD* | (1) | (2) | (3) | (4) |
| --- | --- | --- | --- | --- | --- | --- | --- |
| (1) | Political Orientation | -0.67 | 1.71 | - |  |  |  |
| (2) | Religiosity | 2.39 | 1.41 | .44** | - |  |  |
| (3) | Religious Conviction | 2.08 | 1.28 | .21** | .52** | - |  |
| (4) | Moral Conviction | 3.54 | 1.06 | -.12** | .14** | .39** | - |

*Note.* * *p* < .05, ** *p* < .01, higher political orientation scores = conservative.

Table S8.2

*Hierarchical Regression Model Predicting Preferred Candidate Moral Conviction*

|  | Predictor | Moral Conviction | |  |
| --- | --- | --- | --- | --- |
|  |  | *B* | *SE* | Δ *R^2^* |
| Block 1  df = 1880 | Political Orientation | -0.13** | 0.01 |  |
|  | Religiosity | 0.02 | 0.02 |  |
|  | Religious Conviction | 0.34** | 0.02 | .19** |
| Block 2  df = 1877 | Religiosity X Religious Conviction | 0.02 | 0.01 |  |
|  | Political Orientation X Religiosity | 0.04** | 0.01 |  |
|  | Political Orientation X Religious Conviction | 0.03** | 0.01 | .03** |
| Block 3  df = 1876 | Religiosity X Religious Conviction X Political Orientation | -0.003 | 0.01 | .00 |
| *R^2^* |  |  |  | .22** |

* *p* < .05, ** *p* < .01


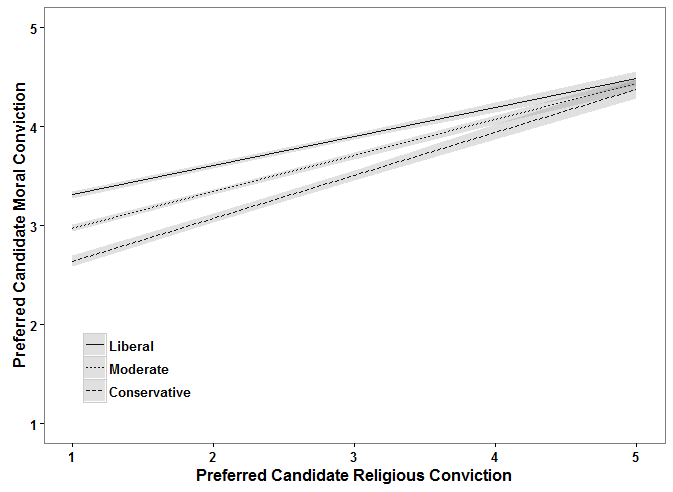


*Figure S8.1.* Simple slopes for the preferred candidate in the 2012 Presidential Election religious conviction by political orientation interaction predicting preferred candidate moral conviction. Political orientation is centered on moderates, 1 *SD* below moderate = liberal, 1 *SD* above moderate = conservative. Ribbons indicate standard error.

Table S8.3

*Simple Slopes for the Religious Conviction by Political Orientation Interaction Predicting Preferred Candidate in the 2012 Presidential Election Moral Conviction.*

| Level of Moderator | Religious Conviction predicting Moral Conviction | | | |
| --- | --- | --- | --- | --- |
|  | *B* | *SE* | *t* | *p* |
| Liberal  (1 *SD* below moderate) | 0.29 | 0.02 | 13.02 | <.001 |
| Moderate | 0.36 | 0.02 | 17.99 | <.001 |
| Conservative  (1 *SD* above moderate) | 0.44 | 0.03 | 14.09 | <.001 |

*Note.* df = 1878

Though not relevant for our hypotheses, there was also an interaction between political orientation and religiosity predicting preferred candidate moral conviction (see Table S8.2). There was a positive relationship between religiosity and moral conviction for conservatives and no relationship between religiosity and moral conviction for moderates or liberals (see Figure S8.2 and Table S8.4).


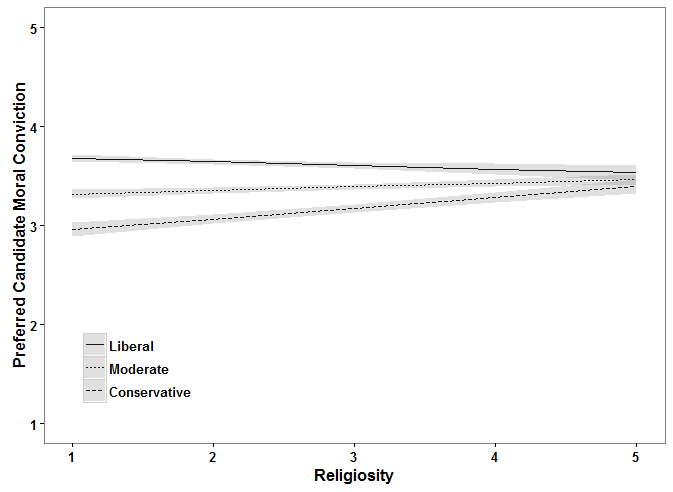


*Figure S8.2.* Simple slopes for the religiosity by political orientation interaction predicting preferred candidate in the 2012 Presidential Election moral conviction. Political orientation is centered on moderates, 1 *SD* below moderate = liberal, 1 *SD* above moderate = conservative. Ribbons indicate standard error.

Table S8.4

*Simple Slopes for the Religiosity by Political Orientation Interaction Predicting Preferred Candidate in the 2012 Presidential Election Moral Conviction.*

| Level of Moderator | Religiosity predicting Moral Conviction | | | |
| --- | --- | --- | --- | --- |
|  | *B* | *SE* | *t* | *p* |
| Liberal  (1 *SD* below moderate) | -0.04 | 0.02 | -1.57 | .12 |
| Moderate | 0.04 | 0.02 | 1.86 | .06 |
| Conservative  (1 *SD* above moderate) | 0.11 | 0.03 | 3.81 | <.001 |

*Note.* df = 1878

### Non-preferred Candidate in 2012 Election

As can be seen in Table S8.5, moral and religious conviction for non-preferred candidate in 2012 election were moderately correlated, though still below our golden thread hypothesis threshold of .70. As can be seen in Table S8.6, the relationship between religious conviction and moral conviction was moderated by religiosity such that the positive relationship between religious conviction and moral conviction was strongest for those with high religiosity, slightly weaker for those with moderate religiosity, and weakest for those with low religiosity (see Figure S8.3 and Table S8.7). These results most closely support the distinct constructs and secularization hypotheses.

Table S8.5

*Descriptive Statistics and Bi-Variate Correlations for Non-Preferred Candidate in the 2012 Presidential Election*

|  |  | M | SD | (1) | (2) | (3) | (4) |
| --- | --- | --- | --- | --- | --- | --- | --- |
| (1) | Political Orientation | -0.67 | 1.79 | - |  |  |  |
| (2) | Religiosity | 2.39 | 1.48 | .44** | - |  |  |
| (3) | Religious Conviction | 1.83 | 1.29 | .19** | .37** | - |  |
| (4) | Moral Conviction | 2.96 | 1.44 | -.07** | .01 | .45** | - |

*Note.* * *p* < .05, ** *p* < .01, higher political orientation scores = conservative.

Table S8.6

*Hierarchical Regression Model Predicting Non-Preferred Candidate Moral Conviction*

|  | Predictor | Moral Conviction | |  |
| --- | --- | --- | --- | --- |
|  |  | *B* | *SE* | Δ *R^2^* |
| Block 1  df = 1877 | Political Orientation | -0.08** | 0.02 |  |
|  | Religiosity | -0.11 | 0.02 |  |
|  | Religious Conviction | 0.56** | 0.02 | .23** |
| Block 2  df = 1874 | Religiosity X Religious Conviction | 0.08** | 0.02 |  |
|  | Political Orientation X Religiosity | 0.05** | 0.01 |  |
|  | Political Orientation X Religious Conviction | 0.01 | 0.02 | .03** |
| Block 3  df = 1873 | Religiosity X Religious Conviction X Political Orientation | -0.01 | 0.01 | .00 |
| *R^2^* |  |  |  | .26** |

* *p* < .05, ** *p* < .01


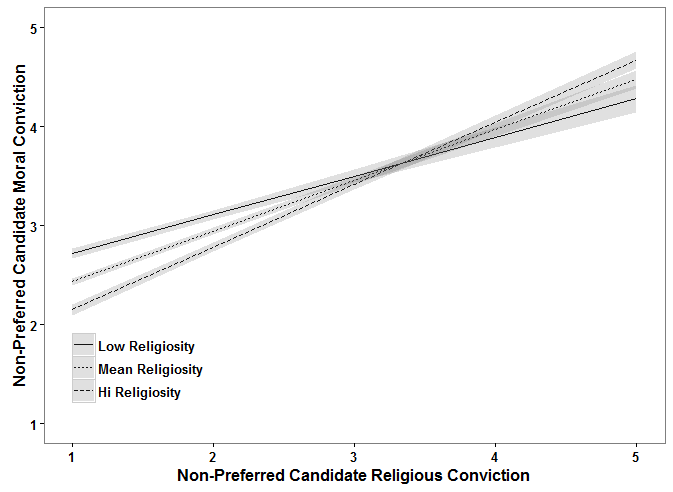


*Figure S8.3.* Simple slopes for the non-preferred candidate in the 2012 Presidential Election religious conviction by religiosity interaction predicting non-preferred candidate moral conviction. Religiosity is mean centered, 1 *SD* below mean = low religiosity, 1 *SD* above mean = high religiosity. Ribbons indicate standard error.

Table S8.7

*Simple Slopes for the Non-Preferred Candidate Religious Conviction by Religiosity Interaction Predicting Non-Preferred Candidate Moral Conviction*

| Level of Moderator | Religious Conviction predicting  Moral Conviction | | | |
| --- | --- | --- | --- | --- |
|  | *B* | *SE* | *t* | *p* |
| Low Religiosity  (1 *SD* below mean) | 0.39 | 0.04 | 10.71 | <.001 |
| Mean Religiosity | 0.51 | 0.02 | 20.71 | <.001 |
| High Religiosity  (1 *SD* above mean) | 0.63 | 0.03 | 22.63 | <.001 |

*Note.* df = 1875

Though not relevant for our hypotheses, there was also an interaction between political orientation and religiosity predicting non-preferred candidate moral conviction (see Table S8.6). There was a negative relationship between religiosity and moral conviction for liberals, a slightly weaker negative relationship for moderates, and no relationship between religiosity and moral conviction for conservatives (see Figure S8.4 and Table S8.8).

*
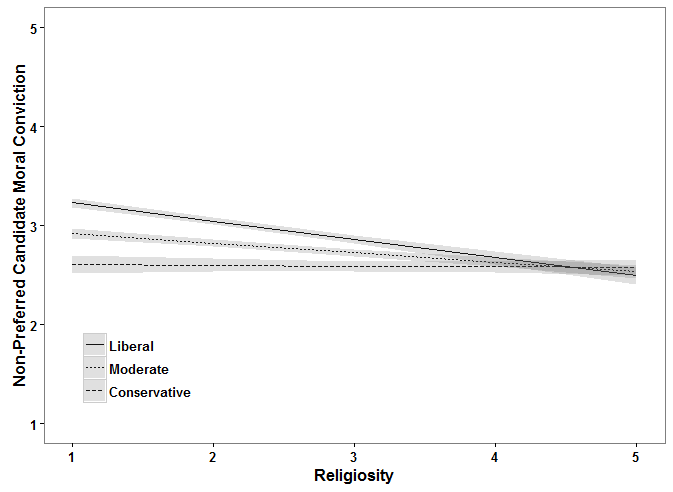
*

*Figure S8.4.* Simple slopes for the religiosity by political orientation interaction predicting non-preferred candidate in the 2012 Presidential Election moral conviction. Political orientation is centered on moderates, 1 *SD* below moderate = liberal, 1 *SD* above moderate = conservative. Ribbons indicate standard error.

Table S8.8

*Simple Slopes for the Religiosity by Political Orientation Interaction Predicting Non-Preferred Candidate in the 2012 Presidential Election Moral Conviction*

| Level of Moderator | Religiosity predicting Moral Conviction | | | |
| --- | --- | --- | --- | --- |
|  | *B* | *SE* | *t* | *p* |
| Liberal  (1 *SD* below moderate) | -0.18 | 0.03 | -6.80 | <.001 |
| Moderate | -0.10 | 0.02 | -4.09 | <.001 |
| Conservative  (1 *SD* above moderate) | -0.01 | 0.03 | -0.25 | .81 |

*Note.* df = 1875

# Study 9: Hanson & Skitka, unpublished data

## Method

Study 9 was a secondary analysis of data originally collected to test whether moral convictions are experienced with a prevention focus (i.e., preventing moral wrongs) or promotion focus (i.e., promoting moral goods). Relevant to the current paper, the data include measures of moral and religious conviction for the issue of legalized abortion, as well as participants’ political orientation. Thus, these data allow us to test the golden thread, culture war (broad form), and distinct constructs hypotheses. This study was approved by the Institutional Review Board of the Office for the Protection of Research Subjects at the University of Illinois at Chicago. Prior to completing any study measures, participants were presented with an informed consent form with information about the study and consented to participate.

### Participants

Four hundred and nineteen participants were recruited from the Introductory Psychology subject pool at the University of Illinois at Chicago. Participants’ ages ranged from 17 to 49 (*M* = 19.22, *SD* = 2.44), were 68.5% female, and 31.5% male.

### Issue Generation

Participants were randomly assigned to one of four writing prompts designed to manipulate the type of topic they would evaluate for the remainder of the study (a moral topic, a practical topic, a topic that is a matter of preference, or a control topic). More specifically, participants saw one of the following four prompts:

1. “There are some political issues that individuals’ feel reflect their beliefs about what is right or wrong, moral or immoral. For these issues, people feel their support or opposition to the issue is a moral stance, rooted in their fundamental beliefs about right and wrong. Please take the next few minutes and think about your stance on a specific political issue that you feel is a reflection of your core moral beliefs and convictions. When you are ready, please write as many sentences as you can to describe why this issue is one you see as a moral issue.”
2. “There are some political issues that individuals’ feel are practical issues. For these issues, people feel their support or opposition to the issue is a pragmatic stance, rooted in the relative costs and benefits of the issue. Please take the next few minutes and think about your stance on a specific political issue that you feel is based on the cost and/or benefits of the issue. When you are ready, please write as many sentences as you can to describe why this issue is one you see as a practical issue.”
3. “There are some political issues that individuals’ feel are a matter of preference. For these issues, people feel their support or opposition to the issue is a personal opinion, and that opinions on the issue may vary. Please take the next few minutes and think about your stance on a specific political issue that you feel is based on just a person opinion. When you are ready, please write as many sentences as you can to describe your stance on the issue.”
4. “Take a few minutes and think about being a subject in a psychology experiment. When you are ready please write as many sentences as you can to describe what it is like to be a subject in a psychology experiment.”

After finishing their assigned writing prompt, participants completed the measures described below.

### Measures

**Moral conviction.** Moral convictions for the issue that participants wrote about in the first part of the study were measured with a validated four-item measure (Skitka & Morgan, 2014). Participants rated the extent to which their feelings about the issue that they previously wrote about were “a reflection of your core moral beliefs and convictions,” “a moral stance,” “based on strong moral principles,” and “a reflection of your fundamental beliefs about right and wrong” on 5-point scales labeled *not at all*, *slightly*, *moderately*, *much*, and *very much* (α = .96).

**Religious conviction.** Religious conviction was measured with a single face-valid item. Participants rated the extent to which their feelings about the issue they previously wrote about were “a reflection of your religious beliefs” on a 5-point scale labeled *not at all*, *slightly*, *moderately*, *much*, and *very much*.

**Political orientation.** Participants’ political orientation was assessed with the question, “Are your political beliefs generally liberal or conservative?” Answer choices included *liberal*, *neutral/neither*, and *conservative*. For participants who initially selected *liberal* or *conservative*, they were then asked, “To what extent are your political beliefs liberal (conservative),” followed by the answer choices *slightly*, *moderately*, *much*, and *very much*. In contrast, participants who initially selected *neutral/neither* were asked, “Do you lean toward liberal or conservative?” Answer choices included *lean toward liberal*, *lean toward conservative*, and *neutral/uncertain*. We aggregated these measures to create a single, bipolar measure of political orientation: -4 (*very much liberal*), -3 (*much liberal*), -2 (*moderately liberal*), -1 (*slightly liberal/lean towards liberal*), 0 (*neutral/uncertain*), 1 (*slightly conservative/lean towards conservative*), 2 (*moderately conservative*), 3 (*much conservative*), and 4 (*very much conservative*).

## Results

Moral and religious conviction were moderately to highly correlated (see Table S9.1), though still below our golden thread hypothesis threshold of .70 a finding and therefore consistent with the distinct constructs hypothesis. Moreover, this relationship was moderated by political orientation. Religious conviction significantly predicted moral conviction at all levels of political orientation, but this pattern was stronger the more conservative participants were (i.e., supportive of the broad culture war hypothesis; see Table S9.2, Table S9.3, and Figure S9.1).

Table S9.1

*Descriptive Statistics and Bi-Variate Correlations for Participant Generated Issue*

|  |  | M | SD | (1) | (2) | (3) |
| --- | --- | --- | --- | --- | --- | --- |
| (1) | Political Orientation | -0.69 | 1.75 | - |  |  |
| (2) | Religious Conviction | 2.14 | 1.45 | .13** | - |  |
| (3) | Moral Conviction | 3.09 | 1.32 | -.03 | .59** | - |

*Note.* * *p* < .05, ** *p* < .01, higher political orientation scores = conservative.

Table S9.2

*Hierarchical Regression Model Predicting Participant Generated Issue Moral Conviction*

|  | Predictor | Moral Conviction | |  |
| --- | --- | --- | --- | --- |
|  |  | *B* | *SE* | Δ *R^2^* |
| Block 1  df = 416 | Political Orientation | -0.08** | 0.03 |  |
|  | Religious Conviction | 0.55** | 0.04 | .36** |
| Block 2  df = 415 | Political Orientation X  Religious Conviction | 0.05* | 0.02 | .01** |
| *R^2^* |  |  |  | .37** |

* *p* < .05, ** *p* < .01


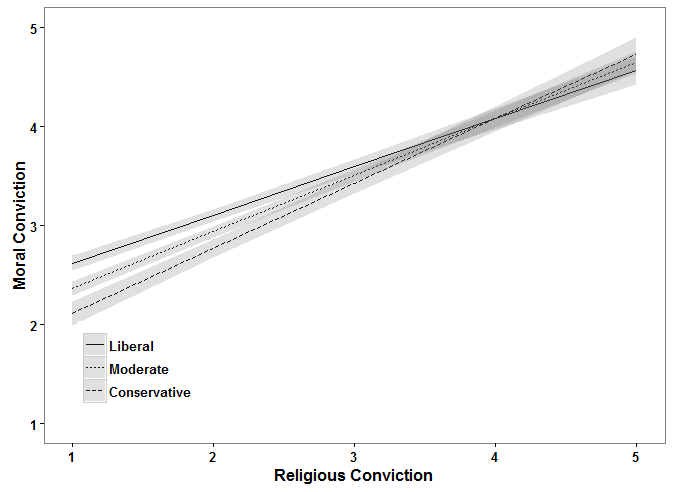


*Figure S9.1.* Simple slopes for the religiosity by political orientation interaction predicting moral conviction. Political orientation is centered on moderates, 1 *SD* below moderate = liberal, 1 *SD* above moderate = conservative. Ribbons indicate standard error.

Table S9.3

*Simple Slopes for the Religious Conviction by Political Orientation Interaction Predicting Moral Conviction*

| Level of Moderator | Religious Conviction predicting  Moral Conviction | | | |
| --- | --- | --- | --- | --- |
|  | *B* | *SE* | *t* | *p* |
| Liberal  (1 *SD* below moderate) | 0.49 | 0.04 | 11.28 | <.001 |
| Moderate | 0.57 | 0.04 | 15.65 | <.001 |
| Conservative  (1 *SD* above moderate) | 0.66 | 0.05 | 12.07 | <.001 |

*Note.* df = 415

# Study 10: Hanson & Skitka, 2016

## Method

This study was approved by the Institutional Review Board of the Office for the Protection of Research Subjects at the University of Illinois at Chicago. Prior to completing any study measures, participants were presented with an informed consent form with information about the study and consented to participate.

### Participants

Participants were recruited from Amazon’s Mechanical Turk website (Mturk) and consisted of 1216 participants who completed relevant measures from wave one of a longitudinal study about attitudes toward the Supreme Court regarding Lesbian, Gay, Bisexual, and Transgendered (LGBT) and affirmative action issues. Participants ranged in age from 18 to 78 (*M* = 36.30, *SD* = 13.01). The same was 45% male and 45% female. Less than one percent had less than a high school education, 11% had a high school degree or GED, 25% had some college but no degree, 11% had an associate’s degree, 30% had a bachelor’s degree, 10% had a master’s degree, and 4% had a doctoral/professional degree.

### Measures

Participants completed measures of moral and religious conviction (interspersed with other items) regarding several LGBT issues (i.e., same-sex marriage, same-sex couple adoption, prohibiting gay boy scout leaders, and LBGT job protection laws) and on affirmative action in college admissions. They also completed a measure of political orientation and religiosity.

**Moral conviction.** Moral convictions associated with the relevant issues were measured with a validated four item measure (see Skitka, Bauman, & Sargis, 2005 for more information about validation). Participants rated the degree to which their feelings about the aforementioned LGBT and affirmative action issues were “a reflection of your fundamental beliefs about right and wrong,” “a reflection of your core moral beliefs and convictions,” “a moral stance,” and “based on strong moral principles?” on 5-point scales labeled *not at all, slightly, moderately, much,* and *very much* (same-sex marriage: α = .93, same-sex adoption: α = .96, gay boy scout leaders: α = .96, LGBT job protection: α = .96, affirmative action: α = .95).

**Religious conviction.** Religious conviction was a measured with two face-valid items, specifically, the extent to which participants indicated that their issue stances were “a reflection of your religious beliefs,” and “a religious stance” with the same response options we used for moral conviction (same-sex marriage: α = .94, same-sex adoption: α = .92, gay Boy Scout leaders: α = .92, LGBT job protection: α = .90, affirmative action: α = .89). The religious and moral conviction items were interspersed with other items assessing attitude strength (e.g., attitude certainty, importance).

**Religiosity.** We used three items from the Santa Clara Strength of Religiosity scale to measure religiosity. Participants were asked how much each of the following states described them: “My religious faith is extremely important to me,” “My religious faith impacts many of my decisions,” and “I look to faith for meaning and purpose in my life,” with the response options of *not at all, slightly, moderately, much,* and *very much* (α = .98).

**Political orientation.** Political orientation was measured by asking participants whether they were conservative, liberal, or neutral/neither. Participants who indicate whether they are liberal or conservative subsequently branched to an item that asked the extent to which they were liberal or conservative, with the response options of *slightly, moderately,* *much*, and *very much* Participants who response with “neutral/neither” branched to an item that asked whether they leaned toward liberal or conservative, or were neutral/uncertain. Leaners were recoded as either *slightly liberal* or *slightly conservative*, and responses to these combined items were used to create a 9-point measure of political orientation, with higher scores reflecting greater conservativism.

## Results

### Same-Sex Marriage

As can be seen in Table S10.1, moral and religious conviction for same-sex marriage were moderately correlated, though still below our golden thread hypothesis threshold of .70. As can be seen in Table S10.2, the relationship between religious conviction and moral conviction was qualified by a three-way interaction with religiosity and political orientation. For conservatives and moderates, the positive relationship between religious conviction and moral conviction was strongest for those with high religiosity, slightly weaker for those with mean levels of religiosity, and weakest or non-significant (conservatives and moderates, respectively) for those with low religiosity (see Figure S10.1 and Table S10.3). In contrast, for liberals the relationship between religious conviction and moral conviction was significantly negative for those low in religiosity, and significantly positive for those at high and mean levels of religiosity. These results most closely support the distinct constructs and secularization hypotheses.

Table S10.1

*Descriptive Statistics and Bi-Variate Correlations for Same-Sex Marriage*

|  |  | M | SD | (1) | (2) | (3) | (4) |
| --- | --- | --- | --- | --- | --- | --- | --- |
| (1) | Political Orientation | -0.38 | 2.53 | - |  |  |  |
| (2) | Religiosity | 2.87 | 1.61 | .43** | - |  |  |
| (3) | Religious Conviction | 2.62 | 1.68 | .45** | .75** | - |  |
| (4) | Moral Conviction | 3.89 | 1.20 | .08** | .26** | .35** | - |

*Note.* * *p* < .05, ** *p* < .01, higher political orientation scores = conservative.

Table S10.2

*Hierarchical Regression Model Predicting Same-Sex Marriage Moral Conviction*

|  | Predictor | Moral Conviction | |  |
| --- | --- | --- | --- | --- |
|  |  | *B* | *SE* | Δ *R^2^* |
| Block 1  df = 1212 | Political Orientation | -0.05** | 0.02 |  |
|  | Religiosity | 0.01 | 0.03 |  |
|  | Religious Conviction | 0.28** | 0.03 | .12** |
| Block 2  df = 1209 | Religiosity X Religious Conviction | 0.11** | 0.02 |  |
|  | Political Orientation X Religiosity | 0.05** | 0.01 |  |
|  | Political Orientation X Religious Conviction | 0.02 | 0.01 | .10** |
| Block 3  df = 1208 | Religiosity X Religious Conviction X Political Orientation | -0.02** | 0.01 | .01** |
| *R^2^* |  |  |  | .22** |

* *p* < .05, ** *p* < .01


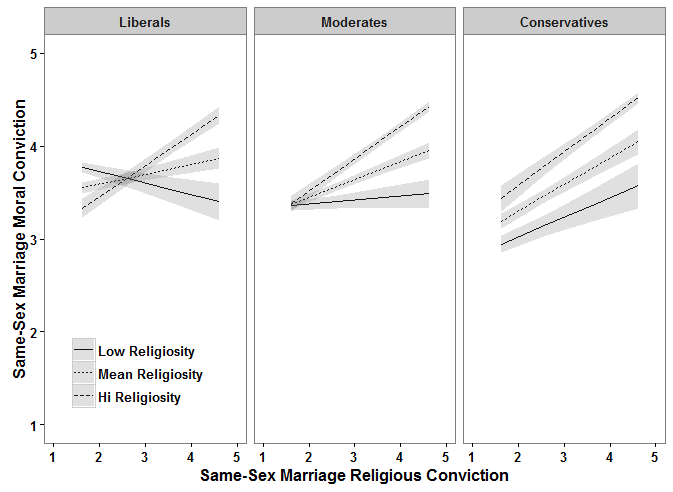


*Figure S10.1.* Simple slopes for the same-sex marriage religious conviction by religiosity by political orientation interaction predicting same-sex marriage moral conviction. Political orientation is centered on moderates, 1 *SD* below moderate = liberal, 1 *SD* above moderate = conservative. Ribbons indicate standard error.

Table S10.3

*Simple Interactions and Slopes for the Same-Sex Marriage Religious Conviction by Religiosity by Political Orientation Interaction Predicting Same-Sex Marriage Moral Conviction*

|  |  | Levels of Political Orientation Moderator | | | | | | | | | | | | | |
| --- | --- | --- | --- | --- | --- | --- | --- | --- | --- | --- | --- | --- | --- | --- | --- |
|  |  | Liberals | | | |  | Moderates | | | |  | Conservatives | | | |
| Simple Interaction |  | *B* | *SE* | *t* | *p* |  | *B* | *SE* | *t* | *p* |  | *B* | *SE* | *t* | *p* |
| Religious Conviction X Religiosity |  | 0.15 | 0.02 | 6.69 | <.001 |  | 0.10 | 0.02 | 5.77 | <.001 |  | 0.05 | 0.03 | 1.92 | 0.06 |
|  |  | Religious Conviction predicting  Moral Conviction | | | |  | Religious Conviction predicting  Moral Conviction | | | |  | Religious Conviction predicting  Moral Conviction | | | |
| Levels of Religiosity Moderator |  | *B* | *SE* | *t* | *p* |  | *B* | *SE* | *t* | *p* |  | *B* | *SE* | *t* | *p* |
| Low Religiosity  (1 *SD* below mean) |  | -0.12 | 0.06 | -2.06 | .03 |  | 0.04 | 0.05 | 0.90 | .37 |  | 0.21 | 0.08 | 2.69 | .007 |
| Mean Religiosity |  | 0.11 | 0.04 | 2.66 | .007 |  | 0.20 | 0.03 | 6.05 | <.001 |  | 0.29 | 0.05 | 5.58 | <.001 |
| High Religiosity  (1 *SD* above mean) |  | 0.33 | 0.05 | 7.44 | <.001 |  | 0.35 | 0.03 | 10.26 | <.001 |  | 0.36 | 0.05 | 7.49 | <.001 |

*Note.* df = 1208, 1 *SD* below midpoint (moderates) = Liberal, 1 *SD* above midpoint (moderates) = Conservatives.

### Same-Sex Couple Adoption

As can be seen in Table S10.4, moral and religious conviction for same-sex couple adoption were weakly to moderately correlated, though still below our golden thread hypothesis threshold of .70. As can be seen in Table S10.5, the relationship between religious conviction and moral conviction was moderated by religiosity such that the positive relationship between religious conviction and moral conviction was strongest for those with high religiosity, slightly weaker for those with moderate religiosity, and non-significant for those with low religiosity (see Figure S10.2 and Table S10.6). These results most closely support the distinct constructs and secularization hypotheses.

Table S10.4

*Descriptive Statistics and Bi-Variate Correlations for Same-Sex Couple Adoption*

|  |  | M | SD | (1) | (2) | (3) | (4) |
| --- | --- | --- | --- | --- | --- | --- | --- |
| (1) | Political Orientation | -0.38 | 2.53 | - |  |  |  |
| (2) | Religiosity | 2.87 | 1.61 | .43** | - |  |  |
| (3) | Religious Conviction | 2.38 | 1.56 | .37** | .70** | - |  |
| (4) | Moral Conviction | 3.78 | 1.28 | -.05 | .13** | .29** | - |

*Note.* * *p* < .05, ** *p* < .01, higher political orientation scores = conservative.

Table S10.5

*Hierarchical Regression Model Predicting Same-Sex Couple Adoption Moral Conviction*

|  | Predictor | Moral Conviction | |  |
| --- | --- | --- | --- | --- |
|  |  | *B* | *SE* | Δ *R^2^* |
| Block 1  df = 1115 | Political Orientation | -0.08** | 0.02 |  |
|  | Religiosity | -0.08** | 0.03 |  |
|  | Religious Conviction | 0.34** | 0.03 | .12** |
| Block 2  df = 1112 | Religiosity X Religious Conviction | 0.11** | 0.02 |  |
|  | Political Orientation X Religiosity | 0.03* | 0.01 |  |
|  | Political Orientation X Religious Conviction | 0.03** | 0.01 | .08** |
| Block 3  df = 1111 | Religiosity X Religious Conviction X Political Orientation | -0.01 | 0.01 | .00 |
| *R^2^* |  |  |  | .20** |

* *p* < .05, ** *p* < .01


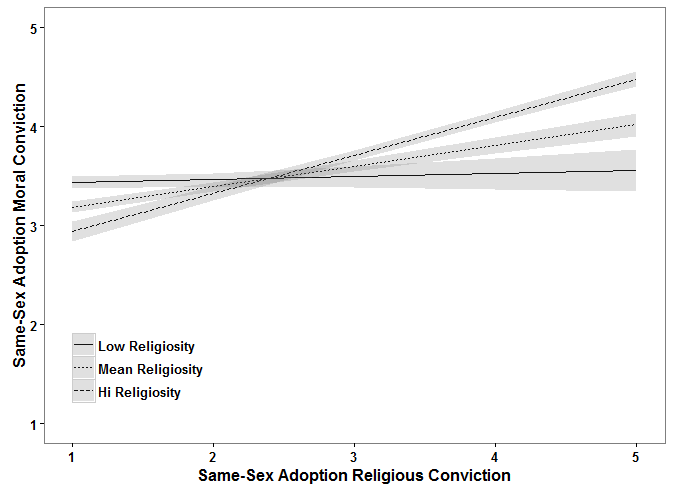


*Figure S10.2.* Simple slopes for the same-sex adoption religious conviction by religiosity interaction predicting same-sex adoption moral conviction. Religiosity is mean centered, 1 *SD* below mean = low religiosity, 1 *SD* above mean = high religiosity. Ribbons indicate standard error.

Table S10.6

*Simple Slopes for the Same-Sex Adoption Religious Conviction by Religiosity Interaction Predicting Same-Sex Adoption Moral Conviction*

| Level of Moderator | Religious Conviction predicting  Moral Conviction | | | |
| --- | --- | --- | --- | --- |
|  | *B* | *SE* | *t* | *P* |
| Low Religiosity  (1 *SD* below mean) | 0.05 | 0.06 | 0.78 | .44 |
| Mean Religiosity | 0.22 | 0.04 | 6.30 | <.001 |
| High Religiosity  (1 *SD* above mean) | 0.40 | 0.03 | 11.60 | <.001 |

*Note.* df = 1112

As can also be seen in Table S10.5, the relationship between religious conviction and moral conviction for same-sex couple adoption was also moderated by political orientation such that the positive relationship between religious conviction and moral conviction was strongest for conservatives, slightly weaker for moderates, and weakest for liberals (see Figure S10.3 and Table S10.7). These results most closely support a weak form of the broad culture war hypothesis.


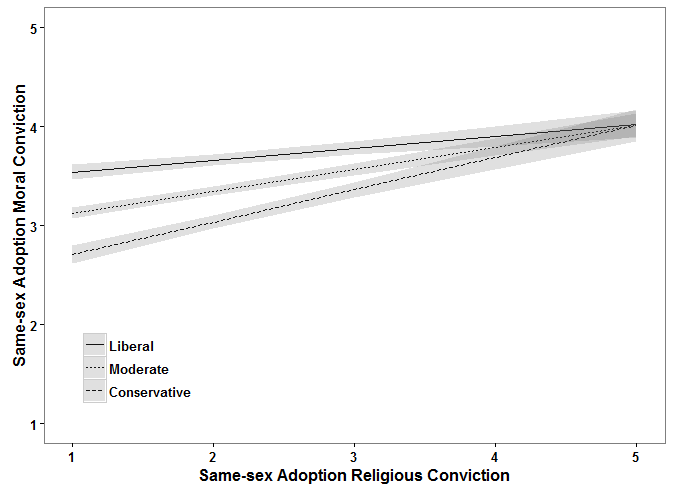


*Figure S10.3.* Simple slopes for the same-sex adoption religious conviction by political orientation interaction predicting same-sex adoption moral conviction. Political orientation is centered on moderates, 1 *SD* below moderate = liberal, 1 *SD* above moderate = conservative. Ribbons indicate standard error.

Table S10.7

*Simple Slopes for the Same-Sex Adoption Religious Conviction by Political Orientation Interaction Predicting Same-Sex Adoption Moral Conviction*

| Level of Moderator | Religious Conviction predicting  Moral Conviction | | | |
| --- | --- | --- | --- | --- |
|  | *B* | *SE* | *t* | *p* |
| Liberal  (1 *SD* below moderate) | 0.12 | 0.04 | 2.75 | .006 |
| Moderate | 0.22 | 0.04 | 6.30 | <.001 |
| Conservative  (1 *SD* above moderate) | 0.32 | 0.05 | 6.41 | <.001 |

*Note.* df = 1112

Though not relevant for our hypotheses, there was also an interaction between political orientation and religiosity predicting same-sex couple adoption moral conviction (see Table S10.6). There was a positive (though marginal) relationship between religiosity and moral conviction for conservatives and no relationship between religiosity and moral conviction for moderates or liberals (see Figure S10.4 and Table S10.8).


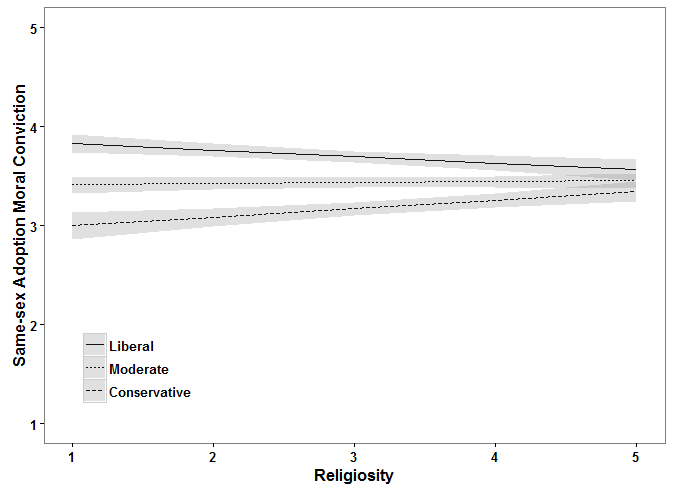


*Figure S10.4.* Simple slopes for the religiosity by political orientation interaction predicting same-sex adoption moral conviction. Political orientation is centered on moderates, 1 *SD* below moderate = liberal, 1 *SD* above moderate = conservative. Ribbons indicate standard error.

Table S10.8

*Simple Slopes for the Religiosity by Political Orientation Interaction Predicting Same-Sex Adoption Moral Conviction*

| Level of Moderator | Religiosity predicting  Moral Conviction | | | |
| --- | --- | --- | --- | --- |
|  | *B* | *SE* | *t* | *p* |
| Liberal  (1 *SD* below moderate) | -0.07 | 0.04 | -1.64 | .10 |
| Moderate | 0.01 | 0.03 | 0.30 | .76 |
| Conservative  (1 *SD* above moderate) | 0.09 | 0.05 | 1.73 | .08 |

*Note.* df = 1112

### Prohibiting Gay Boy Scout Leaders

As can be seen in Table S10.9, moral and religious conviction for attitudes related to prohibiting gay boy scout leaders were moderately correlated, though still below our golden thread hypothesis threshold of .70. As can be seen in Table S10.10, the relationship between religious conviction and moral conviction was moderated by religiosity such that the positive relationship between religious conviction and moral conviction was strongest for those with high religiosity, slightly weaker for those with moderate religiosity, and non-significant for those with low religiosity (see Figure S10.5 and Table S10.11). These results most closely support the distinct constructs and secularization hypotheses.

Table S10.9

*Descriptive Statistics and Bi-Variate Correlations for Prohibiting Gay Boy Scouts Leaders*

|  |  | M | SD | (1) | (2) | (3) | (4) |
| --- | --- | --- | --- | --- | --- | --- | --- |
| (1) | Political Orientation | -0.38 | 2.53 | - |  |  |  |
| (2) | Religiosity | 2.87 | 1.61 | .43** | - |  |  |
| (3) | Religious Conviction | 2.32 | 1.52 | .41** | .70** | - |  |
| (4) | Moral Conviction | 3.71 | 1.28 | .05 | .22** | .35** | - |

*Note.* * *p* < .05, ** *p* < .01, higher political orientation scores = conservative.

Table S10.10

*Hierarchical Regression Model Predicting Prohibiting Gay Boy Scouts Leaders Moral Conviction*

|  | Predictor | Moral Conviction | |  |
| --- | --- | --- | --- | --- |
|  |  | *B* | *SE* | Δ *R^2^* |
| Block 1  df = 1114 | Political Orientation | -0.06** | 0.02 |  |
|  | Religiosity | -0.01 | 0.03 |  |
|  | Religious Conviction | 0.34** | 0.03 | .14** |
| Block 2  df = 1111 | Religiosity X Religious Conviction | 0.10** | 0.02 |  |
|  | Political Orientation X Religiosity | 0.03* | 0.01 |  |
|  | Political Orientation X Religious Conviction | 0.03* | 0.01 | .07** |
| Block 3  df = 1110 | Religiosity X Religious Conviction X Political Orientation | -0.01 | 0.01 | .00 |
| *R^2^* |  |  |  | .21** |

* *p* < .05, ** *p* < .01


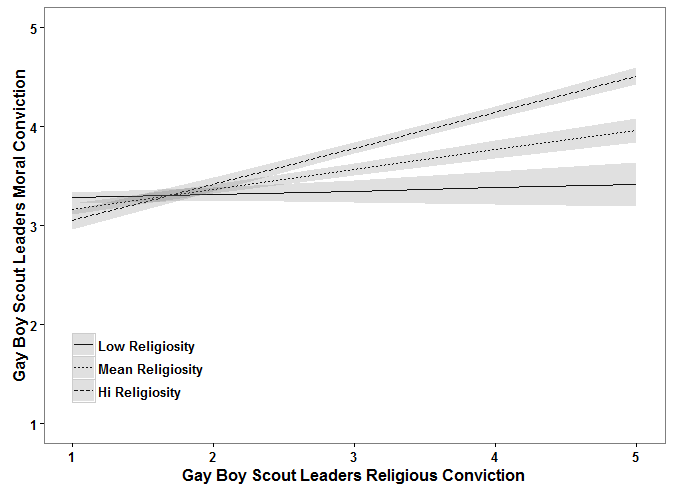


*Figure S10.5.* Simple slopes for the prohibiting gay Boy Scouts leaders religious conviction by religiosity interaction predicting prohibiting gay Boy Scouts leaders moral conviction. Religiosity is mean centered, 1 *SD* below mean = low religiosity, 1 *SD* above mean = high religiosity. Ribbons indicate standard error.

Table S10.11

*Simple Slopes for the Prohibiting Gay Boy Scouts Leaders Religious Conviction by Religiosity Interaction Predicting Prohibiting Gay Boy Scouts Leaders Moral Conviction*

| Level of Moderator | Religious Conviction predicting Moral Conviction | | | |
| --- | --- | --- | --- | --- |
|  | *B* | *SE* | *t* | *P* |
| Low Religiosity  (1 *SD* below mean) | 0.05 | 0.06 | 0.77 | .44 |
| Mean Religiosity | 0.21 | 0.04 | 5.72 | <.001 |
| High Religiosity  (1 *SD* above mean) | 0.38 | 0.04 | 10.62 | <.001 |

*Note.* df = 1111

As can also be seen in Table S10.10, the relationship between religious conviction and moral conviction for prohibiting gay boy scout leaders was also moderated by political orientation such that the positive relationship between religious conviction and moral conviction was strongest for conservatives, slightly weaker for moderates, and weakest for liberals (see Figure S10.6 and Table S10.12). These results most closely support a weak form of the broad culture war hypothesis.


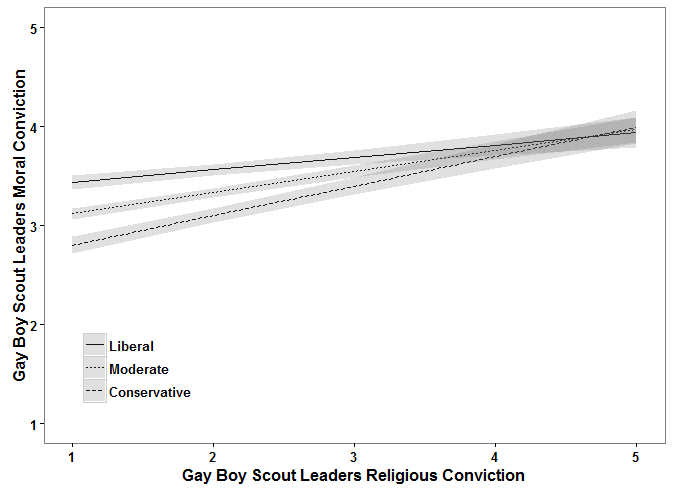


*Figure S10.6.* Simple slopes for the prohibiting gay Boy Scouts leaders religious conviction by political orientation interaction predicting prohibiting gay Boy Scouts leaders moral conviction. Political orientation is centered on moderates, 1 *SD* below moderate = liberal, 1 *SD* above moderate = conservative. Ribbons indicate standard error.

Table S10.12

*Simple Slopes for the Prohibiting Gay Boy Scouts Leaders Religious Conviction by Political Orientation Interaction Predicting Prohibiting Gay Boy Scouts Leaders Moral Conviction*

| Level of Moderator | Religious Conviction predicting Moral Conviction | | | |
| --- | --- | --- | --- | --- |
|  | *B* | *SE* | *t* | *p* |
| Liberal  (1 *SD* below moderate) | 0.13 | 0.05 | 2.64 | .008 |
| Moderate | 0.21 | 0.04 | 5.72 | <.001 |
| Conservative  (1 *SD* above moderate) | 0.30 | 0.05 | 5.71 | <.001 |

*Note.* df = 1111

Though not relevant for our hypotheses, there was also an interaction between political orientation and religiosity predicting prohibiting gay boys scout leaders moral conviction (see Table S10.10). There was a positive relationship between religiosity and moral conviction for conservatives, a slightly weaker positive relationship for moderates, and no relationship between religiosity and moral conviction for liberals (see Figure S10.7 and Table S10.13).


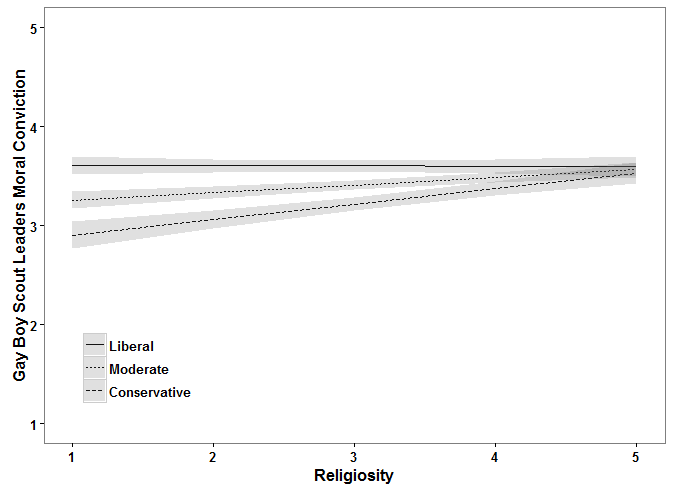


*Figure S10.7.* Simple slopes for the religiosity by political orientation interaction predicting prohibiting gay Boy Scouts leaders moral conviction. Political orientation is centered on moderates, 1 *SD* below moderate = liberal, 1 *SD* above moderate = conservative. Ribbons indicate standard error.

Table S10.13

*Simple Slopes for the Religiosity by Political Orientation Interaction Predicting Prohibiting Gay Boy Scouts Leaders Moral Conviction*

| Level of Moderator | Religiosity predicting Moral Conviction | | | |
| --- | --- | --- | --- | --- |
|  | *B* | *SE* | *t* | *p* |
| Liberal  (1 *SD* below moderate) | -0.002 | 0.04 | -0.06 | .95 |
| Moderate | 0.08 | 0.03 | 2.36 | .02 |
| Conservative  (1 *SD* above moderate) | 0.16 | 0.05 | 3.13 | .002 |

*Note.* df = 1111

### LGBT Job Protection

As can be seen in Table S10.14, moral and religious conviction for attitudes related to LGBT job protection were weakly correlated. As can be seen in Table S10.15, the relationship between religious conviction and moral conviction was moderated by religiosity such that the positive relationship between religious conviction and moral conviction was strongest for those with high religiosity, slightly weaker for those with moderate religiosity, and non-significant for those with low religiosity (see Figure S10.8 and Table S10.16). These results most closely support the distinct constructs and secularization hypotheses.

Table S10.14

*Descriptive Statistics and Bi-Variate Correlations for LGBT Job Protection*

|  |  | M | SD | (1) | (2) | (3) | (4) |
| --- | --- | --- | --- | --- | --- | --- | --- |
| (1) | Political Orientation | -0.38 | 2.53 | - |  |  |  |
| (2) | Religiosity | 2.87 | 1.61 | .43** | - |  |  |
| (3) | Religious Conviction | 2.14 | 1.41 | .23** | .60** | - |  |
| (4) | Moral Conviction | 3.68 | 1.26 | -.20** | .01 | .14** | - |

*Note.* * *p* < .05, ** *p* < .01, higher political orientation scores = conservative.

Table S10.15

*Hierarchical Regression Model Predicting LGBT Job Protection Moral Conviction*

|  | Predictor | Moral Conviction | |  |
| --- | --- | --- | --- | --- |
|  |  | *B* | *SE* | Δ *R^2^* |
| Block 1  df = 1115 | Political Orientation | -0.12** | 0.02 |  |
|  | Religiosity | -0.01 | 0.03 |  |
|  | Religious Conviction | 0.18** | 0.03 | .08** |
| Block 2  df = 1112 | Religiosity X Religious Conviction | 0.05* | 0.02 |  |
|  | Political Orientation X Religiosity | 0.04** | 0.01 |  |
|  | Political Orientation X Religious Conviction | 0.03* | 0.01 | .05** |
| Block 3  df = 1111 | Religiosity X Religious Conviction X Political Orientation | -0.003 | 0.01 | .00 |
| *R^2^* |  |  |  | .12** |

* *p* < .05, ** *p* < .01


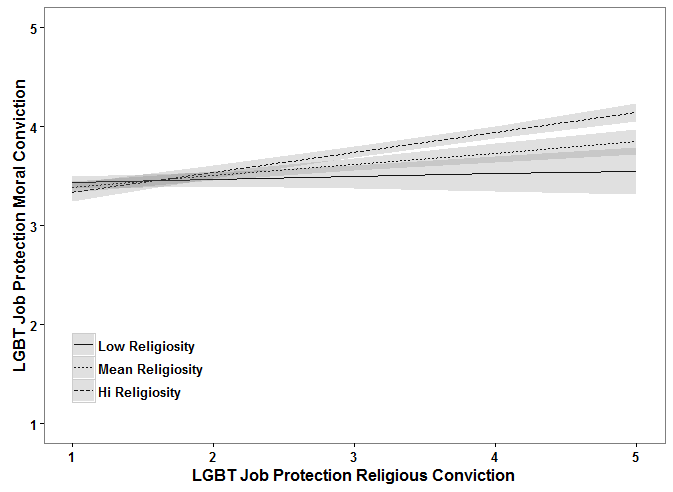


*Figure S10.8.* Simple slopes for the LGBT job protection religious conviction by religiosity interaction predicting LGBT job protection moral conviction. Religiosity is mean centered, 1 *SD* below mean = low religiosity, 1 *SD* above mean = high religiosity. Ribbons indicate standard error.

Table S10.16

*Simple Slopes for the LGBT Job Protection Religious Conviction by Religiosity Interaction Predicting LGBT Job Protection Moral Conviction*

| Level of Moderator | Religious Conviction predicting Moral Conviction | | | |
| --- | --- | --- | --- | --- |
|  | *B* | *SE* | *t* | *P* |
| Low Religiosity  (1 *SD* below mean) | 0.04 | 0.06 | 0.61 | .54 |
| Mean Religiosity | 0.12 | 0.04 | 3.33 | <.001 |
| High Religiosity  (1 *SD* above mean) | 0.21 | 0.03 | 6.05 | <.001 |

*Note.* df = 1112

As can also be seen in Table S10.15, the relationship between religious conviction and moral conviction for LBGT job protection was also moderated by political orientation such that the positive relationship between religious conviction and moral conviction was strongest for conservatives, slightly weaker for moderates, and non-significant for liberals (see Figure S10.9 and Table S10.17). These results most closely support the broad culture war hypothesis.


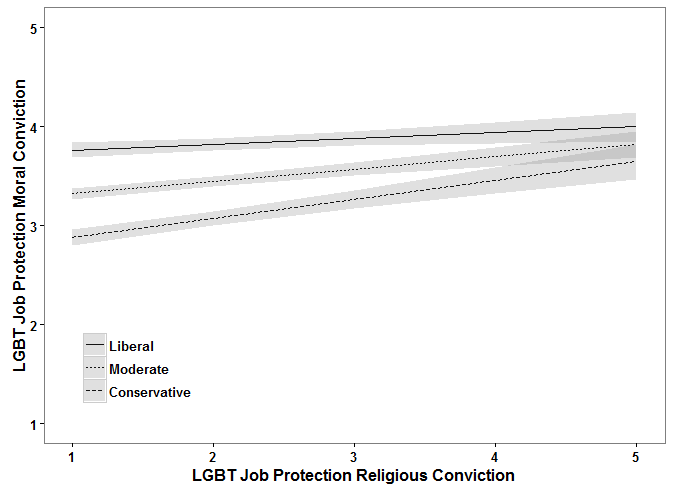


*Figure S10.9.* Simple slopes for the LGBT job protection religious conviction by political orientation interaction predicting LGBT job protection moral conviction. Political orientation is centered on moderates, 1 *SD* below moderate = liberal, 1 *SD* above moderate = conservative. Ribbons indicate standard error.

Table S10.17

*Simple Slopes for the LGBT Job Protection Religious Conviction by Political Orientation Interaction Predicting LGBT Job Protection Moral Conviction*

| Level of Moderator | Religious Conviction predicting Moral Conviction | | | |
| --- | --- | --- | --- | --- |
|  | *B* | *SE* | *t* | *p* |
| Liberal  (1 *SD* below moderate) | 0.06 | 0.05 | 1.27 | .20 |
| Moderate | 0.12 | 0.04 | 3.33 | <.001 |
| Conservative  (1 *SD* above moderate) | 0.19 | 0.05 | 3.61 | <.001 |

*Note.* df = 1112

Though not relevant for our hypotheses, there was also an interaction between political orientation and religiosity predicting LBGT job protection moral conviction (see Table S10.15). There was a positive relationship between religiosity and moral conviction for conservatives and no relationship between religiosity and moral conviction for moderates or liberals (see Figure S10.10 and Table S10.18).


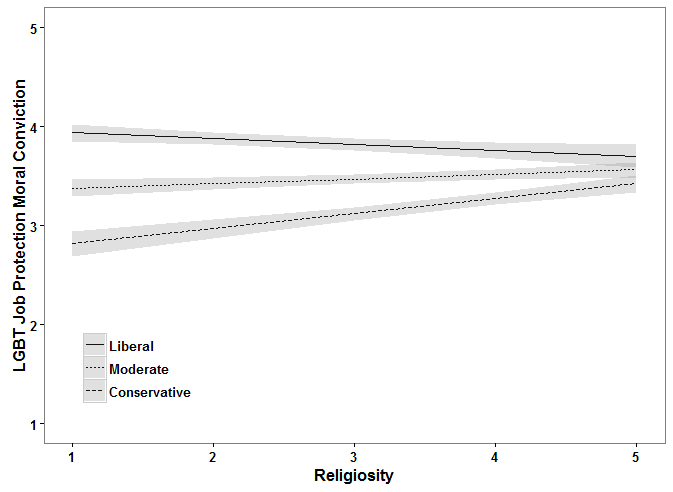


*Figure S10.10.* Simple slopes for the religiosity by political orientation interaction predicting LGBT job protection moral conviction. Political orientation is centered on moderates, 1 *SD* below moderate = liberal, 1 *SD* above moderate = conservative. Ribbons indicate standard error.

Table S10.18

*Simple Slopes for the Religiosity by Political Orientation Interaction Predicting LGBT Job Protection Leaders Moral Conviction*

| Level of Moderator | Religiosity predicting Moral Conviction | | | |
| --- | --- | --- | --- | --- |
|  | *B* | *SE* | *t* | *p* |
| Liberal  (1 *SD* below moderate) | -0.06 | 0.04 | -1.47 | .14 |
| Moderate | 0.05 | 0.03 | 1.49 | .14 |
| Conservative  (1 *SD* above moderate) | 0.15 | 0.04 | 3.42 | <.001 |

*Note.* df = 1112

### Affirmative Action in College Admissions

As can be seen in Table S10.19, moral and religious conviction for attitudes related to affirmative action in college admissions were weakly correlated, though still below our golden thread hypothesis threshold of .70. As can be seen in Table S10.20, the relationship between religious conviction and moral conviction was moderated by religiosity such that the positive relationship between religious conviction and moral conviction was strongest for those with high religiosity, slightly weaker for those with moderate religiosity, and non-significant for those with low religiosity (see Figure S10.11 and Table S10.21). These results most closely support the distinct constructs and secularization hypotheses.

Table S10.19

*Descriptive Statistics and Bi-Variate Correlations for Affirmative Action*

|  |  | M | SD | (1) | (2) | (3) | (4) |
| --- | --- | --- | --- | --- | --- | --- | --- |
| (1) | Political Orientation | -0.38 | 2.53 | - |  |  |  |
| (2) | Religiosity | 2.87 | 1.61 | .43** | - |  |  |
| (3) | Religious Conviction | 1.88 | 1.27 | .15** | .52** | - |  |
| (4) | Moral Conviction | 3.35 | 1.27 | .05 | .17** | .23** | - |

*Note.* * *p* < .05, ** *p* < .01, higher political orientation scores = conservative.

Table S10.20

*Hierarchical Regression Model Predicting Affirmative Action Moral Conviction*

|  | Predictor | Moral Conviction | |  |
| --- | --- | --- | --- | --- |
|  |  | *B* | *SE* | Δ *R^2^* |
| Block 1  df = 1114 | Political Orientation | 0.002 | 0.02 |  |
|  | Religiosity | 0.02 | 0.03 |  |
|  | Religious Conviction | 0.26** | 0.03 | .08** |
| Block 2  df = 1111 | Religiosity X Religious Conviction | 0.08** | 0.03 |  |
|  | Political Orientation X Religiosity | -0.02 | 0.01 |  |
|  | Political Orientation X Religious Conviction | 0.02 | 0.01 | .01** |
| Block 3  df = 1110 | Religiosity X Religious Conviction X Political Orientation | 0.01 | 0.01 | .001 |
| *R^2^* |  |  |  | .09** |

* *p* < .05, ** *p* < .01


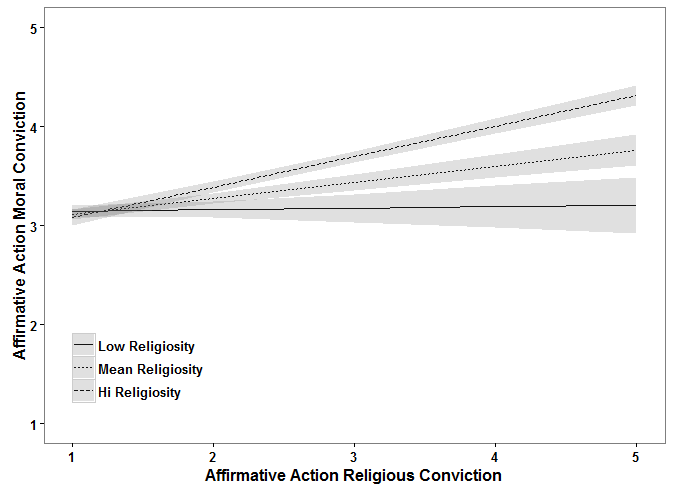


*Figure S10.11.* Simple slopes for the affirmative action religious conviction by religiosity interaction predicting affirmative action moral conviction. Religiosity is mean centered, 1 *SD* below mean = low religiosity, 1 *SD* above mean = high religiosity. Ribbons indicate standard error.

Table S10.21

*Simple Slopes for the Affirmative Action Religious Conviction by Religiosity Interaction Predicting Affirmative Action Moral Conviction*

| Level of Moderator | Religious Conviction predicting Moral Conviction | | | |
| --- | --- | --- | --- | --- |
|  | *B* | *SE* | *t* | *P* |
| Low Religiosity  (1 *SD* below mean) | 0.02 | 0.07 | 0.21 | .84 |
| Mean Religiosity | 0.16 | 0.04 | 3.75 | <.001 |
| High Religiosity  (1 *SD* above mean) | 0.31 | 0.04 | 8.61 | <.001 |

*Note.* df = 1111

# Study 11: Skitka, Hanson, & Wisneski, 2017

## Method

Study 11 was a secondary analysis of data originally collected to test the underlying motivations driving people with strong moral convictions to be politically active for those causes (Skitka et al., 2017). Relevant to the current paper, the data include measures of moral and religious conviction for allowing concealed guns on college campuses, as well as political orientation. Thus, these data allow us to test the golden thread, distinct constructs, and broad culture wars hypotheses. This study was approved by the Institutional Review Board of the Office for the Protection of Research Subjects at the University of Illinois at Chicago. Prior to completing any study measures, participants were presented with an informed consent form with information about the study and consented to participate.

### Participants

Participants (*N* = 1,538) were recruited for a study examining from an online panel maintained by U-SAMP. U-SAMP is a for profit marketing and survey company with over a million volunteer panelists in the U.S. who participate in exchange for direct payment or for donations to charities. We employed quota sampling to ensure that our sample contained roughly similar numbers of males and females, supporters and opponents of legalizing same-sex marriage, and participants from states where same-sex marriage was legal versus illegal at the time of data collection. Participants’ ages ranged from 18 to 82 (*M* = 39.43, *SD* = 14.66), 53% were male and 47% were female, 3% did not complete high school, 19% had a high school diploma or equivalent, 24% attended some college but had no degree, 13% had a technical or associate’s degree, 27% had a bachelor’s degree, 10% had a master’s degree, and 3% had a professional or doctoral degree. This study included measures of moral and religious convictions about same-sex marriage and political orientation, but not religiosity. We could therefore use this study to test the golden thread, culture war (broad form), and distinct constructs hypotheses.

### Measures

**Moral conviction.** Strength of moral conviction about SSM was measured by asking participants the degree to which their feelings about SSM was “a reflection of their core moral beliefs and convictions,” “connected to fundamental beliefs about right and wrong”, “a moral stance,” and “based on a moral principle.” Responses were provided on 5-point scales with point labels *not at all, slightly, moderately, much,* and *very much* (α = .94).

**Religious conviction.** Religious conviction was measured with a single item, “To what extent are your feelings about same-sex marriage a reflection of your religious beliefs,” with a 5-point scale with point labels *not at all, slightly, moderately, much,* and *very much.*

**Political orientation.** To assess political orientation, participants were first asked whether they generally think of themselves as a *liberal, conservative, moderate*, *uncertain/don’t know* or *other*. Participants who indicated that they are liberal or conservative subsequently branched to an item that asked the degree to which they considered themselves a liberal or conservative, with the response options of *slightly liberal/conservative, moderately liberal/conservative,* and *strongly liberal/conservative.* Participants who responded as political moderates, uncertain, or “something else” branched to an item that asked “If you had to choose, would you consider yourself a liberal or a conservative?” with the response options *liberal, neither, conservative.* Leaners were recoded as either *slightly liberal* or *slightly conservative*, and responses to these combined items were used to create a 7-point measure of political orientation, with higher scores reflecting stronger conservatism.

## Results

As can be seen in Table S11.1, moral and religious conviction for attitudes related to affirmative action in college admissions were moderately to highly correlated, though still below our golden thread hypothesis threshold of .70. As can be seen in Table S11.2, the relationship between religious conviction and moral conviction was moderated by political orientation such that the positive relationship between religious conviction and moral conviction was strongest for conservatives, slightly weaker for moderates, and weakest for liberals (see Figure S11.1 and Table S11.3). These results most closely support a modified version of the broad culture war hypothesis and secularization hypothesis.

Table S11.1

*Descriptive Statistics and Bi-Variate Correlations for Same-Sex Marriage*

|  |  | M | SD | (1) | (2) | (3) |
| --- | --- | --- | --- | --- | --- | --- |
| (1) | Political Orientation | 0.27 | 1.73 | - |  |  |
| (2) | Religious Conviction | 2.85 | 1.68 | .37** | - |  |
| (3) | Moral Conviction | 3.58 | 1.26 | .15** | .58** | - |

*Note.* * *p* < .05, ** *p* < .01, higher political orientation scores = conservative.

Table S11.2

*Hierarchical Regression Model Predicting Participant Same-Sex Marriage Moral Conviction*

|  | Predictor | Moral Conviction | |  |
| --- | --- | --- | --- | --- |
|  |  | *B* | *SE* | Δ *R^2^* |
| Block 1  df = 1535 | Political Orientation | -0.06** | 0.02 |  |
|  | Religious Conviction | 0.46** | 0.02 | .34** |
| Block 2  df = 1534 | Political Orientation X  Religious Conviction | 0.07** | 0.01 | .03** |
| *R^2^* |  |  |  | .37** |

* *p* < .05, ** *p* < .01


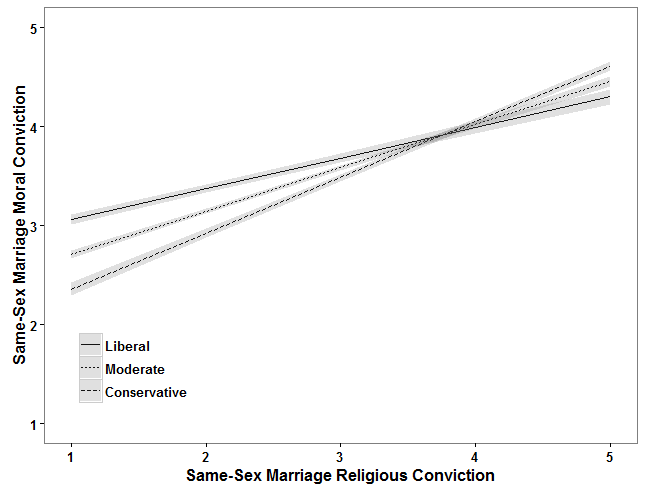


*Figure S11.1.* Simple slopes for the same-se marriage religious conviction by political orientation interaction predicting same-sex marriage moral conviction. Political orientation is centered on moderates, 1 *SD* below moderate = liberal, 1 *SD* above moderate = conservative. Ribbons indicate standard error.

Table S11.3

*Simple Slopes for the Same-Sex Marriage Religious Conviction by Political Orientation Interaction Predicting Same-Sex Marriage Moral Conviction*

| Level of Moderator | Religious Conviction predicting Moral Conviction | | | |
| --- | --- | --- | --- | --- |
|  | *B* | *SE* | *t* | *p* |
| Liberal  (1 *SD* below moderate) | 0.31 | 0.02 | 12.58 | <.001 |
| Moderate | 0.44 | 0.02 | 26.21 | <.001 |
| Conservative  (1 *SD* above moderate) | 0.56 | 0.02 | 27.10 | <.001 |

*Note.* df = 1534

# Study 12: Skitka, Hanson, & Wisneski, 2017

## Method

Study 12 was a secondary analysis of data originally collected to test the underlying motivations driving people with strong moral convictions to be politically active for those causes (Skitka et al., 2017). Relevant to the current paper, the data include measures of moral and religious conviction for allowing concealed guns on college campuses, as well as political orientation. Thus, these data allow us to test the golden thread, distinct constructs, and broad culture wars hypotheses. This study was approved by the Institutional Review Board of the Office for the Protection of Research Subjects at the University of Illinois at Chicago. Prior to completing any study measures, participants were presented with an informed consent form with information about the study and consented to participate.

### Participants

Eight hundred three participants were recruited from Amazon’s Mechanical Turk. Participants were 39.5% female, 50.2% male, and 0.7% indicated another gender identification or preferred not to report their gender, less than 1% did not complete high school, 11% had a high school diploma or equivalent, 29% attended some college but had no degree, 11% had a technical or associate’s degree, 37% had a bachelor’s degree, 8% had a master’s degree, and 2% had a professional or doctoral degree.

### Measures

**Moral conviction.** Moral conviction for guns on college campuses was measured with a validated four-item measure (Skitka & Morgan, 2014). Participants rated the extent to which their feelings about guns on college campuses were “a reflection of your core moral beliefs and convictions,” “a moral stance,” “based on a moral principle,” and “connected to your fundamental beliefs about right and wrong” on 5-point scales labeled *not at all*, *slightly*, *moderately*, *much*, and *very much* (α = .94).

**Religious conviction.** Religious conviction was measured with a single face-valid item. Participants rated the extent to which their feelings about guns on college campuses were “a reflection of your religious beliefs” on a 5-point scale labeled *not at all*, *slightly*, *moderately*, *much*, and *very much*.

**Political orientation.** Participants’ political orientation was assessed with the question, “Generally speaking, do you usually think of yourself as a liberal or a conservative? ” Answer choices included *liberal, conservative, moderate*, *uncertain/don’t know* or *other*. For participants who initially selected *liberal* or *conservative*, they were then asked, “To what degree do you consider yourself liberal/conservative,” followed by the answer choices *slightly* *liberal/conservative*, *moderately liberal/conservative*, and *strongly liberal/conservative.* Participants who initially selected *neither* were asked, “If you had to choose, would you say you lean towards identifying as a conservative or as a liberal?” Answer choices included *lean towards liberal*, *lean towards conservative*, and *neither*. We aggregated these measures to create a single, bipolar measure of political orientation: -3 (*strongly liberal*), -2 (*moderately liberal*), -1 (*slightly liberal/lean towards liberal*), 0 (*neither*), 1 (*slightly conservative/lean towards conservative*), 2 (*moderately conservative*), and 3 (*strongly conservative*).

## Results

Moral and religious conviction for guns on college campuses were moderately correlated (see Table S12.1), which is consistent with the distinct constructs hypothesis. No other hypotheses were supported (see Table S12.2).

Table S12.1

*Descriptive Statistics and Bi-Variate Correlations for Concealed Guns On Campus*

|  |  | M | SD | (1) | (2) | (3) |
| --- | --- | --- | --- | --- | --- | --- |
| (1) | Political Orientation | -0.60 | 1.81 | - |  |  |
| (2) | Religious Conviction | 1.56 | 1.09 | .18** | - |  |
| (3) | Moral Conviction | 3.02 | 1.23 | .02 | .31** | - |

*Note.* * *p* < .05, ** *p* < .01, higher political orientation scores = conservative.

Table S12.2

*Hierarchical Regression Model Predicting Participant Concealed Guns on Campus Moral Conviction*

|  | Predictor | Moral Conviction | |  |
| --- | --- | --- | --- | --- |
|  |  | *B* | *SE* | Δ *R^2^* |
| Block 1  df = 800 | Political Orientation | -0.02 | 0.02 |  |
|  | Religious Conviction | 0.36** | 0.04 | .10** |
| Block 2  df = 799 | Political Orientation X  Religious Conviction | 0.009 | 0.02 | .00 |
| *R^2^* |  |  |  | .10** |

* *p* < .05, ** *p* < .01

# Study 13: Hanson & Skitka, unpublished data

## Method

This study was approved by the Institutional Review Board of the Office for the Protection of Research Subjects at the University of Illinois at Chicago. Prior to completing any study measures, participants were presented with an informed consent form with information about the study and consented to participate.

### Participants

Participants were recruited from the University of Illinois at Chicago Psychology subject pool and consisted of 133 participants whose ages ranged from 17 to 24 (*M* = 19.25, *SD* = 1.17) and were 47% male and 53% female.

### Measures

Participants completed measures of moral and religious conviction (interspersed with other items) regarding their position on capital punishment being legal in all 50 states. They also completed a measure of political orientation.

**Moral conviction.** Moral convictions associated with capital punishment was measured with a validated four item measure (see Skitka, Bauman, & Sargis, 2005 for more information about validation). Participants rated the degree to which their feelings about capital punishment were “a reflection of your fundamental beliefs about right and wrong,” “a reflection of your core moral beliefs and convictions,” “a moral stance,” and “based on strong moral principles” on 5-point scales labeled *not at all, slightly, moderately, much,* and *very much* (α = .87).

**Religious conviction.** Religious conviction was a measured with one face-valid item, specifically, the extent to which participants indicated that their capital punishment stance was “a reflection of your religious beliefs,” with the same response options we used for moral conviction. The religious and moral conviction items were interspersed with other items assessing attitude strength (e.g., attitude certainty, importance).

**Political orientation.** Political orientation was measured by asking participants whether they were conservative, liberal, or neutral/neither. Participants who indicate whether they are liberal or conservative subsequently branched to an item that asked the extent they were liberal or conservative, with the response options of *slightly, moderately, much* and *very much.* Participants who response with “neutral/neither” branched to an item that asked whether they leaned toward liberal or conservative, or neither/uncertain. Leaners were recoded as either *slightly liberal* or *slightly conservative*, and responses to these combined items were used to create a 9-point measure of political orientation, with higher scores reflecting greater conservativism.

## Results

As can be seen in Table S13.1, moral and religious conviction for attitude positions on capital punishment were moderate to moderately correlated, though still below our golden thread hypothesis threshold of .70. Additionally, as seen in Table S13.2, this relationship was not moderated by political orientation. These results most closely support our distinct constructs hypothesis and do not support our broad culture war hypothesis.

Table S13.1

*Descriptive Statistics and Bi-Variate Correlations for Capital Punishment*

|  |  | M | SD | (1) | (2) | (3) |
| --- | --- | --- | --- | --- | --- | --- |
| (1) | Political Orientation | -0.89 | 1.80 | - |  |  |
| (2) | Religious Conviction | 2.20 | 1.39 | .13 | - |  |
| (3) | Moral Conviction | 3.23 | 0.91 | .10 | .46** | - |

*Note.* * *p* < .05, ** *p* < .01, higher political orientation scores = conservative.

Table S13.2

*Hierarchical Regression Model Predicting Participant Capital Punishment Moral Conviction*

|  | Predictor | Moral Conviction | |  |
| --- | --- | --- | --- | --- |
|  |  | *B* | *SE* | Δ *R^2^* |
| Block 1  df = 130 | Political Orientation | 0.02 | 0.04 |  |
|  | Religious Conviction | 0.30** | 0.05 | .21** |
| Block 2  df = 129 | Political Orientation X  Religious Conviction | 0.005 | 0.03 | .00 |
| *R^2^* |  |  |  | .21** |

* *p* < .05, ** *p* < .01

# Study 14: Hanson & Skitka, unpublished data

## Method

This study was approved by the Institutional Review Board of the Office for the Protection of Research Subjects at the University of Illinois at Chicago. Prior to completing any study measures, participants were presented with an informed consent form with information about the study and consented to participate.

### Participants

Participants were recruited from Amazon’s Mechanical Turk website (Mturk) and consisted of 478 participants who completed relevant measures. Participants ranged in age from 18 to 75 (*M* = 33.47, *SD* = 12.95) and were 49% male and 45% female. One percent had less than a high school education, 13% had a high school diploma or GED, 28% had some college but no degree, 10% had a technical/associates degree, 28% had a bachelor’s degree, 11% had a master’s degree, and 2% had a doctoral/professional degree.

### Measures

Participants completed measures of moral and religious conviction (interspersed with other items, e.g., attitude certainty, importance) regarding their position on same-sex marriage. They also completed a measure of political orientation.

**Moral conviction.** Moral convictions associated with same-sex marriage was measured with a validated four item measure (see Skitka, Bauman, & Sargis, 2005 for more information about validation). Participants rated the degree to which their feelings about capital punishment were “a reflection of your fundamental beliefs about right and wrong,” “a reflection of your core moral beliefs and convictions,” “a moral stance,” and “based on strong moral principles” on 5-point scales labeled *not at all, slightly, moderately, much,* and *very much* (α = .92).

**Religious conviction.** Religious conviction was a measured with one face-valid item, specifically, the extent to which participants indicated that their capital punishment stance was “a reflection of your religious beliefs,” with the same response options we used for moral conviction.

**Political orientation.** Political orientation was measured by asking participants whether they were conservative, liberal, or neutral/neither. Participants who indicate whether they are liberal or conservative subsequently branched to an item that asked the extent they were liberal or conservative, with the response options of *slightly, moderately, much* and *very much.* Participants who response with “neutral/neither” branched to an item that asked whether they leaned toward liberal or conservative, or neither/uncertain. Leaners were recoded as either *slightly liberal* or *slightly conservative*, and responses to these combined items were used to create a 9-point measure of political orientation, with higher scores reflecting greater conservativism.

## Results

As can be seen in Table S14.1, moral and religious conviction for same-sex marriage were weakly to moderately correlated, though still below our golden thread hypothesis threshold of .70. As can be seen in Table S14.2, the relationship between religious conviction and moral conviction for same-sex marriage was moderated by political orientation such that the positive relationship between religious conviction and moral conviction was strongest for conservatives, slightly weaker for moderates, and weakest for liberals (see Figure S14.1 and Table S14.3). These results most closely support the distinct constructs hypothesis and a weak form of the broad culture war hypothesis.

Table S14.1

*Descriptive Statistics and Bi-Variate Correlations for Same-Sex Marriage*

|  |  | M | SD | (1) | (2) | (3) |
| --- | --- | --- | --- | --- | --- | --- |
| (1) | Political Orientation | -0.99 | 2.30 | - |  |  |
| (2) | Religious Conviction | 2.36 | 1.64 | .31** | - |  |
| (3) | Moral Conviction | 3.65 | 1.24 | -.11* | .28** | - |

*Note.* * *p* < .05, ** *p* < .01, higher political orientation scores = conservative.

Table S14.2

*Hierarchical Regression Model Predicting Participant Same-Sex Marriage Moral Conviction*

|  | Predictor | Moral Conviction | |  |
| --- | --- | --- | --- | --- |
|  |  | *B* | *SE* | Δ *R^2^* |
| Block 1  df = 475 | Political Orientation | 0.12** | 0.03 |  |
|  | Religious Conviction | 0.27** | 0.03 | .12** |
| Block 2  df = 474 | Political Orientation X  Religious Conviction | 0.10** | 0.01 | .08** |
| *R^2^* |  |  |  | .20** |

* *p* < .05, ** *p* < .01


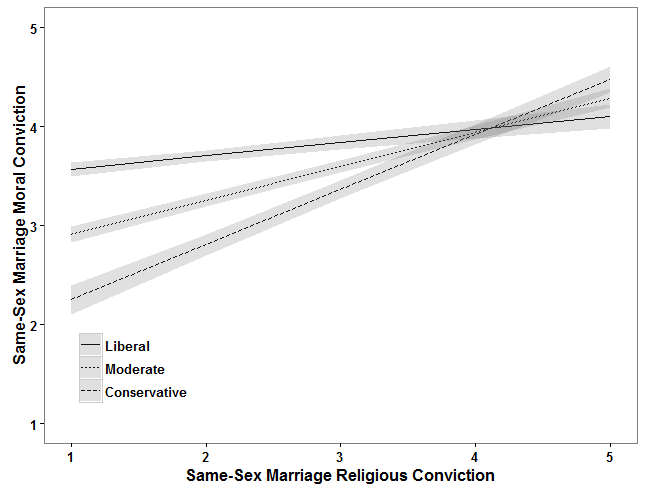


*Figure S14.1.* Simple slopes for the same-sex marriage religious conviction by political orientation interaction predicting same-sex marriage moral conviction. Political orientation is centered on moderates, 1 *SD* below moderate = liberal, 1 *SD* above moderate = conservative. Ribbons indicate standard error.

Table S14.3

*Simple Slopes for the Same-Sex Marriage Religious Conviction by Political Orientation Interaction Predicting Same-Sex Marriage Moral Conviction*

| Level of Moderator | Religious Conviction predicting Moral Conviction | | | |
| --- | --- | --- | --- | --- |
|  | *B* | *SE* | *t* | *p* |
| Liberal  (1 *SD* below moderate) | 0.13 | 0.04 | 3.52 | <.001 |
| Moderate | 0.34 | 0.03 | 9.97 | <.001 |
| Conservative  (1 *SD* above moderate) | 0.56 | 0.05 | 10.48 | <.001 |

*Note.* df = 474

# Study 15: Hanson & Skitka, 2013

## Method

Study 15 was a secondary analysis of data originally collected to test whether moral conviction influences people’s expectations about discussing hot button issues of the day with a political opponent (Hanson et al., 2013). Relevant to the current paper, the data include measures of moral and religious conviction for a participant selected issue, religiosity, and political orientation. Thus, these data allow us to test all four hypotheses. This study was approved by the Institutional Review Board of the Office for the Protection of Research Subjects at the University of Illinois at Chicago. Prior to completing any study measures, participants were presented with an informed consent form with information about the study and consented to participate.

### Participants

Two hundred and twenty-seven participants were recruited from Amazon’s Mechanical Turk. Participants’ ages ranged from 18 to 71 (*M*  = 31.22, *SD* = 12.61), were 40.5% female and 58.1% male. One percent had less than a high school education, 14% had a high school diploma or GED, 37% had some college but no degree, 8% had an associates degree, 33% had a bachelor’s degree, 5% had a master’s degree, and less than 1% had a doctoral/professional degree.

### Issue Selection

Participants were presented with a list of four hot button issues of the day: abortion, capital punishment, nuclear power, and gun control. From that list, they were first asked to select their most moral issue:

1. “From the following list, please select the issue that you feel is most related to your beliefs about what is moral or immoral.”
   1. the availability of legalized abortion in the US
   2. capital punishment (i.e. the continued use of the death penalty)
   3. building new nuclear power plants
   4. stronger gun control laws

Next, participants were asked to select the least moral issue from that list:

1. “From the following list, please select the issue that you feel is least related to your beliefs about what is moral or immoral.”

They then completed the measures described below, for both issues they selected.

### Measures

**Moral conviction.** Moral convictions for each issue was measured with a validated four-item measure (Skitka & Morgan, 2014). Participants rated the extent to which their stance on their two selected issues were “a reflection of your core moral beliefs and convictions,” “a moral stance,” “based on strong moral principles,” and “a reflection of your fundamental beliefs about right and wrong” on 5-point scales labeled *not at all*, *slightly*, *moderately*, *much*, and *very much* (abortion: α = .84, capital punishment: α = .90, gun control: α = .94, nuclear power: α = .90).

**Religious conviction.** Religious conviction was measured with a single face-valid item. Participants rated the extent to which their stance on each issue they selected was “a reflection of your religious beliefs” on a 5-point scale labeled *not at all*, *slightly*, *moderately*, *much*, and *very much*.

**Political orientation.** Political orientation was measured by asking participants whether they were conservative, liberal, or neutral/neither. Participants who indicate whether they are liberal or conservative subsequently branched to an item that asked the extent they were liberal or conservative, with the response options of *slightly, moderately, much* and *very much.* Participants who response with “neutral/neither” branched to an item that asked whether they leaned toward liberal or conservative, or neither/uncertain. Leaners were recoded as either *slightly liberal* or *slightly conservative*, and responses to these combined items were used to create a 9-point measure of political orientation, with higher scores reflecting greater conservativism.

**Religiosity.** Religiosity was assessed with a short form of the Santa Clara Strength of Religious Faith Questionnaire (Plante & Boccaccini, 1997). Participants responded to the stem “To what extent do the following statements apply to you?” followed by the 3 items: “My religious faith is extremely important to me,” “My religious faith impacts many of my decisions,” and “I look to my faith for meaning and purpose in my life.” Participants responded on 5-point scales, with the point labels of *not at all, slightly, moderately, much,* and *very much* (α = .95). Higher scores on this variable indicate greater religiosity.

## Results

### Abortion

Moral and religious conviction were moderately correlated, supporting the distinct constructs hypothesis (see Table S15.1). However, this relationship was qualified by political orientation and religiosity (see Table S15.2). As shown in Figure S15.1 and Table S15.3, religious conviction consistently predicted moral conviction among highly (vs. weakly) religious liberals and moderates (i.e., a finding consistent with the secularization hypothesis). Moreover, religious conviction more strongly predicted moral conviction among conservatives at all levels of religiosity: a finding that is generally consistent with the broad culture war hypothesis.

Table S15.1

*Descriptive Statistics and Bi-Variate Correlations for Abortion*

|  |  | *M* | *SD* | (1) | (2) | (3) | (4) |
| --- | --- | --- | --- | --- | --- | --- | --- |
| (1) | Political Orientation | -0.60 | 2.28 | - |  |  |  |
| (2) | Religiosity | 2.46 | 1.46 | .42** | - |  |  |
| (3) | Religious Conviction | 2.19 | 1.52 | .49** | .76** | - |  |
| (4) | Moral Conviction | 3.72 | 0.99 | .20* | .26** | .42** | - |

*Note.* * *p* < .05, ** *p* < .01, higher political orientation scores = conservative.

Table S15.2

*Hierarchical Regression Model Predicting Abortion Moral Conviction*

|  | Predictor | Moral Conviction | |  |
| --- | --- | --- | --- | --- |
|  |  | *B* | *SE* | Δ *R^2^* |
| Block 1  df = 113 | Political Orientation | -0.002 | 0.04 |  |
|  | Religiosity | -0.09 | 0.09 |  |
|  | Religious Conviction | 0.34** | 0.09 | .18** |
| Block 2  df = 110 | Religiosity X Religious Conviction | 0.13** | 0.05 |  |
|  | Political Orientation X Religiosity | 0.006 | 0.04 |  |
|  | Political Orientation X Religious Conviction | 0.07 | 0.04 | .13** |
| Block 3  df = 109 | Religiosity X Religious Conviction X Political Orientation | -0.05* | 0.02 | .03* |
| *R^2^* |  |  |  | .34** |

* *p* < .05, ** *p* < .01

**
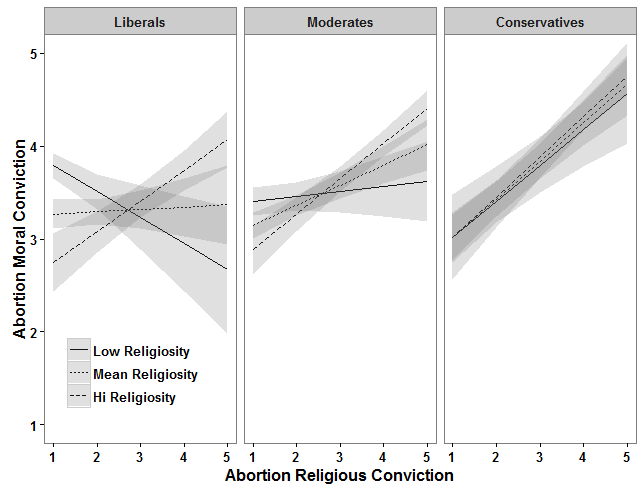
**

*Figure S15.1.* Simple slopes for the abortion religious conviction by religiosity by political orientation interaction predicting abortion moral conviction. Political orientation is centered on moderates, 1 *SD* below moderate = liberal, 1 *SD* above moderate = conservative. Ribbons indicate standard error.

Table S15.3

*Simple Interactions and Slopes for the Abortion Religious Conviction by Religiosity by Political Orientation Interaction Predicting Abortion Moral Conviction*

|  |  | Levels of Political Orientation Moderator | | | | | | | | | | | | | |
| --- | --- | --- | --- | --- | --- | --- | --- | --- | --- | --- | --- | --- | --- | --- | --- |
|  |  | Liberals | | | |  | Moderates | | | |  | Conservatives | | | |
| Simple Interaction |  | *B* | *SE* | *t* | *p* |  | *B* | *SE* | *t* | *p* |  | *B* | *SE* | *t* | *p* |
| Religious Conviction X Religiosity |  | 0.22 | 0.07 | 3.38 | .001 |  | 0.12 | 0.05 | 2.55 | .01 |  | 0.02 | 0.07 | 0.23 | 0.82 |
|  |  | Religious Conviction predicting  Moral Conviction | | | |  | Religious Conviction predicting  Moral Conviction | | | |  | Religious Conviction predicting  Moral Conviction | | | |
| Levels of Religiosity Moderator |  | *B* | *SE* | *t* | *p* |  | *B* | *SE* | *t* | *p* |  | *B* | *SE* | *t* | *p* |
| Low Religiosity  (1 *SD* below mean) |  | -0.28 | 0.18 | -1.57 | .12 |  | 0.05 | 0.12 | 0.46 | .65 |  | 0.39 | 0.16 | 2.46 | .02 |
| Mean Religiosity |  | 0.02 | 0.12 | 0.20 | .84 |  | 0.22 | 0.08 | 2.62 | .01 |  | 0.41 | 0.11 | 3.70 | <.001 |
| High Religiosity  (1 *SD* above mean) |  | 0.33 | 0.12 | 2.65 | .009 |  | 0.38 | 0.09 | 4.23 | <.001 |  | 0.43 | 0.14 | 3.16 | .002 |

*Note.* df = 109, 1 *SD* below midpoint (moderates) = Liberal, 1 *SD* above midpoint (moderates) = Conservatives.

### Capital Punishment

Moral and religious conviction were moderately correlated, but below the .70 golden thread threshold (supportive of the distinct constructs hypothesis, see Table S15.4). However, religiosity moderated this relationship: Religious conviction significantly predicted moral conviction at average and high levels of religiosity, but not at low levels (supportive of the secularization hypothesis, see Figure S15.2, Table S15.5, and Table S15.6).

Table S15.4

*Descriptive Statistics and Bi-Variate Correlations for Capital Punishment*

|  |  | *M* | *SD* | (1) | (2) | (3) | (4) |
| --- | --- | --- | --- | --- | --- | --- | --- |
| (1) | Political Orientation | -0.69 | 2.12 | - |  |  |  |
| (2) | Religiosity | 2.44 | 1.23 | .01 | - |  |  |
| (3) | Religious Conviction | 2.05 | 1.34 | -.13 | .58** | - |  |
| (4) | Moral Conviction | 3.24 | 1.08 | -.02 | .06 | .32** | - |

*Note.* * *p* < .05, ** *p* < .01, higher political orientation scores = conservative.

Table S15.5

*Hierarchical Regression Model Predicting Capital Punishment Moral Conviction*

|  | Predictor | Moral Conviction | |  |
| --- | --- | --- | --- | --- |
|  |  | *B* | *SE* | Δ *R^2^* |
| Block 1  df = 79 | Political Orientation | 0.01 | 0.05 |  |
|  | Religiosity | -0.14 | 0.12 |  |
|  | Religious Conviction | 0.32** | 0.10 | .12** |
| Block 2  df = 76 | Religiosity X Religious Conviction | 0.18* | 0.08 |  |
|  | Political Orientation X Religiosity | 0.04 | 0.06 |  |
|  | Political Orientation X Religious Conviction | -0.02 | 0.04 | .06 |
| Block 3  df = 75 | Religiosity X Religious Conviction X Political Orientation | 0.002 | 0.05 | .00 |
| *R^2^* |  |  |  | .18** |

* *p* < .05, ** *p* < .01


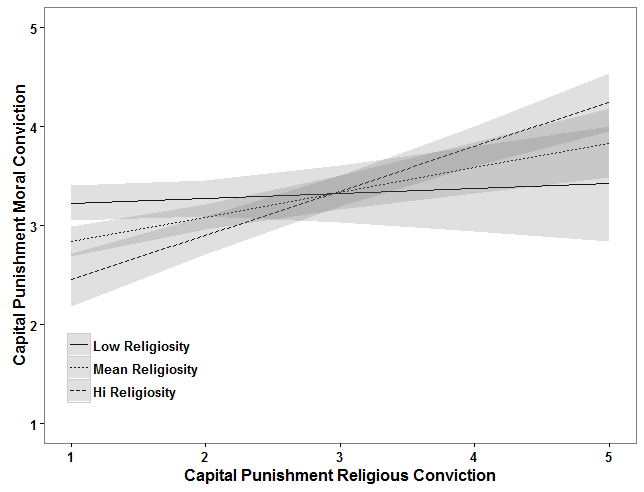


*Figure S15.2.* Simple slopes for the capital punishment religious conviction by religiosity interaction predicting capital punishment moral conviction. Religiosity is mean centered, 1 *SD* below mean = low religiosity, 1 *SD* above mean = high religiosity. Ribbons indicate standard error.

Table S15.6

*Simple Slopes for the Capital Punishment Religious Conviction by Religiosity Interaction Predicting Capital Punishment Moral Conviction*

| Level of Moderator | Religious Conviction predicting Moral Conviction | | | |
| --- | --- | --- | --- | --- |
|  | *B* | *SE* | *t* | *p* |
| Low Religiosity  (1 *SD* below mean) | 0.05 | 0.16 | 0.31 | .75 |
| Mean Religiosity | 0.25 | 0.10 | 2.39 | .02 |
| High Religiosity  (1 *SD* above mean) | 0.45 | 0.11 | 3.92 | <.001 |

*Note.* df = 76

### Nuclear Power

Moral and religious conviction were highly correlated, but below the .70 golden thread threshold (supportive of the distinct constructs hypothesis, see Table S15.7). However, religiosity moderated this relationship: Although religious conviction significantly predicted moral conviction at all levels of religiosity, this relationship was stronger at higher levels of religiosity (supportive of a weak version of the secularization hypothesis, see Table S15.8, Table S15.9, and Figure S15.3).

Table S15.7

*Descriptive Statistics and Bi-Variate Correlations for Nuclear Power*

|  |  | *M* | *SD* | (1) | (2) | (3) | (4) |
| --- | --- | --- | --- | --- | --- | --- | --- |
| (1) | Political Orientation | -0.78 | 2.29 | - |  |  |  |
| (2) | Religiosity | 2.47 | 1.34 | .25** | - |  |  |
| (3) | Religious Conviction | 1.53 | 1.12 | -.08 | .23** | - |  |
| (4) | Moral Conviction | 2.08 | 1.15 | .03 | .18* | .61** | - |

*Note.* * *p* < .05, ** *p* < .01, higher political orientation scores = conservative.

Table S15.8

*Hierarchical Regression Model Predicting Nuclear Power Moral Conviction*

|  | Predictor | Moral Conviction | |  |
| --- | --- | --- | --- | --- |
|  |  | *B* | *SE* | Δ *R^2^* |
| Block 1  df = 144 | Political Orientation | 0.04 | 0.03 |  |
|  | Religiosity | 0.02 | 0.06 |  |
|  | Religious Conviction | 0.63** | 0.07 | .38** |
| Block 2  df = 141 | Religiosity X Religious Conviction | 0.11** | 0.06 |  |
|  | Political Orientation X Religiosity | -0.03 | 0.03 |  |
|  | Political Orientation X Religious Conviction | 0.009 | 0.05 | .03* |
| Block 3  df = 140 | Religiosity X Religious Conviction X Political Orientation | 0.01 | 0.03 | .00 |
| *R^2^* |  |  |  | .41** |

* *p* < .05, ** *p* < .01


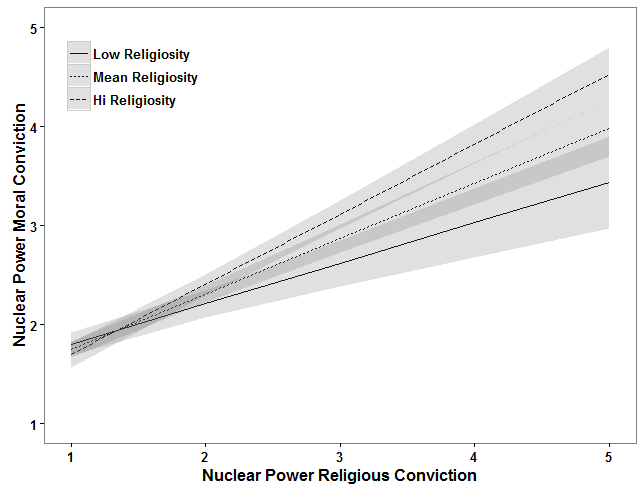


*Figure S15.3.* Simple slopes for the nuclear power religious conviction by religiosity interaction predicting nuclear power moral conviction. Religiosity is mean centered, 1 *SD* below mean = low religiosity, 1 *SD* above mean = high religiosity. Ribbons indicate standard error.

Table S15.9

*Simple Slopes for the Nuclear Power Religious Conviction by Religiosity Interaction Predicting Nuclear Power Moral Conviction*

| Level of Moderator | Religious Conviction predicting Moral Conviction | | | |
| --- | --- | --- | --- | --- |
|  | *B* | *SE* | *t* | *P* |
| Low Religiosity  (1 *SD* below mean) | 0.41 | 0.12 | 3.30 | <.001 |
| Mean Religiosity | 0.56 | 0.08 | 7.30 | <.001 |
| High Religiosity  (1 *SD* above mean) | 0.71 | 0.08 | 9.18 | <.001 |

*Note.* df = 141

### Gun Control

Moral and religious conviction were moderately correlated, supporting the distinct constructs hypothesis (see Table S15.10). No other hypotheses were supported (see Table S15.11).

Table S15.10

*Descriptive Statistics and Bi-Variate Correlations for Gun Control*

|  |  | *M* | *SD* | (1) | (2) | (3) | (4) |
| --- | --- | --- | --- | --- | --- | --- | --- |
| (1) | Political Orientation | -1.00 | 2.04 | - |  |  |  |
| (2) | Religiosity | 2.18 | 1.33 | .19 | - |  |  |
| (3) | Religious Conviction | 1.53 | 1.06 | -.05 | .20 | - |  |
| (4) | Moral Conviction | 2.82 | 1.29 | .06 | -.003 | .45** | - |

*Note.* * *p* < .05, ** *p* < .01, higher political orientation scores = conservative.

Table S15.11

*Hierarchical Regression Model Predicting Gun Control Moral Conviction*

|  | Predictor | Moral Conviction | |  |
| --- | --- | --- | --- | --- |
|  |  | *B* | *SE* | Δ *R^2^* |
| Block 1  df = 220 | Political Orientation | 0.07 | 0.07 |  |
|  | Religiosity | -0.11 | 0.11 |  |
|  | Religious Conviction | 0.58** | 0.13 | .22** |
| Block 2  df = 217 | Religiosity X Religious Conviction | 0.02 | 0.10 |  |
|  | Political Orientation X Religiosity | -0.04 | 0.06 |  |
|  | Political Orientation X Religious Conviction | -0.08 | 0.06 | .03 |
| Block 3  df = 216 | Religiosity X Religious Conviction X Political Orientation | 0.02 | 0.05 | .00 |
| *R^2^* |  |  |  | .25** |

* *p* < .05, ** *p* < .01

# Study 16: Reifen Tagar, Morgan, Halperin, & Skitka, 2013

## Method

Study 16 was a secondary analysis of data originally collected to test whether moral conviction shapes people’s policy preferences toward outgroups in intractable conflict (Reifen Tagar et al., 2013). Relevant to the current paper, the data include measures of moral and religious conviction for the issue of legalized abortion, as well as participants’ political orientation. Thus, these data allow us to test the golden thread, culture war (broad form), and distinct constructs hypotheses. This study was approved by IDC Herzliya University. Prior to completing any study measures, participants were presented with an informed consent form with information about the study and consented to participate.

### Participants

One hundred thirty-six Jewish-Israeli undergraduates started the survey (see Reifen Tagar et al., 2013 for more details). Participants ranged in age from 20 to 46 years old (*M* = 26.66, *SD* = 4.81). The sample was 53% female and politically moderate (*M* = 4.00, *SD* = 1.33 on a scale from 1 (*extreme left*) to 7 (*extreme right*)).

### Measures

For the purposes of this paper, we focused on three measures: Participants’ reported strength of moral and religious conviction for the Israeli-Palestinian conflict (IPC), as well as their political orientation. More detail is provided below.

**Moral conviction.** Moral convictions for the issue of IPC were measured with a three-item measure. Participants rated the following: “To what extent are your feelings about the IPC a reflection of your core moral beliefs and convictions?” “To what extent are your feelings about the IPC deeply connected to your beliefs about ‘right’ and ‘wrong’?” and “To what extent are your attitudes about the IPC a reflection of your core moral values and convictions?” Response choices were on 7-point scales labeled (1) *not at all* and (7) *very much* (α = .71).

**Religious conviction.** Religious conviction was measured with a single face-valid item. Participants rated: “To what extent are your attitudes about the IPC closely connected to your religious beliefs” on a 7-point scale labeled 1 (*not at all*) to 7 (*very high*).

**Political orientation.** Participants reported their political orientation using a single item. Verbal anchors were *extreme left* (1) and *extreme right* (7), with a midpoint of *neither left nor right* (4).

## Results

Moral and religious conviction were weakly correlated, supporting the distinct constructs hypothesis (Table S16.1). No other hypotheses were supported in this study (see Table S16.2).

Table S16.1

*Descriptive Statistics and Correlations of Key Variables in Study 16: Moral and Religious Conviction for Israeli-Palestinian Conflict*

| Variable | *M* | *SD* | 1 | 2 | 3 |
| --- | --- | --- | --- | --- | --- |
| PO | 4.00 | 1.24 | - |  |  |
| RC | 0.00 | 1.91 | .10 | - |  |
| MC | 5.44 | 1.04 | .07 | .20* | - |

**p* < .05. *Note*. MC = moral conviction for I-P Conflict. RC = religious conviction for I-P Conflict. PO = political orientation, ranging from 1 (most left) to 7 (most right).

Table S16.2

*Hierarchical Regression Model Predicting I-P Conflict Moral Conviction*

|  | Predictor | Moral Conviction | |  |
| --- | --- | --- | --- | --- |
|  |  | *B* | *SE* | Δ *R^2^* |
| Block 1  df = 133 | Political Orientation | 0.05 | 0.07 |  |
|  | Religious Conviction | 0.11* | 0.05 | .04† |
| Block 2  df = 132 | Political Orientation X Religious Conviction | 0.05† | 0.03 | .02† |
| *R^2^* |  |  |  | .06* |

†*p* < .10, ** p* < .05, ***p* < .01

*Note:* The interaction of religious conviction and political orientation in predicting moral conviction was marginally significant. Simple slopes analyses revealed a pattern of results that is somewhat consistent with the broad culture wars hypothesis: The effect of religious conviction on moral conviction was stronger for conservatives than liberals (specifically, slope for liberals was *ns* but slopes for moderates and conservatives were significant). Nonetheless, this finding should be interpreted with caution since the finding was not significant per traditional standards (i.e., at the .05 level of significance).

# Study 17: Skitka et al., 2013

## Method

Study 17 was a secondary analysis of data originally collected to test the hypotheses about the connections between moral conviction and social and political intolerance. This study was approved by the Psychology department at Beijing Normal University. Prior to completing any study measures, participants were presented with an informed consent form with information about the study and consented to participate.

### Participants

Participants consisted of a community sample of 100 rural and 110 urban mainland Chinese (*N* = 210). The Chinese sample was 59% female and ranged in age from 14 to 74 (*M* = 36.95, *SD* = 13.51). Participants were recruited for the Chinese version of the study by approaching people in public places and in door-to-door solicitations. Participants were given small gifts of toiletries (e.g., tooth paste) in exchange for their participation. Data was collected using paper-and-pencil questionnaires, and when necessary (i.e., when the respondent was illiterate), the questionnaire was read aloud. Participants were given a list of 16 issues and were asked to select the one issue that was most important to them. The data included measures of moral and religious conviction, and political orientation. Thus, these data allow us to test the golden thread, culture war (broad form), and distinct constructs hypotheses.

### Measures

For the purposes of this paper, we focused on three measures: Participants’ reported strength of moral and religious conviction for their self-selected most important issue, as well as their political orientation. More detail is provided below.

**Moral conviction.** Moral conviction was measured with two items: “To what extent are your feelings about this issue or policy based on your core moral values and convictions?” and “To what extent are your feelings about this issue or policy based on your fundament beliefs about right and wrong?” Both items were measured on 5-point scales labeled *not at all*, *slightly*, *moderately*, *much*, and *very much.* These items correlated at *r* = .80 in China.

**Religious conviction.** Participants rated the extent to which their feelings about their most important issue were “based on a your religious beliefs” on 5-point scales labeled *not at all*, *slightly*, *moderately*, *much*, and *very much*.

**Political orientation.** Participants’ political orientation was assessed with three items. They were asked “In terms of ECONOMIC issues, how would you describe your political views?” In terms of SOCIAL issues, how would you describe your political views? And “How would you describe your GENERAL political outlook?” Participants responded to these items on a 7-point scale with the anchors “*very liberal”* and “*very conservative.”*

## Results

Moral and religious conviction for participant selected issues were moderately correlated but below the .70 golden thread threshold (see Table S17.1), a finding consistent with the distinct constructs hypothesis. No other hypotheses were supported for this issue (see Table S17.2).

Table S17.1

*Descriptive Statistics and Bi-Variate Correlations for Participant Selected Issue*

|  |  | *M* | *SD* | (1) | (2) | (3) |
| --- | --- | --- | --- | --- | --- | --- |
| (1) | Political Orientation | -0.73 | 1.32 | - |  |  |
| (2) | Religious Conviction | 2.54 | 1.35 | -.12 | - |  |
| (3) | Moral Conviction | 3.10 | 1.23 | -.04 | .44** | - |

*Note.* * *p* < .05, ** *p* < .01, higher political orientation scores = conservative.

Table S17.2

*Hierarchical Regression Model Predicting Participant Selected Issue Moral Conviction*

|  | Predictor | Moral Conviction | |  |
| --- | --- | --- | --- | --- |
|  |  | *B* | *SE* | Δ *R^2^* |
| Block 1  df = 204 | Political Orientation | 0.01 | 0.06 |  |
|  | Religious Conviction | 0.40** | 0.06 | .18** |
| Block 2  df = 203 | Political Orientation X  Religious Conviction | -0.03 | 0.05 | .00 |
| *R^2^* |  |  |  | .18** |

* *p* < .05, ** *p* < .01

# Study 18: Skitka et al., 2013

This study was approved by the Institutional Review Board of the Office for the Protection of Research Subjects at the University of Illinois at Chicago. Prior to completing any study measures, participants were presented with an informed consent form with information about the study and consented to participate.

## **Method**

Study 18 contains the same methodology as Study 17, only using (*N* = 595) U.S. Mturk participants. The U.S. sample was recruited on Mechanical Turk, and was 51% female, and ranged in age from 18 to 81 (*M* = 36.23, *SD* = 13.07). The two moral conviction items for the U.S. Mturk sample correlated at *r* = .63. This study was approved by the Institutional Review Board of the Office for the Protection of Research Subjects at the University of Illinois at Chicago. Prior to completing any study measures, participants were presented with an informed consent form with information about the study and consented to participate.

## Results

Moral and religious conviction for participant selected issues were weakly correlated (see Table S18.1), a finding consistent with the distinct constructs hypothesis. No other hypotheses were supported for this issue (see Table S18.2).

Table S18.1

*Descriptive Statistics and Bi-Variate Correlations for Participant Selected Issue*

|  |  | *M* | *SD* | (1) | (2) | (3) |
| --- | --- | --- | --- | --- | --- | --- |
| (1) | Political Orientation | -0.41 | 1.62 | - |  |  |
| (2) | Religious Conviction | 2.11 | 1.41 | .15** | - |  |
| (3) | Moral Conviction | 4.12 | 0.93 | -.08 | .26** | - |

*Note.* * *p* < .05, ** *p* < .01, higher political orientation scores = conservative.

Table S18.2

*Hierarchical Regression Model Predicting Participant Selected Issue Moral Conviction*

|  | Predictor | Moral Conviction | |  |
| --- | --- | --- | --- | --- |
|  |  | *B* | *SE* | Δ *R^2^* |
| Block 1  df = 592 | Political Orientation | -0.07** | 0.02 |  |
|  | Religious Conviction | 0.18** | 0.03 | .08** |
| Block 2  df = 591 | Political Orientation X  Religious Conviction | 0.03 | 0.02 | .01 |
| *R^2^* |  |  |  | .09** |

* *p* < .05, ** *p* < .01

# Study 19: Skitka, Hanson, Washburn, & Mueller, unpublished data

## Method

This study was approved by the Institutional Review Board of the Office for the Protection of Research Subjects at the University of Illinois at Chicago. Prior to completing any study measures, participants were presented with an informed consent form with information about the study and consented to participate.

### Participants

Four hundred twenty-nine participants were recruited from Amazon’s Mechanical Turk in June 2017. Sixteen participants failed both of our instructional manipulation check items and were therefore excluded from subsequent analyses. Our final sample consisted of 413 participants (*M*_age_ = 37.52, *SD*_age_ = 12.04, 42.6% female). 75.1% of the sample described their race/ethnicity as White, 8.7% as Black/African American, 7.7% as Asian/Asian American, 5.8% as Latino/Hispanic/Chicano/Puerto Rican, 1.9% as Multiracial, 1.7% as Middle Eastern/North African, and 1.7% as American Indian/Alaska Native. The sample was slightly liberal on average (*M* = -0.24, *SD* = 2.66). This study was pre-registered at AsPredicted.org, see

### Measures

In this study, participants were asked to report their attitude position, attitude strength, moral and religious conviction, and felt emotions toward seven issues of the day: (1) abstinence only sex education; (2) physician-assisted suicide (allowing people with terminal illnesses to decide with their doctors when to end their lives); (3) allowing abortion to remain a legal option in the U.S.; (4) allowing prayer in public schools; (5) the idea that human activity (e.g., burning fossil fuels) contributes to global warming; (6) raising taxes on the top 1% income earners in the U.S.; and (7) the idea that transgendered people (i.e., people who identify themselves as different than the sex they were born as) should be able to use the public restroom of their choice. They were also asked to report their religiosity and political orientation. More detail is provided below.

**Moral conviction.** Moral convictions for the seven issues were measured with a validated four-item measure (Skitka & Morgan, 2014). Participants rated the extent to which their feelings about legalized abortion were “a reflection of your core moral beliefs and convictions,” “a moral stance,” “based on a moral principle,” and “connected to your beliefs about fundamental right and wrong” on 5-point scales labeled *not at all*, *slightly*, *moderately*, *much*, and *very much* (αs ranged from 0.93 to 0.96 across the seven issues).

**Religious conviction.** Religious conviction was measured with two face-valid items. Participants rated the extent to which their feelings about each issue were “a religious stance,” and “a reflection of your religious beliefs” on 5-point scales labeled *not at all*, *slightly*, *moderately*, *much*, and *very much* (*r*s ranged from 0.85 to 0.94 across the seven issues).

**Religiosity.** Religiosity was assessed with a short form of the Santa Clara Strength of Religious Faith Questionnaire (Plante & Boccaccini, 1997). Participants responded to the stem “To what extent do the following statements apply to you?” followed by the 3 items: “My religious faith is extremely important to me,” “My religious faith impacts many of my decisions,” and “I look to my faith for meaning and purpose in my life.” Participants responded on 5-point scales, with the point labels of *not at all, slightly, moderately, much,* and *very much.*

**Political orientation.** Participants’ political orientation was assessed with the question, “Are your political beliefs generally liberal or conservative?” Answer choices included *liberal*, *neutral/neither*, and *conservative*. For participants who initially selected *liberal* or *conservative*, they were then asked, “To what extent are your political beliefs liberal (conservative),” followed by the answer choices *slightly*, *moderately*, *much*, and *very much*. In contrast, participants who initially selected *neutral/neither* were asked, “If you had to say which way you lean, would you say you are more conservative or more liberal?” Answer choices included *lean toward liberal*, *lean toward conservative*, and *neutral/neither*. We aggregated these measures to create a single, bipolar measure of political orientation: -4 (*very much liberal*), -3 (*much liberal*), -2 (*moderately liberal*), -1 (*slightly liberal/lean liberal*), 0 (*neutral/neither*), +1 (*slightly conservative/lean conservative*), +2 (*moderately conservative*), +3 (*much conservative*), and +4 (*very much conservative*).

## Results

### Abstinence-Only Sex Education

Moral and religious were weakly to moderately correlated (see Table S19.1), a finding consistent with the distinct constructs hypothesis. Moreover, this relationship was moderated by religiosity. Although religious conviction significantly predicted moral conviction at each level of religiosity, the relationship was stronger at higher levels of religiosity (i.e., supportive of the secularization hypothesis; see Table S19.2, Table S19.3, and Figure S19.1**).**

Table S19.1

*Descriptive Statistics and Bivariate Correlations for Abstinence-Only Sex Education*

|  |  | *M* | *SD* | (1) | (2) | (3) | (4) |
| --- | --- | --- | --- | --- | --- | --- | --- |
| (1) | Political Orientation | -0.24 | 2.62 | - |  |  |  |
| (2) | Religiosity | 2.38 | 1.51 | .46** | - |  |  |
| (3) | Religious Conviction | 1.94 | 1.38 | .39** | .72** | - |  |
| (4) | Moral Conviction | 3.08 | 1.29 | -.01 | .23** | .31** | - |

*Note.* * *p* < .05. ** *p* < .01. Higher scores on political orientation indicate greater conservatism.

Table S19.2

*Hierarchical Regression Model Predicting Abstinence-Only Sex Education Moral Conviction*

|  | Predictor | Moral Conviction | |  |
| --- | --- | --- | --- | --- |
|  |  | *B* | *SE* | Δ *R^2^* |
| Block 1  df = 409 | Political Orientation | -0.10** | 0.02 |  |
|  | Religiosity | -0.16** | 0.05 |  |
|  | Religious Conviction | 0.66** | 0.06 | .30** |
| Block 2  df = 406 | Religiosity X Religious Conviction | 0.08** | 0.04 |  |
|  | Political Orientation X Religiosity | 0.03 | 0.02 |  |
|  | Political Orientation X Religious Conviction | 0.04† | 0.02 | .05** |
| Block 3  df = 405 | Religiosity X Religious Conviction X Political Orientation | 0.00 | 0.01 | .00 |
| *R^2^* |  |  |  | .35** |

† *p* < .10, * *p* < .05, ** *p* < .01


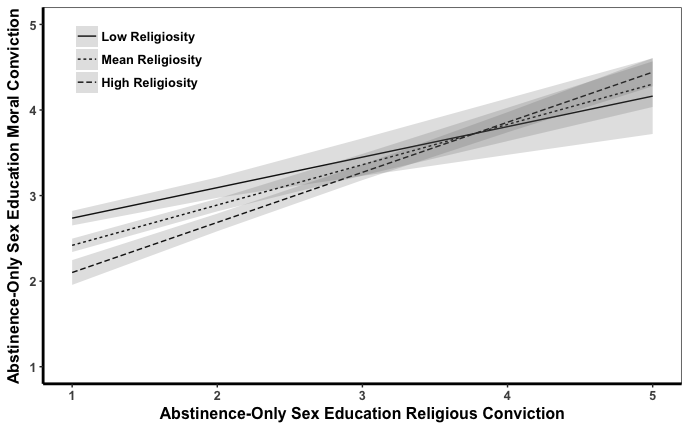


*Figure S19.1.* Simple slopes for the religious conviction by religiosity interaction predicting abstinence-only sex education moral conviction. Religiosity is mean-centered, 1 *SD* below the mean is considered low religiosity, 1 *SD* above the mean is considered high religiosity. Ribbons indicate standard error.

Table S19.3

*Simple Slopes for the Religious Conviction by Religiosity Interaction Predicting Abstinence-Only Sex Education Moral Conviction*

|  | Religious Conviction Predicting Moral Conviction | | | |
| --- | --- | --- | --- | --- |
| Level of Moderator | *B* | *SE* | *t* | *p* |
| Low Religiosity  (1 *SD* below mean) | 0.37 | 0.12 | 3.15 | .002 |
| Mean Religiosity | 0.48 | 0.07 | 6.46 | < .001 |
| High Religiosity  (1 *SD* above mean) | 0.59 | 0.06 | 9.80 | < .001 |

*Note.* df = 406.

### Physician-Assisted Suicide

Moral and religious were weakly to moderately correlated (see Table S19.4), a finding consistent with the distinct constructs hypothesis. Moreover, this relationship was moderated by religiosity. Religious conviction significantly predicted moral conviction at average and high levels of religiosity, but not at low levels (i.e., supportive of the secularization hypothesis; see Table S19.5, Table S19.6, and Figure S19.2**).**

Table S19.4

*Descriptive Statistics and Bivariate Correlations for Physician-Assisted Suicide*

|  |  | *M* | *SD* | (1) | (2) | (3) | (4) |
| --- | --- | --- | --- | --- | --- | --- | --- |
| (1) | Political Orientation | -0.24 | 2.62 | - |  |  |  |
| (2) | Religiosity | 2.38 | 1.51 | .46** | - |  |  |
| (3) | Religious Conviction | 2.05 | 1.46 | .40** | .72** | - |  |
| (4) | Moral Conviction | 3.51 | 1.19 | .03 | .23** | .39** | - |

*Note.* * *p* < .05. ** *p* < .01. Higher scores on political orientation indicate greater conservatism.

Table S19.5

*Hierarchical Regression Model Predicting Physician-Assisted Suicide Moral Conviction*

|  | Predictor | Moral Conviction | |  |
| --- | --- | --- | --- | --- |
|  |  | *B* | *SE* | Δ *R^2^* |
| Block 1  df = 409 | Political Orientation | -0.06** | 0.02 |  |
|  | Religiosity | -0.05 | 0.05 |  |
|  | Religious Conviction | 0.40** | 0.05 | .17** |
| Block 2  df = 406 | Religiosity X Religious Conviction | 0.11** | 0.04 |  |
|  | Political Orientation X Religiosity | 0.05** | 0.02 |  |
|  | Political Orientation X Religious Conviction | 0.01 | 0.02 | .09** |
| Block 3  df = 405 | Religiosity X Religious Conviction X Political Orientation | -0.01 | 0.01 | .00 |
| *R^2^* |  |  |  | .26** |

† *p* < .10, * *p* < .05, ** *p* < .01


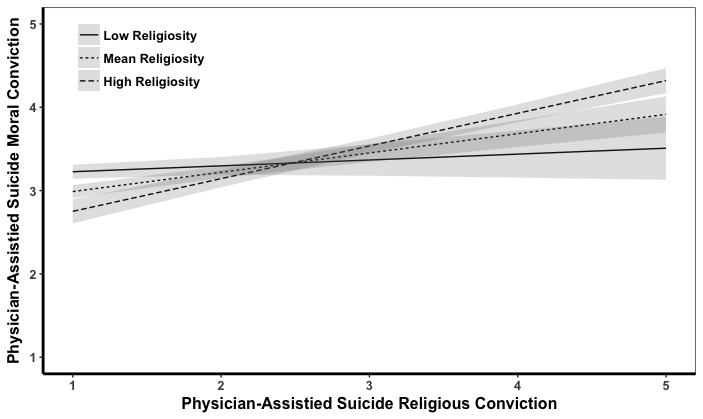


*Figure S19.2.* Simple slopes for the religious conviction by religiosity interaction predicting physician-assisted suicide moral conviction. Religiosity is mean-centered, 1 *SD* below the mean is considered low religiosity, 1 *SD* above the mean is considered high religiosity. Ribbons indicate standard error.

Table S19.6

*Simple Slopes for the Religious Conviction by Religiosity Interaction Predicting Physician-Assisted Suicide Moral Conviction*

|  | Religious Conviction Predicting Moral Conviction | | | |
| --- | --- | --- | --- | --- |
| Level of Moderator | *B* | *SE* | *t* | *p* |
| Low Religiosity  (1 *SD* below mean) | 0.07 | 0.10 | 0.72 | .47 |
| Mean Religiosity | 0.23 | 0.06 | 3.78 | < .001 |
| High Religiosity  (1 *SD* above mean) | 0.39 | 0.06 | 6.93 | < .001 |

*Note.* df = 406.


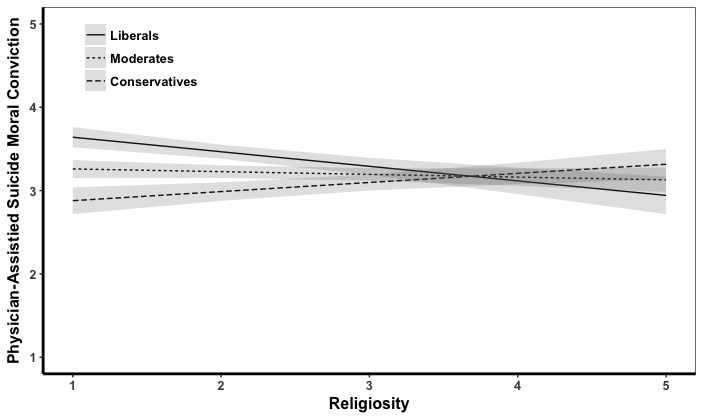


*Figure S19.3.* Simple slopes for the political orientation by religiosity interaction predicting physician-assisted suicide moral conviction. Political orientation is centered on moderates, 1 *SD* below moderate = liberals, 1 *SD* above moderate = conservatives. Ribbons indicate standard error.

Table S19.7

*Simple Slopes for the Political Orientation by Religiosity Interaction Predicting Physician-Assisted Suicide Moral Conviction*

|  | Religiosity Predicting Moral Conviction | | | |
| --- | --- | --- | --- | --- |
| Level of Moderator | *B* | *SE* | *t* | *p* |
| Liberals  (1 *SD* below moderate) | -0.17 | 0.07 | -2.35 | .02 |
| Moderates | -0.03 | 0.05 | -0.63 | .53 |
| Conservatives  (1 *SD* above moderate) | 0.11 | 0.07 | 1.53 | .13 |

*Note.* df = 406.

### Abortion

Moral and religious were weakly to moderately correlated (see Table S19.8), a finding consistent with the distinct constructs hypothesis. Moreover, this relationship was moderated by religiosity. Religious conviction significantly predicted moral conviction at average and high levels of religiosity, but not at low levels (i.e., supportive of the secularization hypothesis; see Table S19.9, Table S19.10, and Figure S19.4**).**

Table S19.8

*Descriptive Statistics and Bivariate Correlations for Abortion*

|  |  | *M* | *SD* | (1) | (2) | (3) | (4) |
| --- | --- | --- | --- | --- | --- | --- | --- |
| (1) | Political Orientation | -0.24 | 2.62 | - |  |  |  |
| (2) | Religiosity | 2.38 | 1.51 | .46** | - |  |  |
| (3) | Religious Conviction | 2.13 | 1.56 | .42** | .74** | - |  |
| (4) | Moral Conviction | 3.65 | 1.24 | .07 | .21** | .42** | - |

*Note.* * *p* < .05. ** *p* < .01. Higher scores on political orientation indicate greater conservatism.

Table S19.9

*Hierarchical Regression Model Predicting Abortion Moral Conviction*

|  | Predictor | Moral Conviction | |  |
| --- | --- | --- | --- | --- |
|  |  | *B* | *SE* | Δ *R^2^* |
| Block 1  df = 409 | Political Orientation | -0.04† | 0.02 |  |
|  | Religiosity | -0.16** | 0.06 |  |
|  | Religious Conviction | 0.48** | 0.05 | .20** |
| Block 2  df = 406 | Religiosity X Religious Conviction | 0.12** | 0.03 |  |
|  | Political Orientation X Religiosity | 0.07** | 0.02 |  |
|  | Political Orientation X Religious Conviction | -0.01 | 0.02 | .09** |
| Block 3  df = 405 | Religiosity X Religious Conviction X Political Orientation | -0.02† | 0.01 | < .01† |
| *R^2^* |  |  |  | .29** |

† *p* < .10, * *p* < .05, ** *p* < .01


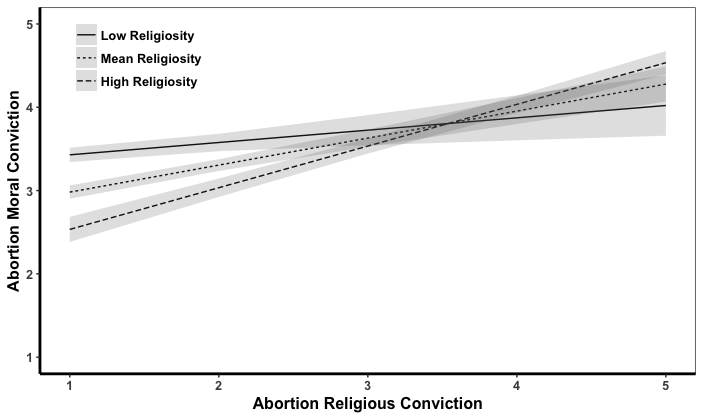


*Figure S19.4.* Simple slopes for the religious conviction by religiosity interaction predicting abortion moral conviction. Religiosity is mean-centered, 1 *SD* below the mean is considered low religiosity, 1 *SD* above the mean is considered high religiosity. Ribbons indicate standard error.

Table S19.10

*Simple Slopes for the Religious Conviction by Religiosity Interaction Predicting Abortion Moral Conviction*

|  | Religious Conviction Predicting Moral Conviction | | | |
| --- | --- | --- | --- | --- |
| Level of Moderator | *B* | *SE* | *t* | *p* |
| Low Religiosity  (1 *SD* below mean) | 0.15 | 0.10 | 1.52 | .13 |
| Mean Religiosity | 0.32 | 0.06 | 5.34 | < .001 |
| High Religiosity  (1 *SD* above mean) | 0.50 | 0.06 | 9.02 | < .001 |

*Note.* df = 406.


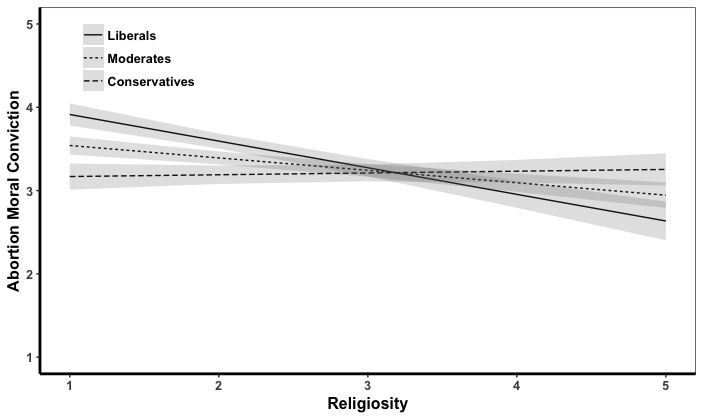


*Figure S19.5.* Simple slopes for the political orientation by religiosity interaction predicting abortion moral conviction. Political orientation is centered on moderates, 1 *SD* below moderate = liberals, 1 *SD* above moderate = conservatives. Ribbons indicate standard error.

Table S19.11

*Simple Slopes for the Political Orientation by Religiosity Interaction Predicting Abortion Moral Conviction*

|  | Religiosity Predicting Moral Conviction | | | |
| --- | --- | --- | --- | --- |
| Level of Moderator | *B* | *SE* | *t* | *p* |
| Liberals  (1 *SD* below moderate) | -0.32 | 0.08 | -4.06 | < .001 |
| Moderates | -0.15 | 0.05 | -2.77 | < .01 |
| Conservatives  (1 *SD* above moderate) | 0.02 | 0.07 | 0.30 | .77 |

*Note.* df = 406.

### Prayer in Public Schools

Moral and religious were moderately to highly correlated (see Table S19.12), a finding consistent with the distinct constructs hypothesis. Moreover, this relationship was moderated by religiosity. Although religious conviction significantly predicted moral conviction at each level of religiosity, the relationship was stronger at higher religiosity levels (i.e., supportive of the secularization hypothesis; see Table S19.13, Table S19.14, and Figure S19.6**).**

Table S19.12

*Descriptive Statistics and Bivariate Correlations for Prayer in Public Schools*

|  |  | *M* | *SD* | (1) | (2) | (3) | (4) |
| --- | --- | --- | --- | --- | --- | --- | --- |
| (1) | Political Orientation | -0.24 | 2.62 | - |  |  |  |
| (2) | Religiosity | 2.38 | 1.51 | .46** | - |  |  |
| (3) | Religious Conviction | 2.45 | 1.59 | .42** | .72** | - |  |
| (4) | Moral Conviction | 3.39 | 1.32 | .14** | .39** | .55** | - |

*Note.* * *p* < .05. ** *p* < .01. Higher scores on political orientation indicate greater conservatism.

Table S19.13

*Hierarchical Regression Model Predicting Prayer in Public Schools Moral Conviction*

|  | Predictor | Moral Conviction | |  |
| --- | --- | --- | --- | --- |
|  |  | *B* | *SE* | Δ *R^2^* |
| Block 1  df = 409 | Political Orientation | -0.06* | 0.02 |  |
|  | Religiosity | 0.03 | 0.05 |  |
|  | Religious Conviction | 0.48** | 0.05 | .32** |
| Block 2  df = 406 | Religiosity X Religious Conviction | 0.08** | 0.03 |  |
|  | Political Orientation X Religiosity | 0.02 | 0.02 |  |
|  | Political Orientation X Religious Conviction | 0.03 | 0.02 | .04** |
| Block 3  df = 405 | Religiosity X Religious Conviction X Political Orientation | -0.01 | 0.01 | < .01 |
| *R^2^* |  |  |  | .36** |

† *p* < .10, * *p* < .05, ** *p* < .01


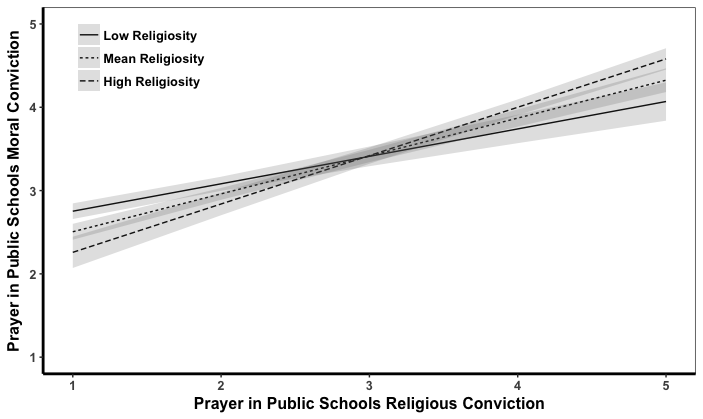


*Figure S19.6.* Simple slopes for the religious conviction by religiosity interaction predicting prayer in public schools moral conviction. Religiosity is mean-centered, 1 *SD* below the mean is considered low religiosity, 1 *SD* above the mean is considered high religiosity. Ribbons indicate standard error.

Table S19.14

*Simple Slopes for the Religious Conviction by Religiosity Interaction Predicting Prayer in Public Schools Moral Conviction*

|  | Religious Conviction Predicting Moral Conviction | | | |
| --- | --- | --- | --- | --- |
| Level of Moderator | *B* | *SE* | *t* | *p* |
| Low Religiosity  (1 *SD* below mean) | 0.34 | 0.07 | 5.12 | < .001 |
| Mean Religiosity | 0.46 | 0.05 | 9.67 | < .001 |
| High Religiosity  (1 *SD* above mean) | 0.59 | 0.06 | 9.29 | < .001 |

*Note.* df = 406.

### Man-Made Climate Change

Moral and religious conviction were weakly to moderately correlated (see Table S19.15), a finding consistent with the distinct constructs hypothesis. No other hypotheses were supported for this issue (see Table S19.16).

Table S19.15

*Descriptive Statistics and Bivariate Correlations for Man-Made Climate Change*

|  |  | *M* | *SD* | (1) | (2) | (3) | (4) |
| --- | --- | --- | --- | --- | --- | --- | --- |
| (1) | Political Orientation | -0.24 | 2.62 | - |  |  |  |
| (2) | Religiosity | 2.38 | 1.51 | .46** | - |  |  |
| (3) | Religious Conviction | 1.51 | 1.01 | .09 | .40** | - |  |
| (4) | Moral Conviction | 2.80 | 1.38 | -.31** | -.09 | .31** | - |

*Note.* * *p* < .05. ** *p* < .01. Higher scores on political orientation indicate greater conservatism.

Table S19.16

*Final Hierarchical Regression Model Predicting Man-Made Climate Change Moral Conviction*

|  |  | Moral Conviction | |  |
| --- | --- | --- | --- | --- |
|  | Predictor | *B* | *SE* | Δ*R*^2^ |
| Block 1  df = 409 | Political Orientation | -0.15** | 0.03 |  |
|  | Religiosity | -0.10* | 0.05 |  |
|  | Religious Conviction | 0.52** | 0.07 | .22** |
| Block 2  df = 406 | Religiosity X Religious Conviction | 0.05 | 0.05 |  |
|  | Political Orientation X Religiosity | 0.03 | 0.02 |  |
|  | Political Orientation X Religious Conviction | 0.02 | 0.03 | .02* |
| Block 3  df = 405 | Religiosity X Religious Conviction X Political Orientation | -0.03 | 0.02 | .003 |
| *R*^2^ |  |  |  | .24** |

*Note*. * *p* < .05. ** *p* < .01. Higher scores on political orientation indicate greater conservatism.

### Taxing the Wealthy

Moral and religious conviction were weakly correlated (see Table S19.17), a finding consistent with the distinct constructs hypothesis. No other hypotheses were supported for this issue (see Table S19.18).

Table S19.17

*Descriptive Statistics and Bivariate Correlations for Taxing the Wealthy*

|  |  | *M* | *SD* | (1) | (2) | (3) | (4) |
| --- | --- | --- | --- | --- | --- | --- | --- |
| (1) | Political Orientation | -0.24 | 2.62 | - |  |  |  |
| (2) | Religiosity | 2.38 | 1.51 | .46** | - |  |  |
| (3) | Religious Conviction | 1.56 | 1.06 | .08 | .40** | - |  |
| (4) | Moral Conviction | 3.22 | 1.30 | -.25** | -.04 | .25** | - |

*Note.* * *p* < .05. ** *p* < .01. Higher scores on political orientation indicate greater conservatism.

Table S19.18

*Final Hierarchical Regression Model Predicting Taxing the Wealthy Moral Conviction*

|  |  | Moral Conviction | |  |
| --- | --- | --- | --- | --- |
|  | Predictor | *B* | *SE* | Δ*R*^2^ |
| Block 1  df = 409 | Political Orientation | -0.13** | 0.03 |  |
|  | Religiosity | -0.02 | 0.05 |  |
|  | Religious Conviction | 0.34** | 0.06 | .14** |
| Block 2  df = 406 | Religiosity X Religious Conviction | 0.09 | 0.05 |  |
|  | Political Orientation X Religiosity | 0.04* | 0.02 |  |
|  | Political Orientation X Religious Conviction | 0.03 | 0.03 | .04** |
| Block 3  df = 405 | Religiosity X Religious Conviction X Political Orientation | 0.002 | 0.02 | .000 |
| *R*^2^ |  |  |  | .18** |

*Note*. * *p* < .05. ** *p* < .01. Higher scores on political orientation indicate greater conservatism.


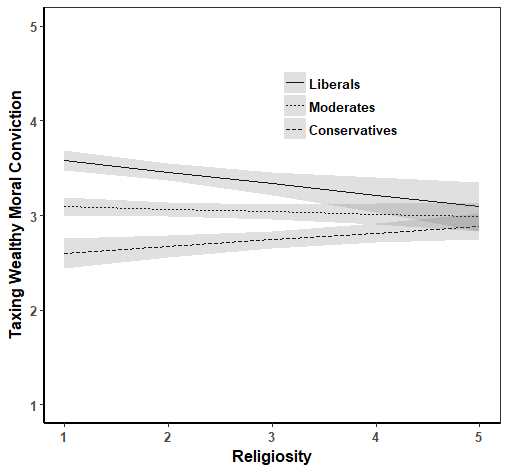


*Figure S19.7.* Simple slopes for the religiosity by political orientation interaction predicting taxing the wealthy moral conviction from Study 19. Political orientation is centered on moderates, 1 *SD* below moderate = liberals, 1 *SD* above moderate = conservatives. Ribbons indicate standard error.

Table S19.19

*Simple Slopes for the Religiosity by Political Orientation Interaction Predicting Taxing the Wealthy Moral Conviction*

|  | Religious Conviction Predicting Moral Conviction | | | |
| --- | --- | --- | --- | --- |
| Level of Moderator | *B* | *SE* | *t* | *p* |
| Liberals  (1 *SD* below moderate) | -0.12 | 0.08 | -1.58 | .12 |
| Moderates | -0.03 | 0.05 | -0.50 | .62 |
| Conservatives  (1 *SD* above moderate) | 0.07 | 0.06 | 1.21 | .23 |

*Note.* df = 406.

### Transgender Bathroom Choice

Moral and religious conviction were moderately correlated (see Table S19.20), a finding consistent with the distinct constructs hypothesis. Moreover, this relationship was moderated by political orientation. Religious conviction significantly predicted moral conviction among moderates and conservatives, but not among liberals (i.e., supportive of the broad culture war hypothesis; see Table S19.21, Table S19.22, and Figure S19.8).

Table S19.20

*Descriptive Statistics and Bivariate Correlations for Transgender Bathroom Choice*

|  |  | *M* | *SD* | (1) | (2) | (3) | (4) |
| --- | --- | --- | --- | --- | --- | --- | --- |
| (1) | Political Orientation | -0.24 | 2.62 | - |  |  |  |
| (2) | Religiosity | 2.38 | 1.51 | .46** | - |  |  |
| (3) | Religious Conviction | 1.99 | 1.44 | .39** | .68** | - |  |
| (4) | Moral Conviction | 3.43 | 1.37 | .02 | .22** | .38** | - |

*Note.* * *p* < .05. ** *p* < .01. Higher scores on political orientation indicate greater conservatism.

Table S19.21

*Final Hierarchical Regression Model Predicting Transgender Bathroom Choice Moral Conviction*

|  |  | Moral Conviction | |  |
| --- | --- | --- | --- | --- |
|  | Predictor | *B* | *SE* | Δ*R*^2^ |
| Block 1  df = 409 | Political Orientation | -0.08** | 0.03 |  |
|  | Religiosity | -0.01 | 0.06 |  |
|  | Religious Conviction | 0.43** | 0.06 | .17** |
| Block 2  df = 406 | Religiosity X Religious Conviction | 0.04 | 0.04 |  |
|  | Political Orientation X Religiosity | 0.01 | 0.02 |  |
|  | Political Orientation X Religious Conviction | 0.06** | 0.02 | .04** |
| Block 3  df = 405 | Religiosity X Religious Conviction X Political Orientation | -0.02 | 0.02 | .003 |
| *R*^2^ |  |  |  | .21** |

*Note*. * *p* < .05. ** *p* < .01. Higher scores on political orientation indicate greater conservatism.


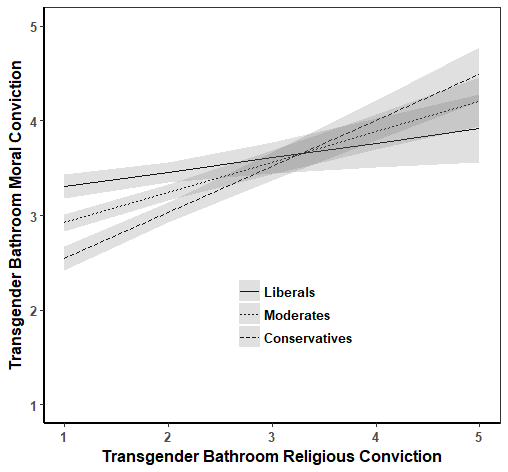


*Figure S19.8.* Simple slopes for the religious conviction by political orientation interaction predicting transgender bathroom choice moral conviction from Study 19. Political orientation is centered on moderates, 1 *SD* below moderate = liberals, 1 *SD* above moderate = conservatives. Ribbons indicate standard error.

Table S19.22

*Simple Slopes for the Religious Conviction by Political Orientation Interaction Predicting Transgender Bathroom Choice Moral Conviction*

|  | Religious Conviction Predicting Moral Conviction | | | |
| --- | --- | --- | --- | --- |
| Level of Moderator | *B* | *SE* | *t* | *p* |
| Liberals  (1 *SD* below moderate) | 0.15 | 0.11 | 1.45 | .15 |
| Moderates | 0.32 | 0.07 | 4.32 | < .001 |
| Conservatives  (1 *SD* above moderate) | 0.49 | 0.08 | 5.79 | < .001 |

*Note.* df = 406.

# References

Brandt, M. J., Wisneski, D. C., & Skitka, L. J. (2015). Moralization and the 2012 U.S. presidential election campaign. *Journal of Social and Political Psychology, 3*, 211-237.

Hanson, B. E. & Skitka, L. J. (2013). *The “culture wars” in everyday life: Investigating moral conflict in anticipated social interactions.* Poster presented at the annual meeting of the Society for Personality and Social Psychology, New Orleans, LA.

Hanson, B. E., Skitka, L. J., & Wisneski, D. C. (2016). *Moral conviction’s limit on Supreme Court legitimacy: A natural experiment involving same-sex marriage*. Poster presented at the annual meeting of the Society for Personality and Social Psychology, San Diego, CA.

Mallett, R. K., Washburn, A. N., & Skitka, L. J. (under review). *Victim, honor, dignity, or activism? Preferred responses to politically motivated insults.*

Mueller, A. B. (2016). Effects of perspective taking on moral and religious conviction. Unpublished raw data.

Plante, T. G., & Boccaccini, M. (1997). The Santa Clara Strength of Religious Faith Questionnaire. *Pastoral Psychology, 45,* 375–387.

Reifen-Tagar, M., Morgan, G. S., Halperin, E., & Skitka, L. J. (2013). When ideology matters: Moral conviction and the association between ideology and policy preferences in the Israeli-Palestinian conflict. *European Journal of Social Psychology, 44*, 117-125.

Skitka, L. J., Bauman, C. W., & Lytle, B. L. (2009). The limits of legitimacy: Moral and religious convictions as constraints on deference to authority. *Journal of Personality and Social Psychology, 97,* 567 - 578*.*

Skitka, L. J., Bauman, C. W., & Sargis, E. G. (2005). Moral conviction: Another contributor to attitude strength or something more? *Journal of Personality and Social Psychology, 88,* 895 – 917.

Skitka, L. J., Hanson, B. E., & Wisneski, D. C. (2017). Utopian hopes or dystopian fears? Exploring the motivational underpinnings of moralized political engagement. *Personality and Social Psychology Bulletin, 43*, 177-190.

Skitka, L. J., & Morgan, G. S. (2014). The social and political implications of moral conviction. *Advances in Political Psychology, 35*, 95-110.

Washburn, A. N., & Skitka, L. J. (2014). [Moral conviction and science denial]. Unpublished raw data.

Washburn, A. N., & Skitka, L. J. (2015). [Consensus and science denial]. Unpublished raw data.

Washburn, A. N., & Skitka, L. J. (2017). Science denial across the political divide: Liberals and conservatives are similarly motivated to deny attitude-inconsistent science. *Social Psychological and Personality Science.* doi: 10.1177/1948550617731500

1. Sample sizes sometimes vary slightly across issues within the same study, however, due to different patterns of missingness for variables included in the analysis, due to some examples of people not completing any measures for a given issue (e.g., the participant dropped out before completing the entire survey). [↑](#footnote-ref-1)
